# Physiologically Based Pharmacokinetic (PBPK) Modeling of FIX in Pediatric Hemophilia B: Extravascular Distribution and Dosing Optimization

**DOI:** 10.3390/pharmaceutics18081030

**Published:** 2026-08-20

**Authors:** Mengmeng Liu, Guoqing Liu, Qixian Ling, Yongbo Chen, Haojie Xu, Runhui Wu, Zhenping Chen, Libo Zhao

**Affiliations:** 1 Department of Pharmacy, Peking University Third Hospital, Beijing 100191, China; 2511210484@bjmu.edu.cn (M.L.); 15155493869@163.com (Q.L.); 18868284537@163.com (Y.C.); x17852205820@163.com (H.X.); 2 Hemophilia Comprehensive Care Center, Hematology Department, Beijing Children’s Hospital, Capital Medical University, National Center for Children’s Health, Beijing 100045, China; 18810324215@163.com (G.L.); runhuiwu@hotmail.com (R.W.)

**Keywords:** hemophilia B, PBPK modeling, factor IX, pediatric population, extravascular distribution, dose optimization

## Abstract

**Background**: Prophylaxis in children with hemophilia B (HB) lacks quantitative approaches that integrate both plasma exposure and tissue distribution. This study aimed to develop and validate a physiologically based pharmacokinetic (PBPK) model of factor IX (FIX) for pediatric HB. The model incorporated the binding of FIX to type IV collagen (Col4) to characterize its distribution in both plasma and extravascular tissues. **Methods**: A total of 20 children with severe HB were included, contributing 219 plasma samples. The base PBPK model was first established and verified using adult and plasma-derived FIX (pdFIX) data. It was subsequently extrapolated to children by integrating FIX-CTBB parameters and pediatric observations for model calibration. The validated model was used to characterize plasma pharmacokinetics, predict tissue distribution and target attainment, and simulate alternative prophylactic dosing regimens. **Results**: The model adequately described the plasma pharmacokinetics of FIX in children and predicted substantial extravascular distribution. Total extravascular exposure was approximately sixfold higher than plasma exposure. Marked heterogeneity in target attainment was identified across tissues. Lower target attainment was observed in the colon, pancreas, and brain, whereas delayed attainment occurred in bone and muscle. Simulations of prophylactic dosing regimens suggested that 75 IU/kg twice weekly may provide a favorable balance among sustained FIX exposure, tissue-level target attainment, and treatment burden. **Conclusions**: This PBPK model provides a mechanistic and quantitative framework for characterizing plasma and tissue exposure to FIX in children with HB and may support individualized optimization of FIX prophylactic dosing.

## 1. Introduction

Hemophilia B (HB) is a congenital X-linked bleeding disorder caused by pathogenic variants in F9, leading to factor IX (FIX) deficiency or dysfunction [1,2]. The prevalence of HB in males is approximately 3.8 per 100,000, with a birth prevalence of about 5.0 per 100,000 [3,4]. In children with HB, the goals of prophylaxis have extended beyond reducing major bleeding episodes. Current treatment aims to achieve sustained bleed control, preserve musculoskeletal function, reduce the cumulative risk of hemophilic arthropathy, and improve long-term quality of life. Available evidence [5,6] shows that regular prophylaxis can lessen bleeding-related morbidity and support favorable long-term functional outcomes. Although gene therapy [7] and non-factor [8,9] therapies can reduce injection frequency, their applicability remains limited. FIX replacement therapy therefore continues to serve as the cornerstone of treatment.

At present, FIX replacement therapy in children with HB is still guided primarily by plasma FIX activity, which serves as the key metric for dose and dosing frequency adjustment. This strategy is clinically practical and widely applicable. However, its evidence base remains incomplete. Existing pediatric studies [10,11] have provided important support for the safety and efficacy of prophylactic FIX therapy, yet direct comparative evidence remains insufficient for tuning and optimizing alternative regimens. Children also show marked developmental differences in body fluid composition, organ blood flow, tissue distribution, and drug disposition [12,13]. As a result, dosing regimens established in adults cannot be directly extrapolated to pediatric patients by simple body weight adjustment alone.

Previous studies [14,15] have shown that no single target trough level is appropriate for all patients. Some patients maintain good bleed control at relatively low trough levels, whereas others may still experience bleeding despite achieving higher target levels. In addition, the in vivo behavior of FIX is not confined to the intravascular space. Available evidence [16,17] indicates that FIX binds specifically to type IV collagen (Col4) in the extravascular space, where a substantial extravascular reservoir is present. Competitive displacement experiments in primates suggested that the extravascular FIX pool may be at least three times larger than the circulating pool and is maintained through rapid and reversible exchange with plasma [18]. In hemophilia B mouse models, FIX exerted hemostatic effects even when plasma FIX was not measurable, further supporting the potential functional relevance of extravascular FIX [19,20]. Although plasma FIX activity remains an important marker, it may not fully capture actual exposure or the degree of protection achieved in different tissues. This issue is particularly relevant in pediatric patients, in whom tissue physiology, perfusion patterns, and extravascular space characteristics are all changing over time, which may further influence the distribution of FIX across anatomical sites. In addition, pediatric patients tend to have higher levels of physical activity and remain at increased risk of bleeding, especially in joints and muscles, which are closely linked to long-term functional outcomes. Therefore, defining tissue FIX exposure may be critical for optimizing prophylactic strategies and improving long-term clinical outcomes in children with HB.

To address these clinical and methodological needs, this study aimed to develop and validate a PBPK model of FIX therapy for children with HB, with the goal of systematically characterizing plasma exposure and tissue distribution. On this basis, the model was further applied to compare in vivo exposure and tissue target attainment across dosing regimens that differed in dose level, dosing frequency, and timing of administration, thereby evaluating more effective prophylactic strategies for pediatric patients. We expect this work to provide a mechanistically grounded quantitative framework for individualized optimization of FIX dosing in children with HB and to inform future pediatric dose selection and model-informed decision making.

## 2. Materials and Methods

### 2.1. Basic PBPK Model Development

**Figure 1:**
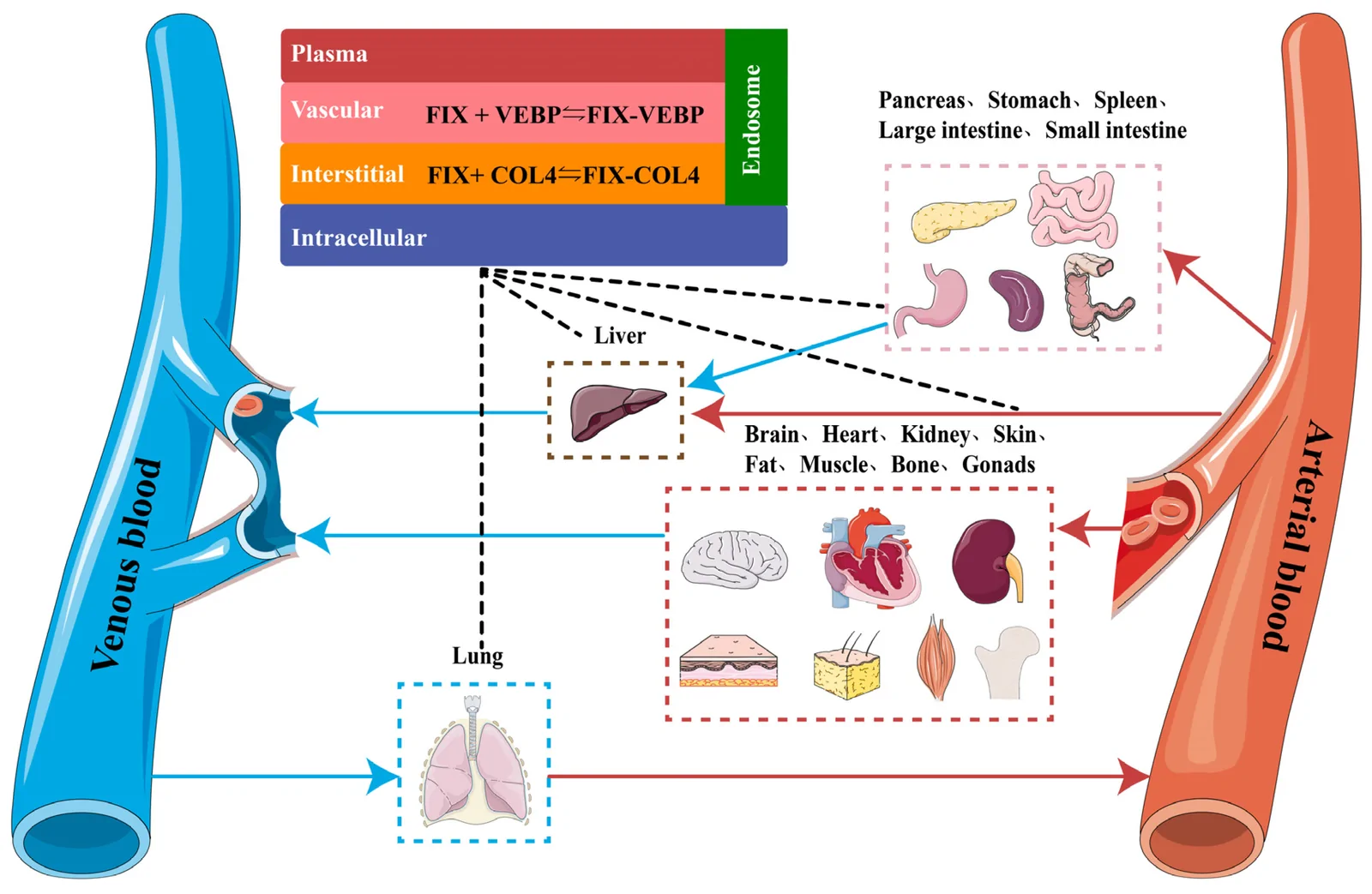
Schematic representation of the physiologically based pharmacokinetic (PBPK) model. Each organ compartment consists of a vascular space (red), containing plasma and blood cells, and an interstitial space (orange), which includes type IV collagen (Col4). FIX binds reversibly to Col4 in the extravascular space, and reversibly to a vascular endothelial binding partner (VEBP) within the vascular space.

The PBPK model describing FIX therapy in children with HB was developed on the basis of a previously reported PBPK model for macromolecules [21], which was used to characterize the pharmacokinetic behavior of intravenously administered FIX in human plasma and tissues. The model comprised 15 organ compartments representing a virtual human body. Each tissue compartment was further divided into plasma, vascular endothelial, endosomal, interstitial fluid (or extravascular), and intracellular subcompartments (Figure 1). At the organ level, transcapillary exchange was described using the two-pore model, and FIX in the interstitial space returned to the systemic circulation through lymphatic flow. Because FIX does not contain an Fc moiety, FcRn-mediated binding and recycling were not included. The complete mass-balance equations are provided in Supplementary Method S1.

To develop the PBPK model for FIX, physicochemical parameters of plasma-derived factor IX (pdFIX) from Drugbank and relevant clinical observation data were collected. To capture the extravascular distribution of FIX, Col4 was introduced as a binding component in the extravascular space. Information on Col4 expression across tissues was obtained from the PK-Sim^®^ expression database [22]. Previous studies have suggested that FIX may also bind to the vascular endothelium. Following the modeling approach reported by Michael [23], a vascular endothelial binding partner (VEBP) was further introduced into the vascular space to improve the description of FIX binding. The catalytic rate constant, K_cat_, was used to characterize FIX degradation and elimination in vivo. Based on the clinical plasma concentration data of pdFIX, the model was calibrated by parameter identification using a Monte Carlo approach combined with sensitivity analysis to estimate the binding parameters of FIX to Col4 and VEBP.

### 2.2. Pediatric Dataset for Model Validation

Children with severe HB were enrolled at Beijing Children’s Hospital. Eligibility criteria were as follows: age 1–16 years, a confirmed diagnosis of severe HB (factor IX activity ≤2 IU/dL), and ongoing prophylactic treatment with human coagulation Factor IX-CTBB (FIX-CTBB). Exclusion criteria included the presence of a FIX inhibitor (anti-FIX antibody titer > 0.6 Bethesda units/mL), active bleeding, or any other coagulation disorder. The study protocol was approved by the Ethics Committee of Beijing Children’s Hospital and was conducted in accordance with the Declaration of Helsinki. Written informed consent was obtained from all enrolled children and their legal guardians. The study was registered in the Chinese Clinical Trial Registry (ChiCTR; registration number: ChiCTR2200059494).

After a washout period of at least 96 h, each child received a single infusion of FIX-CTBB at 50 ± 5 IU/kg. Peripheral venous blood was collected into two 2 mL vacuum tubes containing 3.2% trisodium citrate. As described previously [24], samples were obtained before dosing and at 15 min, 30 min, 1 h, 3 h, 6 h, 9 h, 24 h, 48 h, 72 h, and 96 h after infusion. Immediately after collection, all blood samples were centrifuged at 2500× *g* for 15 min at room temperature (20–25 °C) to obtain platelet-poor plasma. The plasma was then stored at −80 °C until further analysis.

FIX activity (FIX:C) was measured using a one-stage clotting assay based on activated partial thromboplastin time (APTT), with measurements performed at multiple dilutions. HemosIL FIX-deficient plasma and HemosIL^®^ SynthAsil reagents were used (Instrumentation Laboratory, Bedford, MA, USA). FIX inhibitor titers were determined using the Nijmegen-modified Bethesda assay. All assays were performed on an ACL TOP 700 automated coagulation analyzer (Instrumentation Laboratory, Bedford, MA, USA). FIX inhibitor testing was conducted for all samples at baseline and at each post-dose time point.

### 2.3. Model Extrapolation and Validation

The drug-specific parameters of FIX-CTBB were then incorporated into the base model. The collected pediatric clinical data were split into a training set and a validation set at a ratio of 8:2 [25,26]. The training set was used to repeat the parameter identification process and extrapolate the adult model to the pediatric population for simulation of FIX disposition in children. During pediatric extrapolation, the adult model structure, organ-compartment topology, transcapillary transport equations, lymphatic return, and literature-derived Col4 and VEBP binding parameters were retained. Age-dependent anatomical and physiological variables, including body size, organ volumes, blood flows, hematocrit, and interstitial-space volumes, were generated using the pediatric physiology framework in PK-Sim^®^. The pediatric training dataset was used to re-estimate $K_{cat}$ and the Col4 reference concentration, whereas $K_{D,Col4}$, $K_{D,VEBP}$ and the VEBP concentration remained fixed at their literature-derived values. Based on the established pediatric PBPK model, age-stratified simulations were further performed in children aged 1–2, 2–6, 6–12, and 12–18 years. To evaluate the predictive performance of the PBPK model, simulated results were compared with the validation set from the pediatric clinical data. A virtual pediatric population of 1000 subjects aged 1 to 18 years was generated on the basis of the model, with variability in height and body weight.

Quantitative predictive performance was additionally assessed by matching each observed FIX concentration to the central model prediction and the lower and upper limits of the 95% prediction interval at the same nominal sampling time [27]. The percentage prediction error (PE) for each observation was calculated according to Equation (1):

(1) $${PE}_{i}(\%)=\frac{\left(C_{pred,i}-C_{obs,i}\right)}{C_{obs,i}}\times 100$$

where $C_{pred,i}$ and $C_{obs,i}$ represent the model-predicted and observed FIX concentrations for the observation, respectively. Positive PE values indicate overprediction, whereas negative values indicate underprediction. Model bias and predictive precision were summarized using the mean prediction error (MPE), mean absolute percentage error (MAPE), root mean squared error (RMSE), and average absolute fold error (AAFE). These metrics were calculated according to Equations (2)–(5):

(2) $$MPE(\%)=\frac{1}{N}\sum_{i=1}^{N}{PE}_{i}$$

(3) $$MAPE(\%)=\frac{1}{N}\sum_{i=1}^{N}\left|{PE}_{i}\right|$$

(4) $$RMSE=\sqrt{\frac{1}{N}\sum_{i=1}^{N}{\left(C_{pred,i}-C_{obs,i}\right)}^{2}}$$

(5) $$AAFE=10^{\left[\frac{1}{N}\sum \left|\log_{10}(\frac{C_{pred,i}}{C_{obs,i}})\right|\right]}$$

A local sensitivity analysis was performed within the Open Systems Pharmacology framework. Each selected parameter was varied around its final value using a variation range of 0.1 and two perturbation steps in each direction, while all other parameters were held constant. Sensitivity was evaluated for plasma AUC, half-life, clearance, volume of distribution, and selected tissue-exposure endpoints. The full sensitivity equation and perturbation settings are provided in Supplementary Method S2.

### 2.4. Pediatrics Dose Optimization

The established pediatric PBPK model for FIX was used to evaluate dose level and dosing frequency. Simulations were performed using 50 IU/kg as the reference regimen, with two additional dose levels of 75 IU/kg and 100 IU/kg. Dosing frequency was simulated as once, twice, or three times weekly. In addition, 50 IU/kg twice weekly was compared with 100 IU/kg once weekly to assess the effects of dose level and dosing frequency under the same total weekly dose. Target thresholds of 1%, 3%, and 5% were selected as exploratory tissue-reference levels. The 1% and 5% cutoffs correspond to the conventional plasma FIX activity boundaries separating severe, moderate, and mild hemophilia [28], consistent with previous FIX pharmacokinetic modeling that evaluated target attainment across multiple predefined FIX activity levels [29]. Because these thresholds were originally established for plasma FIX activity, their application to tissues was intended primarily for comparative model evaluation, and their clinical relevance as tissue-protection thresholds requires further validation. These thresholds correspond to clinically relevant levels, with increasing FIX activity associated with reduced bleeding risk and reflecting the transition from severe to moderate and mild HB phenotypes. Given that the normal plasma FIX concentration is approximately 5 μg/mL and the molecular weight is 55 kDa, these thresholds corresponded to 0.909 nM, 2.727 nM, and 4.545 nM, respectively [30]. Duration above target and target attainment rate at each threshold were used as evaluation metrics to identify the better dosing regimen associated with the overall clinical benefit in the pediatric population.

### 2.5. Software

Whole-body PBPK models for pdFIX and FIX-CTBB were developed using PK-Sim^®^ and MoBi^®^ (version 12.1; Open Systems Pharmacology). Plasma concentration–time data for the pdFIX base model were extracted from published studies using Engauge Digitizer (version 12.1). Data analysis and figure preparation were performed using R (version 4.4.3) and RStudio (version 2024.12.1+563).

## 3. Results

### 3.1. Population Characteristics

A total of 20 pediatric patients were included, contributing 219 plasma samples. All patients had FIX:C levels below 2%. The mean age was 8.59 ± 4.46 years (range, 1.42–16.66 years), mean body weight was 35.67 ± 18.77 kg (range, 12.00–70.00 kg), and mean height was 134.30 ± 29.85 cm (range, 107.50–148.00 cm). All patients tested negative for FIX inhibitors (<0.60 BU/mL). Detailed demographic characteristics are summarized in Table S1.

### 3.2. Development and Optimization of FIX PBPK Models in Adults and Pediatric Patients

**Figure 2:**
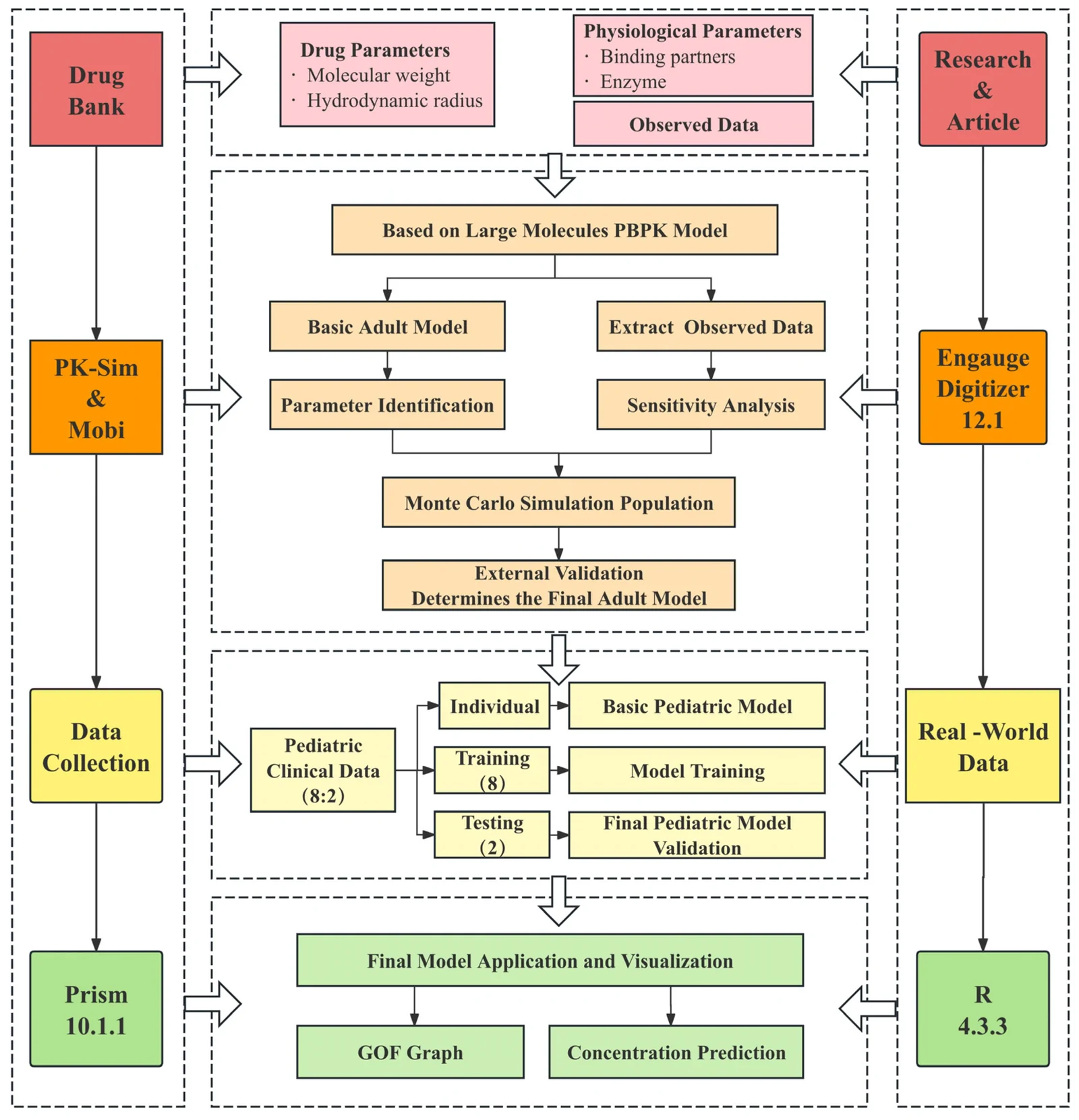
Workflow for developing a PBPK model of FIX therapy in pediatric HB. PBPK, physiologically based pharmacokinetic; GOF, goodness-of-fit diagnostics used for model evaluation.

**Figure 3:**
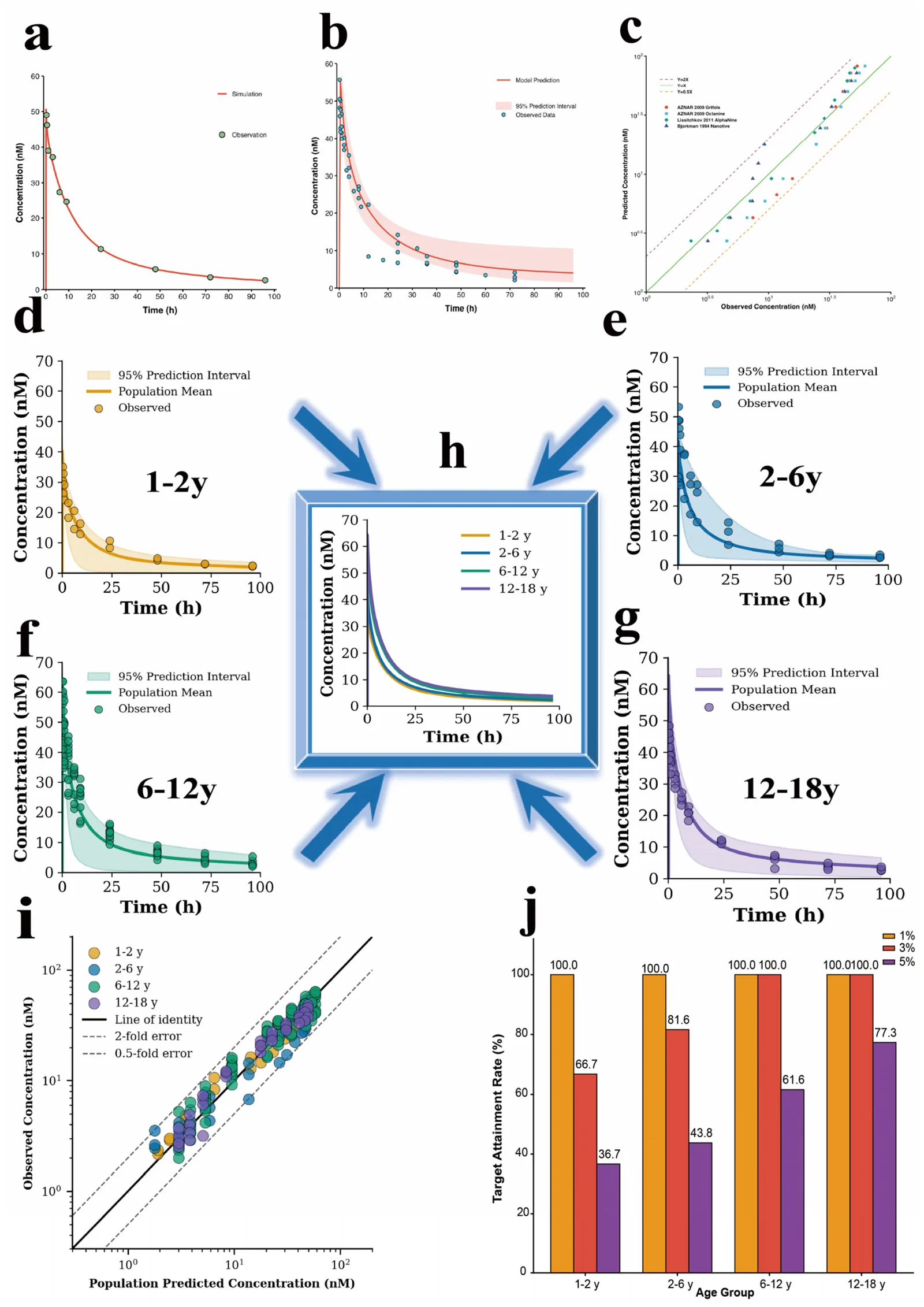
Development and validation of the PBPK model for FIX in adults and children. (**a**) Individual fit of the adult PBPK model. The line represents model-simulated FIX concentrations, and dots represent observed data. (**b**) Population fit of the adult PBPK model for plasma FIX concentrations. The line represents the model-predicted profile, the shaded area indicates the 95% prediction interval, and dots represent observed data from published studies. (**c**) Predicted versus observed FIX concentrations in adults. The solid line represents the line of identity (y = x), and the dashed lines indicate twofold deviation (y = 0.5x and y = 2x). Symbols denote data from different studies [31,32,33], with most observations distributed within the twofold error range. Population fit of the pediatric PBPK model using the 1–2 years (**d**), 2–6 years (**e**), 6–12 years (**f**) and 12–18 years (**g**) dataset. The line represents the model-predicted profile, the shaded area indicates the 95% prediction interval, and symbols represent observed data. (**h**) Mean plasma FIX concentration–time profiles across pediatric age groups after a single 50 IU/kg dose. The orange, blue, green, and purple curves represent children aged 1–2, 2–6, 6–12, and 12–18 years, respectively. (**i**) Predicted versus observed FIX concentrations in children. The solid line represents the line of identity (y = x), and the dashed lines indicate twofold deviation (y = 0.5x and y = 2x), with most observations within the twofold error range. (**j**) Bar plots showing the proportion of pediatric patients achieving predefined FIX activity targets across age groups. The orange, red, and purple bars indicate target thresholds of >1%, >3%, and >5%, respectively.

A PBPK model of FIX therapy in children with HB was developed using PK-Sim, and the overall workflow is shown in Figure 2. After incorporation of FIX binding to Col4, an adult-based PBPK model was established and then extrapolated to the pediatric population. The model was further refined by fitting a reference plasma concentration–time profile and estimating drug parameters related to elimination and distribution. The individual fit in adults is shown in Figure 3a. The adult-based model was then evaluated using published observational data. The predicted profiles from 1000 virtual European adult males showed good agreement with the observed data (Figure 3b,c). The adult model was subsequently extrapolated to children to estimate pediatric parameters related to elimination and distribution. Model parameters were further optimized using the training dataset, and simulations were performed in 1000 virtual Asian pediatric subjects aged 1–18 years to establish the final pediatric PBPK model. Age-stratified simulations were subsequently conducted in four pediatric subgroups: 1–2 years (Figure 3d), 2–6 years (Figure 3e), 6–12 years (Figure 3f), and 12–18 years (Figure 3g). In all age groups, the simulated population mean concentration–time profiles were in good agreement with the observed FIX concentrations, with most observations falling within the corresponding 95% prediction intervals. The goodness-of-fit plot further showed that the observed concentrations were generally consistent with the population-predicted concentrations across the pediatric age range (Figure 3i). In the testing dataset, the MPE, MAPE, RMSE, and AAFE were 0.02%, 31.57%, 9.11 nM, and 1.421, respectively; 82.05% of observations fell within the 95% prediction interval, and 87.18% were within the twofold error range (Table S2). These results demonstrated that the final pediatric PBPK model adequately characterized FIX disposition in children aged 1–18 years.

### 3.3. Parameter Sensitivity Analysis

A sensitivity analysis was further performed to identify the key model parameters affecting the plasma pharmacokinetics of FIX in children. The results showed that area under the plasma concentration–time curve (AUC), half-life, and volume of distribution in plasma were more sensitive to changes in FIX radius and surface area (SA), whereas clearance (CL) was more sensitive to changes in fat and Col4. In contrast, VEBP binding had a limited effect on the plasma pharmacokinetic parameters of FIX. Detailed sensitivity results are provided in Figure S1.

### 3.4. PBPK Model Predictions of FIX Concentrations in Plasma and Tissues of Extravascular Space

The PBPK model was able to characterize the distribution of FIX in plasma and the extravascular space (Figure S2). The plasma pharmacokinetic parameters are summarized in Table S3. In adults, the plasma CL of FIX was 3.60 mL/h/kg, the steady-state volume of distribution (Vss) was 163.83 mL/kg, and the estimated terminal half-life was 41.36 h. In pediatric subjects, age-dependent differences in FIX exposure and disposition were observed. The youngest children showed higher body weight-normalized clearance, with CL values of 7.02 and 5.44 mL/h/kg in the 1–2 and 2–6 years age groups, respectively, compared with 5.24 and 3.03 mL/h/kg in the 6–12 and 12–18 years age groups. Consistently, systemic exposure increased with age, as reflected by AUC values of 570, 641, 853, and 940 nM·h in the 1–2, 2–6, 6–12, and 12–18 years groups, respectively. Although C_max_ was lower in younger children than in older pediatric subjects, all pediatric age groups achieved 100% plasma target attainment at the 1% FIX activity threshold. However, clear age-related differences were observed at higher target thresholds. The 3% target attainment rate increased from 66.7% in children aged 1–2 years and 81.6% in those aged 2–6 years to 100% in both the 6–12 and 12–18 years groups. Similarly, the 5% target attainment rate was lower in younger children, reaching 36.7% and 43.8% in the 1–2 and 2–6 years groups, respectively, compared with 61.6% and 77.3% in the 6–12 and 12–18 years groups. These findings indicate that, despite complete attainment of the conventional 1% trough target, younger pediatric patients may have insufficient FIX exposure when higher prophylactic targets are considered, supporting the need for age-specific evaluation and dose optimization in children with hemophilia B. Additional model-derived parameters are provided in Table 1.

Table 2 further summarizes the distribution of FIX in children. In plasma, the AUC_0–96h_ was 916.24 nmol·h/L, the peak concentration was 51.2 nM, and the half-life was 46.35 h. Total extravascular exposure was approximately sixfold higher than plasma exposure, indicating substantial extravascular distribution of FIX. Among tissues, the spleen, liver, and kidney showed AUC_0−96h_ values of 832.06, 824.14, and 661.77 nmol·h/L, respectively, suggesting that these tissues may represent major sites of extravascular FIX distribution.

**Figure 4:**
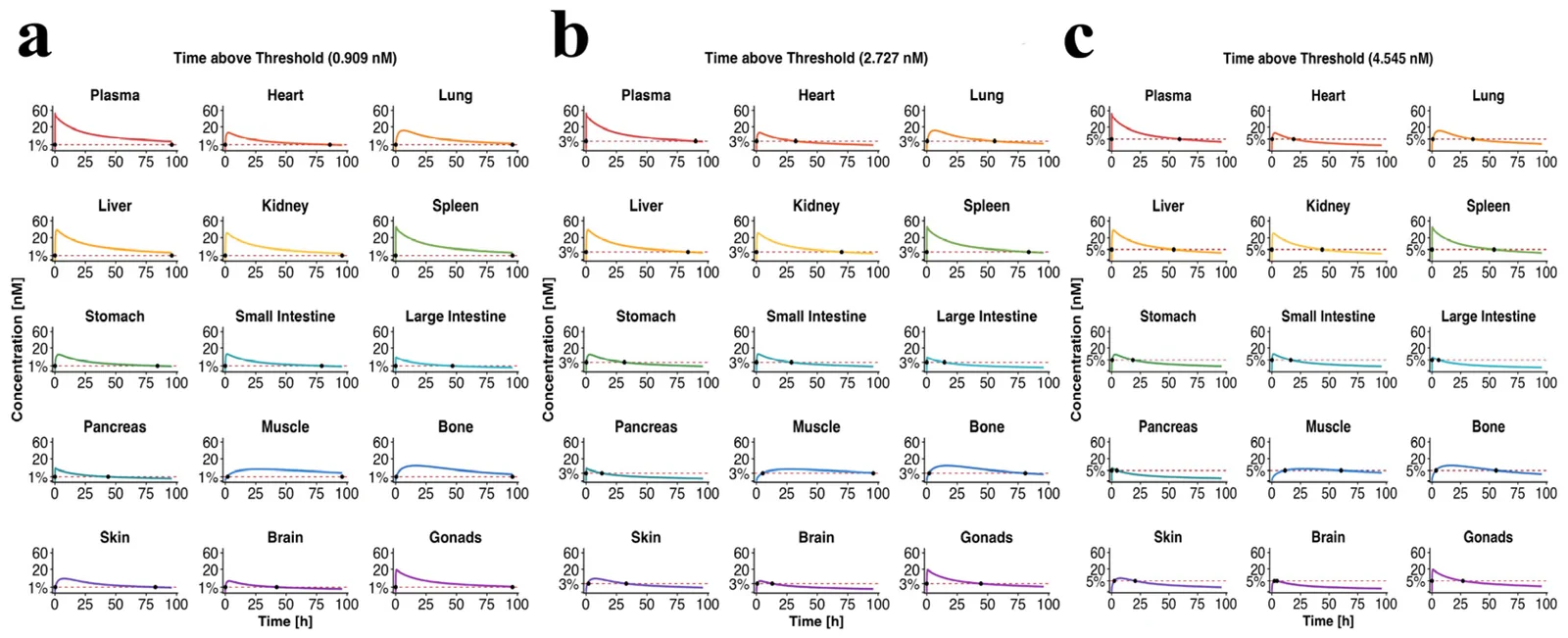
Time above threshold of FIX concentrations across tissues in children. (**a**) Threshold of 1% (0.909 nM). (**b**) Threshold of 3% (2.727 nM). (**c**) Threshold of 5% (4.545 nM). The red dashed line indicates the corresponding concentration threshold. Black dots indicate the time points at which FIX concentrations reach the target.

Target attainment of FIX across tissues is shown in Figure 4, and the corresponding time-above-target and target attainment rates are provided in Table S4. Using a threshold of FIX concentration >1%, target attainment exceeded 80.00% in plasma and in most organs and tissues. In contrast, target attainment was lower in the large intestine, pancreas, and brain, at 48.65%, 45.52%, and 43.80%, respectively. In addition, the time to reach 1% FIX concentration was delayed in bone and muscle, at 0.55 h and 2 h, respectively, compared with other tissues. When higher thresholds of >3% or >5% were applied, overall target attainment decreased further. Consistent with the results at the 1% threshold, the colon, pancreas, and especially the brain continued to show the lowest target attainment, and delayed attainment in bone and muscle remained evident.

### 3.5. Dose Optimization

**Figure 5:**
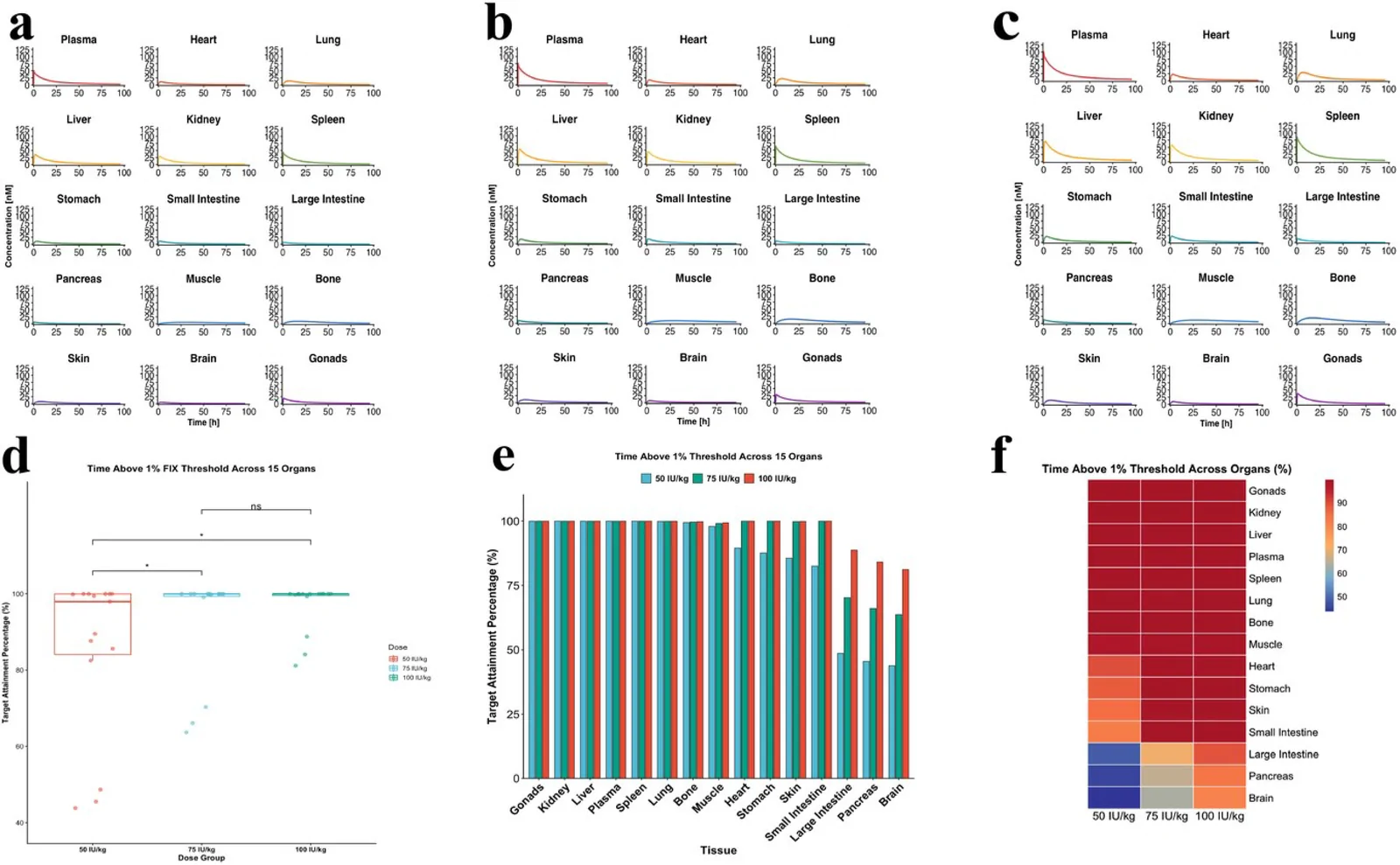
Effect of dose level on FIX exposure and target attainment in children. (**a**) Tissue concentration–time profiles of FIX after administration of 50 IU/kg. (**b**) Tissue concentration–time profiles of FIX after administration of 75 IU/kg. (**c**) Tissue concentration–time profiles of FIX after administration of 100 IU/kg. (**d**) Box plots of target attainment rates (>1%) at different dose levels. Red represents the 50 IU/kg group, blue represents the 75 IU/kg group, and green represents the 100 IU/kg group. The 75 IU/kg and 100 IU/kg groups showed higher target attainment than the 50 IU/kg group, with no significant difference between 75 IU/kg and 100 IU/kg, * *p* < 0.05. (**e**) Bar plots of target attainment rates (>1%) at different dose levels. Blue represents 50 IU/kg, green represents 75 IU/kg, and red represents 100 IU/kg. (**f**) Heatmap of target attainment rates (>1%) at different dose levels. Colors range from blue to red, indicating increasing target attainment. From left to right, the panels represent the 50 IU/kg, 75 IU/kg, and 100 IU/kg dose groups.

The simulations showed differences between plasma and tissue FIX exposure, and target attainment varied across pediatric age groups (Figure 3j). Therefore, additional dose-exploration simulations were conducted to evaluate dosing strategies in children with hemophilia B. The simulation results for different dose levels are shown in Figure 5. Figure 5a–c present the distribution of FIX across tissues after administration of 50 IU/kg, 75 IU/kg, and 100 IU/kg, respectively. The distribution profiles suggested that, in some organs or tissues such as muscle, further dose escalation produced only limited improvement in tissue exposure, indicating potential saturation of FIX distribution in the extravascular space. Target attainment rates at the threshold of FIX concentration >1% across dose groups are shown in Figure 5d. Both the 75 IU/kg and 100 IU/kg groups showed significantly higher target attainment than the 50 IU/kg group, whereas the difference between the 75 IU/kg and 100 IU/kg groups was not.

Bar plots and heatmaps of the corresponding target attainment results are also provided in Figure 5e,f. Detailed results for FIX concentrations >3% and >5% are provided in Figures S12–S17. The simulation results for different dosing frequencies are shown in Figures S30–S38. As dosing frequency increased, target attainment improved significantly at FIX concentration thresholds of >1%, >3%, and >5%. Further comparison of 50 IU/kg twice weekly and 100 IU/kg once weekly showed that the 50 IU/kg twice weekly regimen achieved better target attainment at the 1%, 3%, and 5% thresholds. These findings suggest that, during prophylactic FIX therapy in children with HB, increasing dosing frequency may provide more benefit than increasing the dose per infusion. Dosing time was also evaluated. Under the same twice-weekly regimen, administration of the second dose at 72 h showed better target attainment than administration at 96 h, but the mean target attainment rates (92.99% vs. 91.62%) were similar between the two schedules. Overall, the simulation results support 75 IU/kg twice weekly as a potentially preferred regimen for children with HB.

## 4. Discussion

Building on a previously reported adult PBPK model for macromolecules and integrating clinical observations from children with HB, this study developed and validated a pediatric PBPK model for FIX therapy. The model adequately characterized the plasma pharmacokinetics of FIX in children and further described its distribution in extravascular tissues. Using this model, FIX exposure under different dose levels, dosing frequencies, and dosing schedules was systematically compared, and pediatric prophylactic regimens were further evaluated and optimized. By incorporating the mechanistic basis of extravascular FIX binding, this study established a quantitative modeling framework that may support clinical dosing decisions and inform individualized FIX therapy in children with HB.

In this study, age-dependent differences in FIX disposition were observed between pediatric subjects and adults. This study showed that younger children had higher body weight-normalized CL than adults, with the highest CL observed in the 1–2 years group, followed by a gradual decrease with increasing age, and Vss was consistently higher in all pediatric age groups than in adults. Similar trends have been reported previously. In a population pharmacokinetic model of recombinant factor IX Fc fusion protein (rFIXFc) in children younger than 12 years, Koopman [34] found that increasing age was independently associated with lower CL. In a phase III pediatric study of Nonacog Beta Pegol (N9-GP) [35], children showed higher body weight-normalized CL and lower incremental recovery than adults and adolescents. Udata [36] developed a population pharmacokinetic model for rFIX and reported that the Vss was slightly higher in children than in adults. Terasakar [5] also developed a population pharmacokinetic model for rIX-FP and found that CL and Vss were higher in children and decreased with age. Drug distribution and elimination are jointly influenced by developmental changes in body composition, extracellular fluid fraction, organ size and blood flow, tissue perfusion, endothelial barrier properties, and protein and tissue binding [12]. Compared with adults, children generally have a larger extracellular fluid space relative to body size and continuously changing tissue composition [37], both of which may expand the apparent distribution space. Age-related differences in organ blood flow per kilogram and in the relative contribution of the liver may also increase CL for some drugs [38]. In this study, the higher CL and Vss observed in children may reflect age-dependent changes in FIX pharmacokinetics during growth and development. Unlike previous reports [34], the simulated terminal half-life was longer in children than in adults. This difference may be related to the specific FIX product studied and to metabolic differences between the populations.

Model predictions showed that total extravascular exposure in children exceeded plasma exposure, consistent with findings in adults [23]. These results further indicate that FIX distribution in children is not confined to the plasma compartment but involves substantial extravascular distribution. The concept of a distinct extravascular distribution pattern for FIX was proposed as early as the 1990s [16]. Based on competitive displacement experiments in primates, Feng [18] suggested that the extravascular FIX pool is at least three times larger than the circulating FIX pool, with a rapid and reversible equilibrium between plasma and extravascular FIX. In addition, Cooley [19,20] reported in a hemophilia B mouse model that FIX could still exert hemostatic effects even when no measurable FIX was detected in plasma, further supporting the presence of extravascular FIX distribution. The two binding components incorporated into the model are supported by previous experimental and modeling studies, but they differ in their level of mechanistic certainty. FIX binding to Col4 has been demonstrated experimentally and provides a biologically plausible mechanism for reversible extravascular retention [16,20]. Accordingly, the Col4 dissociation constant was adopted from previous studies, whereas the Col4 reference concentration was estimated during model optimization. In contrast, VEBP was introduced based on previous observations of specific and saturable FIX binding to vascular endothelial cells and was implemented as an operational endothelial binding pool rather than a molecularly identified receptor [20]. The VEBP dissociation constant and binding-partner concentration were therefore fixed to literature-derived values. These assumptions introduce uncertainty because the literature-derived affinities and binding capacities may not fully represent the pediatric population or tissue-specific endothelial environment, and the molecular identity and physiological abundance of VEBP remain incompletely characterized. Recent evidence [39] showing that VEGF/SDF-1α signaling regulates endothelial progenitor-cell migration, proliferation, differentiation, and vascular repair further illustrates the biological complexity and heterogeneity of the endothelial compartment. This complexity also supports the importance of incorporating an endothelial binding component into the PBPK framework, because transcapillary transport alone may not fully represent the specific and saturable interaction of FIX with the vascular endothelium. In the absence of sufficient pediatric data to independently identify parameters, retaining experimentally or previously model-informed parameter values provides a mechanistically constrained representation of this process and reduces parameter arbitrariness and the risk of overfitting to the limited pediatric dataset. Local sensitivity analysis showed that Col4-related parameters influenced selected pharmacokinetic endpoints, whereas VEBP-related parameters had relatively limited effects on plasma predictions. These findings support the overall stability of the model to moderate local parameter variation, while tissue-exposure predictions should still be interpreted in the context of the underlying Col4 and VEBP assumptions. The model predicted relatively high FIX exposure in the spleen, liver, and kidney, which may reflect organ-specific differences in perfusion, vascular and interstitial spaces, transcapillary transport, and tissue binding within the PBPK framework. A tissue distribution study in mice by Van [40] using SPECT/CT further showed that both rFIX and rFIXFc distributed beyond the plasma compartment and were retained in joint and muscle regions, with FIX-related signals also detected in the liver, spleen, and kidney. In addition, previous studies [41,42] found that, in tissue lysates from HB mice, the liver contained the highest level of FIX after dosing, while FIX was also detectable in the kidney and spleen. Thus, exposure in the liver, spleen, and kidney is primarily relevant to understanding whole-body FIX distribution and retention, whereas exposure in musculoskeletal tissues is more directly related to the clinical sites of bleeding. In this context, the delayed target attainment predicted in bone and muscle may be more clinically relevant to prophylactic protection than the higher AUC values observed in the liver, spleen, and kidney. Overall, these findings suggest that assessment of in vivo exposure based solely on plasma FIX levels may not fully reflect actual tissue retention or the potential hemostatic contribution of FIX at the tissue level.

Target attainment of FIX varied across tissues, and this pattern persisted at the 1%, 3%, and 5% thresholds. These findings indicate that FIX-mediated hemostatic protection is not determined by a single level of plasma exposure and shows clear spatial heterogeneity. In this study, target attainment was relatively low in the brain, large intestine, and pancreas. This may be related to organ-specific barrier properties, local perfusion patterns, and tissue microenvironment. In the brain, the blood–brain barrier tightly restricts the entry of macromolecules into the parenchyma through specialized receptor-mediated transport and active efflux systems, and the cerebral vasculature itself shows marked regional heterogeneity [43]. In the intestine, selective permeability is largely governed by epithelial tight junctions, and transport across the barrier is constrained by molecular size, charge, and available transport pathways [44]. Pancreatic blood supply is also regionally organized, with the endocrine and exocrine compartments sharing a complex microcirculatory network [45]. These factors may reduce FIX distribution in the brain, large intestine, and pancreas. These tissues with lower target attainment do not necessarily represent sites at highest risk of bleeding. They do indicate that FIX exposure is not distributed uniformly across tissues. Model simulations showed delayed target attainment of FIX in bone and muscle, a finding with direct clinical relevance. The major long-term disability burden in HB arises from recurrent joint bleeding and related musculoskeletal damage. Muscle bleeding can also lead to functional limitation, contracture, and even pseudotumor formation [46]. The slower establishment of FIX exposure in bone and muscle suggests that early tissue protection after a single dose may not be synchronized across tissues, which may contribute to the greater susceptibility of these sites to breakthrough bleeding. Adjusting the timing of physical activity after dosing in children according to this delay may help reduce the risk of bleeding in bone and muscle and may improve clinical benefit.

When the single dose was increased from 50 IU/kg to 75 IU/kg, target attainment improved clearly in most tissues. However, after further escalation to 100 IU/kg, the additional gain became limited in some tissues. These findings suggest that the distributional benefit of FIX in extravascular tissues may not continue to increase linearly with dose and may instead be constrained by a saturable process. A similar hypothesis has also been proposed in previous studies [47,48]. Under the same total weekly dose, model predictions showed that 50 IU/kg twice weekly performed better than 100 IU/kg once weekly. In a study of FIX by Valentino [49], the mean annualized bleeding rate (ABR) was lower with 50 IU/kg twice weekly than with 100 IU/kg once weekly (2.6 vs. 4.6), which is consistent with our findings. In the B-LONG study [10], prophylaxis with rFIXFc in children was initiated once weekly, but dosing frequency could be increased to twice weekly according to clinical need, further highlighting the distinct requirements of pediatric dosing strategies. However, the higher peak concentration achieved by a single high dose may not be sustained throughout the dosing interval. For prophylaxis in children with HB, the key consideration may be not a transiently higher peak, but the maintenance of adequate and stable effective exposure across the entire dosing interval. Under the same total weekly dose, dividing the dose into more frequent administrations may be more effective in maintaining sustained protection and may therefore provide better bleeding prevention in children. Although 75 IU/kg twice weekly improved tissue target attainment, this regimen would require a total weekly dose of 150 IU/kg, which is 50% higher than that required for either 50 IU/kg twice weekly or 100 IU/kg once weekly. Higher dose intensity would increase factor consumption and treatment burden [50,51]. However, more intensive prophylaxis may be clinically justified in selected pediatric patients because younger children generally exhibit higher body weight-normalized FIX clearance and lower systemic exposure than older children and adults. In addition, their higher levels of physical activity and the need to prevent recurrent musculoskeletal bleeding and cumulative joint damage may favor maintaining more sustained FIX exposure throughout the dosing interval. Optimization of prophylactic regimens in children requires a balance between sustained protection, injection burden, and long-term feasibility [52]. Accordingly, 75 IU/kg twice weekly should be considered a model-informed candidate for prospective clinical evaluation, with final dosing individualized according to clinical response, safety, adherence, and resource availability.

This study has several limitations. First, the pediatric model was extrapolated from an adult-based model, and several model parameters could not be independently identified from the pediatric data; therefore, the predictions remain conditional on the underlying structural and parameter assumptions. The conclusions regarding extravascular FIX distribution were derived from PBPK simulations and were not directly verified using tissue measurements or imaging data in children. Direct tissue sampling would be invasive and is generally neither ethically nor practically feasible in pediatric patients with hemophilia B. Although previous experimental and imaging studies provide biological support for extravascular FIX distribution, further experimental evidence will be valuable to validate the absolute tissue concentrations predicted by the present model. In addition, age-related and interindividual variability in organ size, blood flow, tissue volumes, endothelial permeability, and tissue-binding capacity may not have been fully captured, particularly for Col4- and VEBP-related processes. Second, the model was developed and internally evaluated using data from 20 pediatric patients recruited at a single center. Given the rarity of pediatric hemophilia B and the practical challenges associated with intensive pharmacokinetic sampling in children, collection of larger pediatric PK datasets remains challenging. Future studies should use multicenter datasets, additional FIX products, and prospectively collected clinical outcomes to further evaluate and extend the generalizability of the model to broader pediatric populations. Third, regimen optimization was based primarily on model-derived time-above-target and target attainment rates. These endpoints have not yet been validated against actual bleeding events, joint outcomes, safety outcomes, or long-term clinical benefit. Accordingly, the dosing regimens proposed in this study represent model-informed strategies, and their clinical application should be further individualized according to patient-specific characteristics, including clinical response, bleeding phenotype, safety, adherence, and treatment feasibility.

## 5. Conclusions

In conclusion, this study developed and validated a PBPK model of FIX for children with HB. The model suggests that FIX shows substantial extravascular distribution in children and that exposure and target attainment vary across tissues. In pediatric prophylaxis, increasing dosing frequency may be more effective than simply increasing the dose per infusion for maintaining sustained protection, and 75 IU/kg twice weekly may be a promising regimen. Further validation with larger multicenter studies and clinical outcome data will be needed to confirm the model predictions and the proposed dosing strategy.

## Figures and Tables

**Table 1:** Detailed parameters used in PBPK model development.

Parameter	Final Value	Reference
**FIX-CTBB**		
Molecular weight	55.00 kDa	Drugbank
Hydrodynamic radius	2.29 nm	Estimated
K_D_^Col4^	5.26 μM	Michael2024
K_D_^VEBP^	0.04 μM	Michael2024
**Enzyme**		
Enzymatic activity (K_cat_)	0.0057 1/day	Estimated
**Binding partners (concentration)**		
Col4	9.29 μM	Estimated
VEBP	20.00 μM	Michael2024

Abbreviations: FIX-CTBB, human coagulation factor IX-CTBB; KDCol4, dissociation constant for binding in extravascular space; KDVEBP, dissociation constant for vascular endothelium binding partner (VEBP).

**Table 2:** Plasma and tissue exposure of FIX after administration of 50 IU/kg.

Parameters	AUC_0–96h_ (nmol·h/L)	Peak FIX (nM)	T_max_ (h)	Half-Life (h)
**FIX intravascular**				
Plasma	916.24	51.20	0.05	46.35
**FIX extravascular**				
Bone	524.59	9.61	16.75	40.56
Brain	123.63	4.75	3.25	46.18
Gonads	404.23	19.11	0.75	46.32
Heart	281.85	11.33	2.50	46.23
Kidney	661.77	28.93	1.00	46.29
Stomach	272.88	10.52	3.00	46.19
Small Intestine	256.29	11.00	2.00	46.29
Large Intestine	141.80	7.11	0.40	46.33
Liver	824.14	35.37	2.00	46.28
Lung	474.44	14.59	6.50	45.81
Muscle	425.40	5.82	22.75	52.59
Pancreas	132.09	6.63	0.40	46.33
Skin	249.29	7.17	7.50	45.61
Spleen	832.06	42.55	0.30	46.34
**Total FIX in extravascular tissues**	5604.46			

## Data Availability

The raw data supporting the conclusions of this article will be made available by the authors upon request.

## Supplementary Materials

The following supporting information can be downloaded at https://www.mdpi.com/article/10.3390/pharmaceutics18081030/s1: Supplementary Method S1. PBPK Model Equations for FIX; Supplementary Method S2. Sensitivity Analysis; Supplementary Method S3. Statistical Comparison of Simulated Dosing Regimens; Figure S1. Sensitivity Analysis of Key Pharmacokinetic Parameters in the FIX PBPK Model; Figure S2. Extravascular Tissue Distribution of FIX in Children; Figure S3. Time Above the 1% FIX Target Across Tissues After Administration of 50 IU/kg; Figure S4. Time Above the 3% FIX Target Across Tissues After Administration of 50 IU/kg; Figure S5. Time Above the 5% FIX Target Across Tissues After Administration of 50 IU/kg; Figure S6. Time Above the 1% FIX Target Across Tissues After Administration of 75 IU/kg; Figure S7. Time Above the 3% FIX Target Across Tissues After Administration of 75 IU/kg; Figure S8. Time Above the 5% FIX Target Across Tissues After Administration of 75 IU/kg; Figure S9. Time Above the 1% FIX Target Across Tissues After Administration of 100 IU/kg; Figure S10. Time Above the 3% FIX Target Across Tissues After Administration of 100 IU/kg; Figure S11. Time Above the 5% FIX Target Across Tissues After Administration of 100 IU/kg; Figure S12. Box Plots of Tissue Target Attainment Rates (>3%) at Different Dose Levels; Figure S13. Bar Plots of Tissue Target Attainment Rates (>3%) at Different Dose Levels; Figure S14. Heatmap of Tissue Target Attainment Rates (>3%) at Different Dose Levels; Figure S15. Box Plots of Tissue Target Attainment Rates (>5%) at Different Dose Levels; Figure S16. Bar Plots of Tissue Target Attainment Rates (>5%) at Different Dose Levels; Figure S17. Heatmap of Tissue Target Attainment Rates (>5%) at Different Dose Levels; Figure S18. Tissue Distribution Profiles of FIX Under 50 IU/kg Once-Weekly Dosing; Figure S19. Time Above the 1% FIX Target Across Tissues Under 50 IU/kg Once-Weekly Dosing; Figure S20. Time Above the 3% FIX Target Across Tissues Under 50 IU/kg Once-Weekly Dosing; Figure S21. Time Above the 5% FIX Target Across Tissues Under 50 IU/kg Once-Weekly Dosing; Figure S22. Tissue Distribution Profiles of FIX Under 50 IU/kg Twice-Weekly Dosing; Figure S23. Time Above the 1% FIX Target Across Tissues Under 50 IU/kg Twice-Weekly Dosing; Figure S24. Time Above the 3% FIX Target Across Tissues Under 50 IU/kg Twice-Weekly Dosing; Figure S25. Time Above the 5% FIX Target Across Tissues Under 50 IU/kg Twice-Weekly Dosing; Figure S26. Tissue Distribution Profiles of FIX Under 50 IU/kg Three-Times-Weekly Dosing; Figure S27. Time Above the 1% FIX Target Across Tissues Under 50 IU/kg Three-Times-Weekly Dosing; Figure S28. Time Above the 3% FIX Target Across Tissues Under 50 IU/kg Three-Times-Weekly Dosing; Figure S29. Time Above the 5% FIX Target Across Tissues Under 50 IU/kg Three-Times-Weekly Dosing; Figure S30. Box Plots of Tissue Target Attainment Rates (>1%) at Different Dosing Frequencies; Figure S31. Bar Plots of Tissue Target Attainment Rates (>1%) at Different Dosing Frequencies; Figure S32. Heatmap of Tissue Target Attainment Rates (>1%) at Different Dosing Frequencies; Figure S33. Box Plots of Tissue Target Attainment Rates (>3%) at Different Dosing Frequencies; Figure S34. Bar Plots of Tissue Target Attainment Rates (>3%) at Different Dosing Frequencies; Figure S35. Heatmap of Tissue Target Attainment Rates (>3%) at Different Dosing Frequencies; Figure S36. Box Plots of Tissue Target Attainment Rates (>5%) at Different Dosing Frequencies; Figure S37. Bar Plots of Tissue Target Attainment Rates (>5%) at Different Dosing Frequencies; Figure S38. Heatmap of Tissue Target Attainment Rates (>5%) at Different Dosing Frequencies; Figure S39. Tissue Distribution Profiles of FIX Under 100 IU/kg Once-Weekly Dosing; Figure S40. Time Above the 1% FIX Target Across Tissues Under 100 IU/kg Once-Weekly Dosing; Figure S41. Time Above the 3% FIX Target Across Tissues Under 100 IU/kg Once-Weekly Dosing; Figure S42. Time Above the 5% FIX Target Across Tissues Under 100 IU/kg Once-Weekly Dosing; Figure S43. Box Plots of Tissue Target Attainment Rates (>1%) Comparing Increased Dosing Frequency With Dose Doubling at the Same Total Weekly Dose; Figure S44. Bar Plots of Tissue Target Attainment Rates (>1%) Comparing Increased Dosing Frequency With Dose Doubling at the Same Total Weekly Dose; Figure S45. Heatmap of Tissue Target Attainment Rates (>1%) Comparing Increased Dosing Frequency With Dose Doubling at the Same Total Weekly Dose; Figure S46. Box Plots of Tissue Target Attainment Rates (>3%) Comparing Increased Dosing Frequency With Dose Doubling at the Same Total Weekly Dose; Figure S47. Bar Plots of Tissue Target Attainment Rates (>3%) Comparing Increased Dosing Frequency With Dose Doubling at the Same Total Weekly Dose; Figure S48. Heatmap of Tissue Target Attainment Rates (>3%) Comparing Increased Dosing Frequency With Dose Doubling at the Same Total Weekly Dose; Figure S49. Box Plots of Tissue Target Attainment Rates (>5%) Comparing Increased Dosing Frequency With Dose Doubling at the Same Total Weekly Dose; Figure S50. Bar Plots of Tissue Target Attainment Rates (>5%) Comparing Increased Dosing Frequency With Dose Doubling at the Same Total Weekly Dose; Figure S51. Heatmap of Tissue Target Attainment Rates (>5%) Comparing Increased Dosing Frequency With Dose Doubling at the Same Total Weekly Dose; Figure S52. Tissue Distribution Profiles of FIX Under 50 IU/kg Twice-Weekly Dosing With the Second Dose Administered at 72 h; Figure S53. Time Above the 1% FIX Target Across Tissues Under 50 IU/kg Twice-Weekly Dosing With the Second Dose Administered at 72 h; Figure S54. Time Above the 3% FIX Target Across Tissues Under 50 IU/kg Twice-Weekly Dosing With the Second Dose Administered at 72 h; Figure S55. Time Above the 5% FIX Target Across Tissues Under 50 IU/kg Twice-Weekly Dosing With the Second Dose Administered at 72 h; Figure S56. Box Plots of Tissue Target Attainment Rates (>1%) Comparing 72-h and 96-h Second-Dose Schedules; Figure S57. Bar Plots of Tissue Target Attainment Rates (>1%) Comparing 72-h and 96-h Second-Dose Schedules; Figure S58. Heatmap of Tissue Target Attainment Rates (>1%) Comparing 72-h and 96-h Second-Dose Schedules; Figure S59. Box Plots of Tissue Target Attainment Rates (>3%) Comparing 72-h and 96-h Second-Dose Schedules; Figure S60. Bar Plots of Tissue Target Attainment Rates (>3%) Comparing 72-h and 96-h Second-Dose Schedules; Figure S61. Heatmap of Tissue Target Attainment Rates (>3%) Comparing 72-h and 96-h Second-Dose Schedules; Figure S62. Box Plots of Tissue Target Attainment Rates (>5%) Comparing 72-h and 96-h Second-Dose Schedules; Figure S63. Bar Plots of Tissue Target Attainment Rates (>5%) Comparing 72-h and 96-h Second-Dose Schedules; Figure S64. Heatmap of Tissue Target Attainment Rates (>5%) Comparing 72-h and 96-h Second-Dose Schedules; Table S1. Patients’ Baseline Characteristics and Demographics; Table S2. Quantitative Predictive Performance of the Pediatric FIX PBPK Model; Table S3. Plasma Pharmacokinetic Parameters in Adults and Children; Table S4. Time Above the 1% Target for FIX in Each Tissue in the Pediatric Model After Administration of 50 IU/kg; Table S5. Time Above the 3% Target for FIX in Each Tissue in the Pediatric Model After Administration of 50 IU/kg; Table S6. Time Above the 5% Target for FIX in Each Tissue in the Pediatric Model After Administration of 50 IU/kg; Table S7. Time Above the 1% Target for FIX in Each Tissue in the Pediatric Model After Administration of 75 IU/kg; Table S8. Time Above the 3% Target for FIX in Each Tissue in the Pediatric Model After Administration of 75 IU/kg; Table S9. Time Above the 5% Target for FIX in Each Tissue in the Pediatric Model After Administration of 75 IU/kg; Table S10. Time Above the 1% Target for FIX in Each Tissue in the Pediatric Model After Administration of 100 IU/kg; Table S11. Time Above the 3% Target for FIX in Each Tissue in the Pediatric Model After Administration of 100 IU/kg; Table S12. Time Above the 5% Target for FIX in Each Tissue in the Pediatric Model After Administration of 100 IU/kg; Table S13. Statistical Comparison of Target Attainment Metrics Between Different Dose Groups; Table S14. Time Above the 1% Target for FIX in Each Tissue in the Pediatric Model After Administration of 50 IU/kg Twice Weekly; Table S15. Time Above the 3% Target for FIX in Each Tissue in the Pediatric Model After Administration of 50 IU/kg Twice Weekly; Table S16. Time Above the 5% Target for FIX in Each Tissue in the Pediatric Model After Administration of 50 IU/kg Twice Weekly; Table S17. Time Above the 1% Target for FIX in Each Tissue in the Pediatric Model After Administration of 100 IU/kg Once Weekly; Table S18. Time Above the 3% Target for FIX in Each Tissue in the Pediatric Model After Administration of 100 IU/kg Once Weekly; Table S19. Time Above the 5% Target for FIX in Each Tissue in the Pediatric Model After Administration of 100 IU/kg Once Weekly; Table S20. Statistical Comparison of Target Attainment Between Increased Dosing Frequency and Increased Single-Dose Strategies Under the Same Total Weekly Dose; Table S21. Time Above the 1% Target for FIX in Each Tissue in the Pediatric Model After Administration of 50 IU/kg Three Times Weekly; Table S22. Time Above the 3% Target for FIX in Each Tissue in the Pediatric Model After Administration of 50 IU/kg Three Times Weekly; Table S23. Time Above the 5% Target for FIX in Each Tissue in the Pediatric Model After Administration of 50 IU/kg Three Times Weekly; Table S24. Statistical Comparison Metrics Between Different Dosing Frequency Groups; Table S25. Statistical Comparison Metrics Between Different Dosing Schedule Groups. Reference [53] is cited in the supplementary material.

## Abbreviations

The following abbreviations are used in this manuscript:

ABR	Annualized bleeding rate
APTT	Activated partial thromboplastin time
AUC	Area under the concentration–time curve
AUC_0–96h_	Area under the concentration–time curve from 0 to 96 h
BU	Bethesda unit
ChiCTR	Chinese Clinical Trial Registry
CL	Clearance
C_max_	Maximum concentration
Col4	Type IV collagen
FIX	Factor IX
FIX:C	Factor IX activity
FIX-CTBB	Human coagulation Factor IX-CTBB
GOF	Goodness of fit
HB	Hemophilia B
K_D_	Dissociation constant
K_DCol4_	Dissociation constant for binding to type IV collagen
K_DVEBP_	Dissociation constant for binding to vascular endothelial binding partner
K_cat_	Catalytic rate constant
N9-GP	Nonacog beta pegol
PBPK	Physiologically based pharmacokinetic
pdFIX	Plasma-derived factor IX
rFIX	Recombinant factor IX
rFIXFc	Recombinant factor IX Fc fusion protein
rIX-FP	Recombinant factor IX albumin fusion protein
SA	Surface area
SPECT/CT	Single-photon emission computed tomography/computed tomography
T_max_	Time to maximum concentration
VEBP	Vascular endothelial binding partner
V_ss_	Steady-state volume of distribution
