# Supplementary material for: Physiologically Based Pharmacokinetic (PBPK) Modeling of FIX in Pediatric Hemophilia B: Extravascular Distribution and Dosing Optimization
Source: Pharmaceutics. 2026 Aug 20;18(8):1030. doi: 10.3390/pharmaceutics18081030 (PMC13516121; doi:10.3390/pharmaceutics18081030)
Supplement: Supplementary file 1 [file pharmaceutics-18-01030-s001.zip › pharmaceutics-4465351-supplementary.pdf]

Supplementary Material

Supplement for: Physiologically Based Pharmacokinetic (PBPK) Modeling of FIX in Pediatric Hemophilia B: Extravascular Distribution and Dosing Optimization.

Contents

Supplementary Method 2

Supplementary Method S1. PBPK model equations for FIX 2

Supplementary Method S2. Sensitivity analysis 3

Supplementary Method S3. Statistical comparison of simulated dosing regimens 4

Supplementary Figures 5

Supplementary Tables 69

Reference 82

Supplementary Method

Supplementary Method S1. PBPK model equations for FIX

A whole-body PBPK model for FIX was developed in PK-Sim based on the generic therapeutic protein model described by Niederalt[1], with exclusion of FcRn-mediated binding and recycling because FIX does not contain an Fc moiety. The model therefore retained the structural components required for macromolecular distribution, including vascular, interstitial, and cellular spaces in each organ, as well as lymphatic return from the interstitial space to the systemic circulation.

For each organ, lymphatic return from the interstitial space to venous plasma was described as

$$J_{i,org}^{lymph} = L_{org} C_{i,org}$$

where $J_{i,org}^{lym}$ is the lymphatic mass flux from the interstitial space of organ org, L_{org} is organ lymph flow, and $C_{i,org}$ is the interstitial concentration. In the generic framework, lymph flow was parameterized as a fraction of organ plasma flow:

$$L_{org} = f_{lym\quad,org} Q_{blood,org} (1 - HCT)$$

where $f_{lymp\quad,org}$ is the organ-specific lymph flow fraction, $Q_{blood,org}$is organ blood flow, and HCT is hematocrit.

Transcapillary exchange from plasma to interstitial space was described using the two-pore model as the sum of convective and diffusive transport through small and large pores, parameterized by pore-specific

fluid flows, reflection coefficients, Peclet numbers, and permeability-surface area products. The latter were derived from solute radius, endothelial pore size, capillary surface area, hydraulic conductivity, and diffusion coefficient, the latter calculated according to the Stokes-Einstein relationship.

Because no Fc moiety was present, FcRn binding, endosomal recycling, and FcRn-mediated protection from intracellular degradation were not activated. Under this configuration, tissue distribution was driven primarily by macromolecular size, organ-specific endothelial properties, and lymphatic return.

Supplementary Method S2. Sensitivity analysis

Sensitivity analysis was conducted within the Open Systems Pharmacology (OSP) framework to characterize the influence of model input parameters on model-derived pharmacokinetic endpoints. In line with the OSP sensitivity-analysis workflow described by Najjar[2], a one-at-a-time local sensitivity analysis was applied, in which individual input parameters were perturbed while all remaining parameters were held constant. Sensitivity metrics were evaluated with respect to predefined pharmacokinetic outputs, such as exposure- and disposition-related endpoints.

For each selected parameter, sensitivity was quantified as the ratio of the relative change in the pharmacokinetic endpoint to the relative change in the perturbed model parameter. Accordingly, sensitivity coefficients were calculated as:

$$S_{j,i} = \frac{\Delta PK_j / PK_j}{\Delta p_i / p_i}$$

where $S_{j,i}$ denotes the sensitivity of pharmacokinetic endpoint PK_j to input parameter p_i, PK_j is the value obtained under baseline conditions, ΔPK_j is the change in the endpoint after perturbation of p_i, and $\Delta p_i$is

the applied change in parameter value. This formulation yields a dimensionless coefficient that reflects both the direction and magnitude of parameter influence on the selected endpoint.

Within the OSP implementation, sensitivities are estimated from simulations performed after parameter perturbation around the baseline value. For numerical stability, the reported local sensitivity can be obtained as an average across multiple small perturbations defined by the selected variation range and number of steps. This approach enables ranking of input parameters according to their relative influence on each pharmacokinetic endpoint and facilitates identification of parameters with the greatest impact on model behavior.

Supplementary Method S3. Statistical comparison of simulated dosing regimens

Tissue-specific target attainment rates were compared between simulated dosing regimens using two-sided paired Wilcoxon signed-rank tests, with the same tissues matched across regimens. For comparisons involving three regimens, including the three dose levels and three dosing frequencies, pairwise p values were adjusted using the Bonferroni method. Comparisons between two regimens, including 50 IU/kg twice weekly versus 100 IU/kg once weekly and administration of the second dose at 72 versus 96 h, were performed without multiplicity adjustment. A two-sided adjusted p value < 0.05 was considered statistically significant. Statistical analyses were performed using R version 4.4.3.

Supplementary Figures

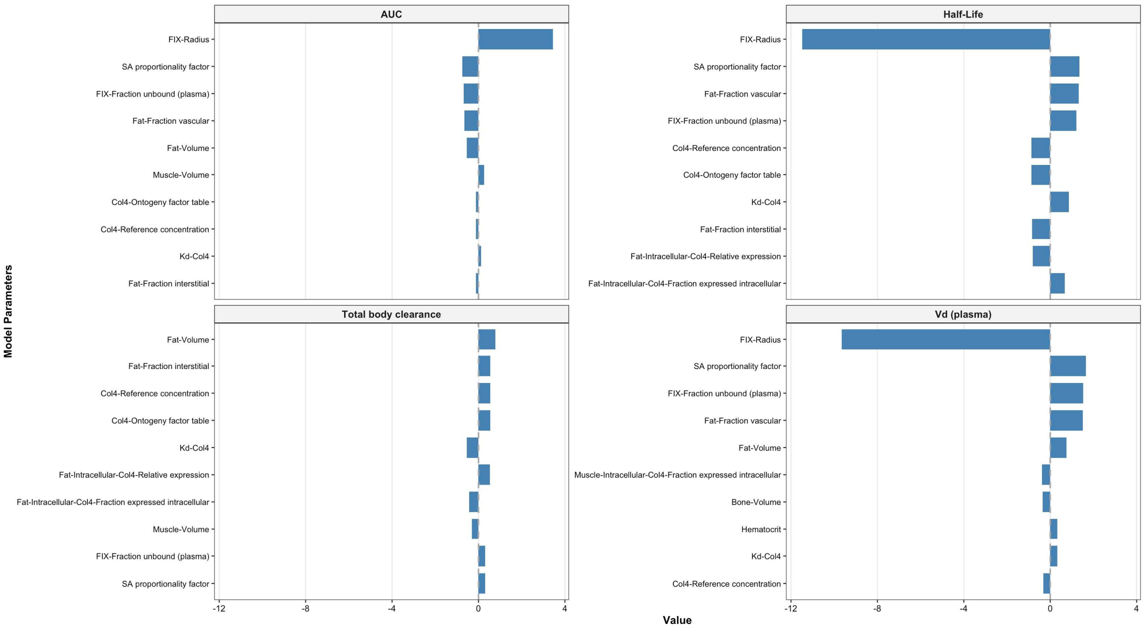

Figure S1. Sensitivity analysis of the area under the curve, half-life, total clearance, and plasma volume of distribution of the FIX-CTBB PBPK model. Sensitivity values are shown. AUC, area under the curve; V_d, volume of distribution Col4, type IV collagen; Kd, dissociation constant; VEBP, vascular endothelial binding partner; SA, surface area.

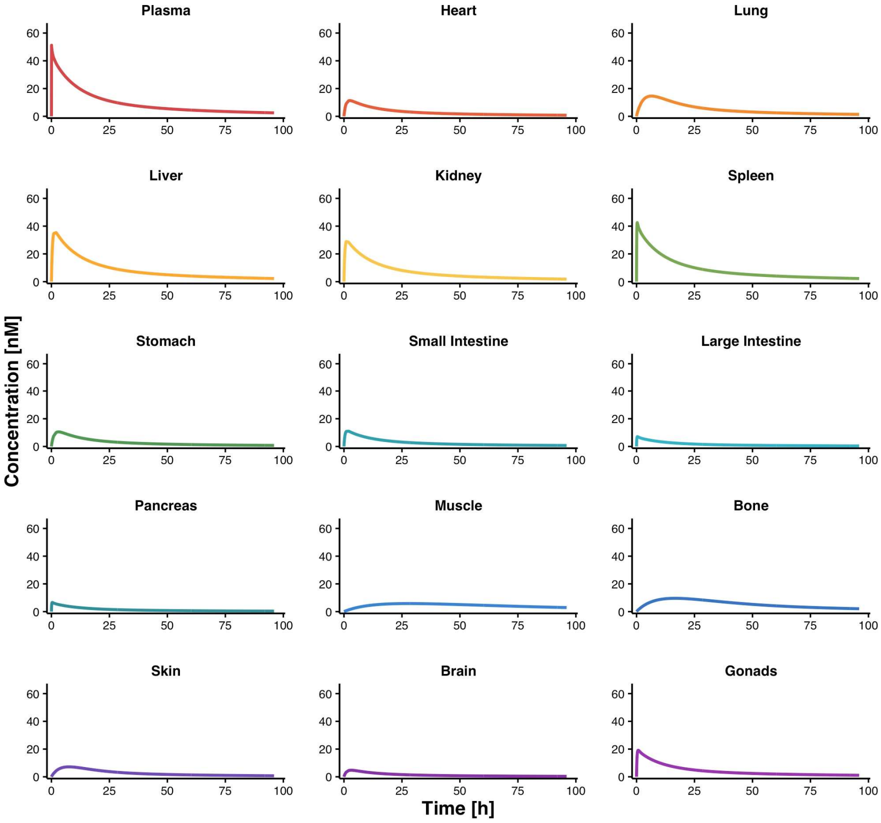

Figure S2. The distribution of FIX in various tissues extravascular space in children. The y-axis represents FIX concentration (nM), and the x-axis represents time (t). The figure shows FIX concentration-time profiles across tissues, illustrating the extravascular distribution of FIX in children.

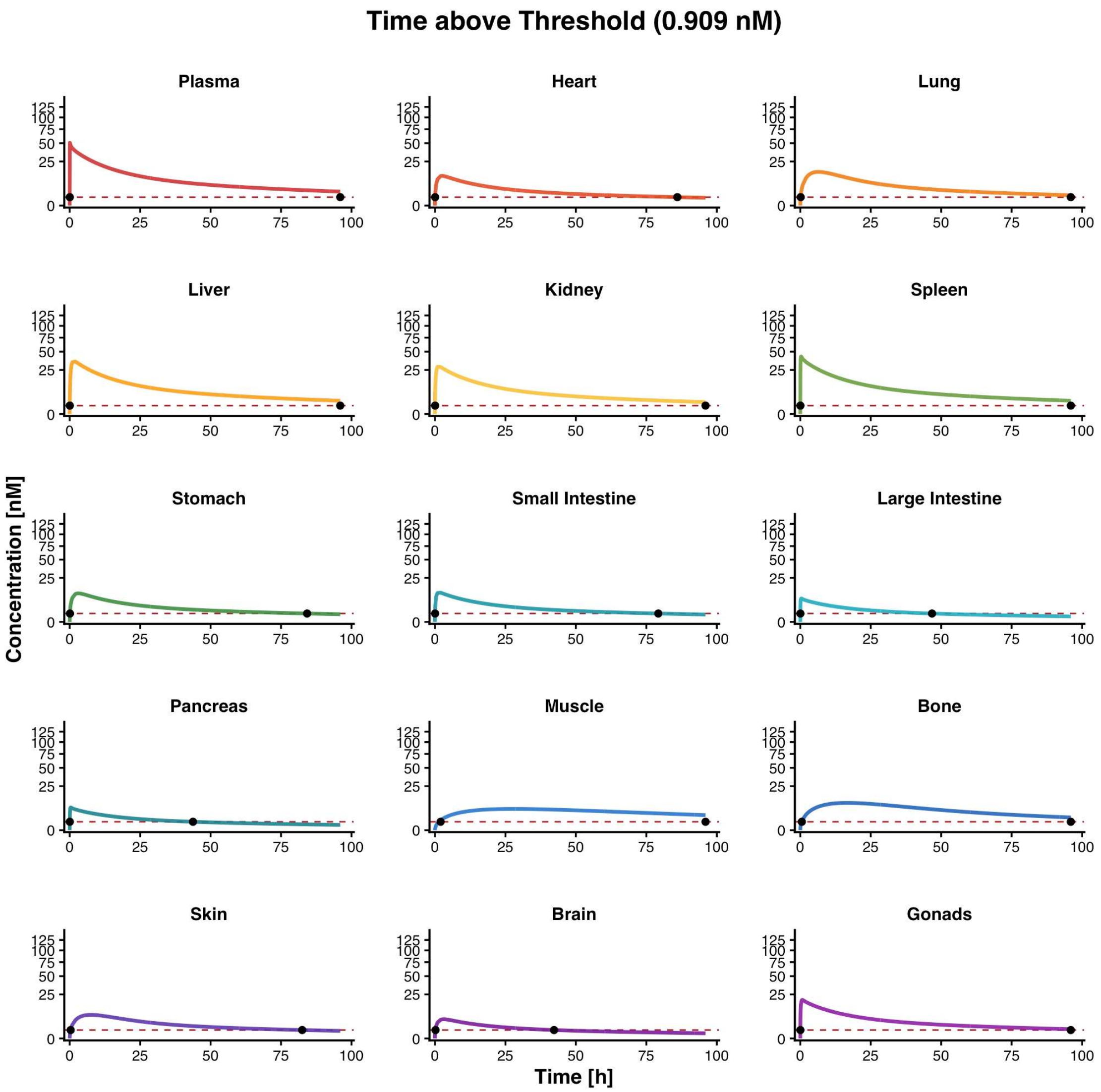

Figure S3. Time intervals during which FIX concentrations exceeded 1% in different tissues after administration of 50 IU/kg. The y-axis represents FIX concentration (nM), and the x-axis represents time (t). The red line indicates the 1% concentration threshold. Black dots mark the start and end times at which FIX concentrations reached the 1% target.

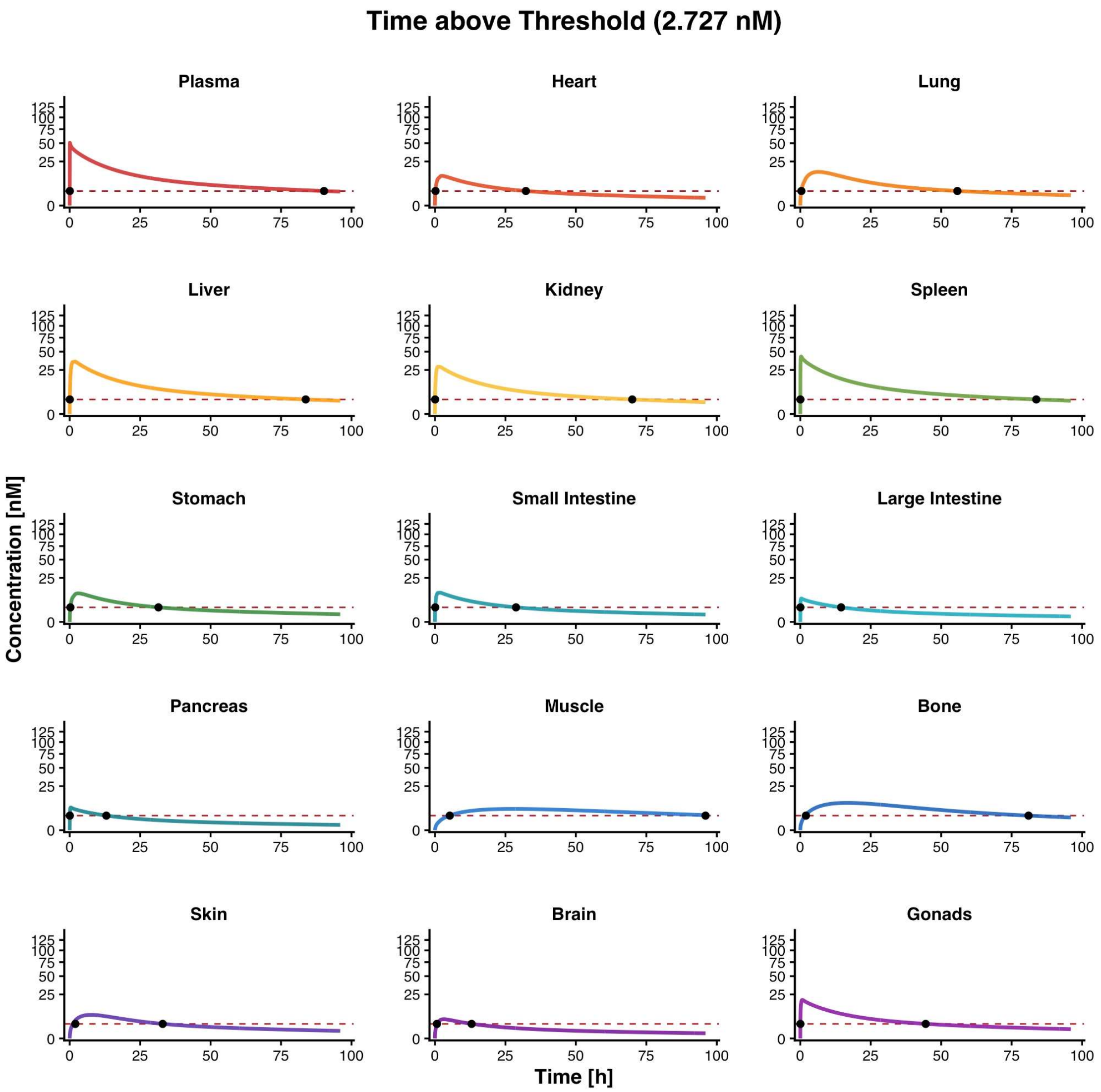

Figure S4. Time intervals during which FIX concentrations exceeded 3% in different tissues after administration of 50 IU/kg. The y-axis represents FIX concentration (nM), and the x-axis represents time (t). The red line indicates the 3% concentration threshold. Black dots mark the start and end times at which FIX concentrations reached the 3% target.

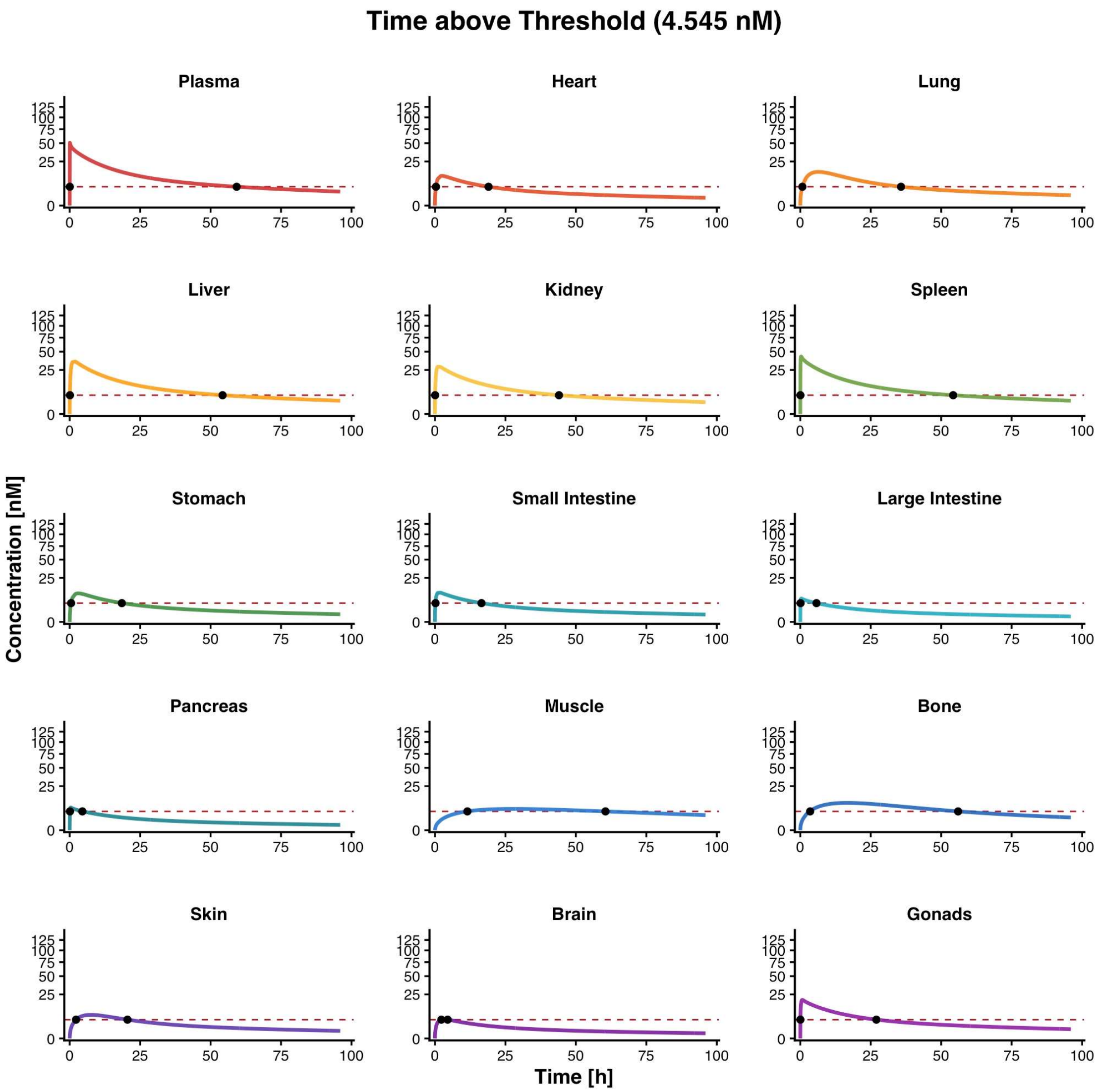

Figure S5. Time intervals during which FIX concentrations exceeded 5% in different tissues after administration of 50 IU/kg. The y-axis represents FIX concentration (nM), and the x-axis represents time (t). The red line indicates the 5% concentration threshold. Black dots mark the start and end times at which FIX concentrations reached the 5% target.

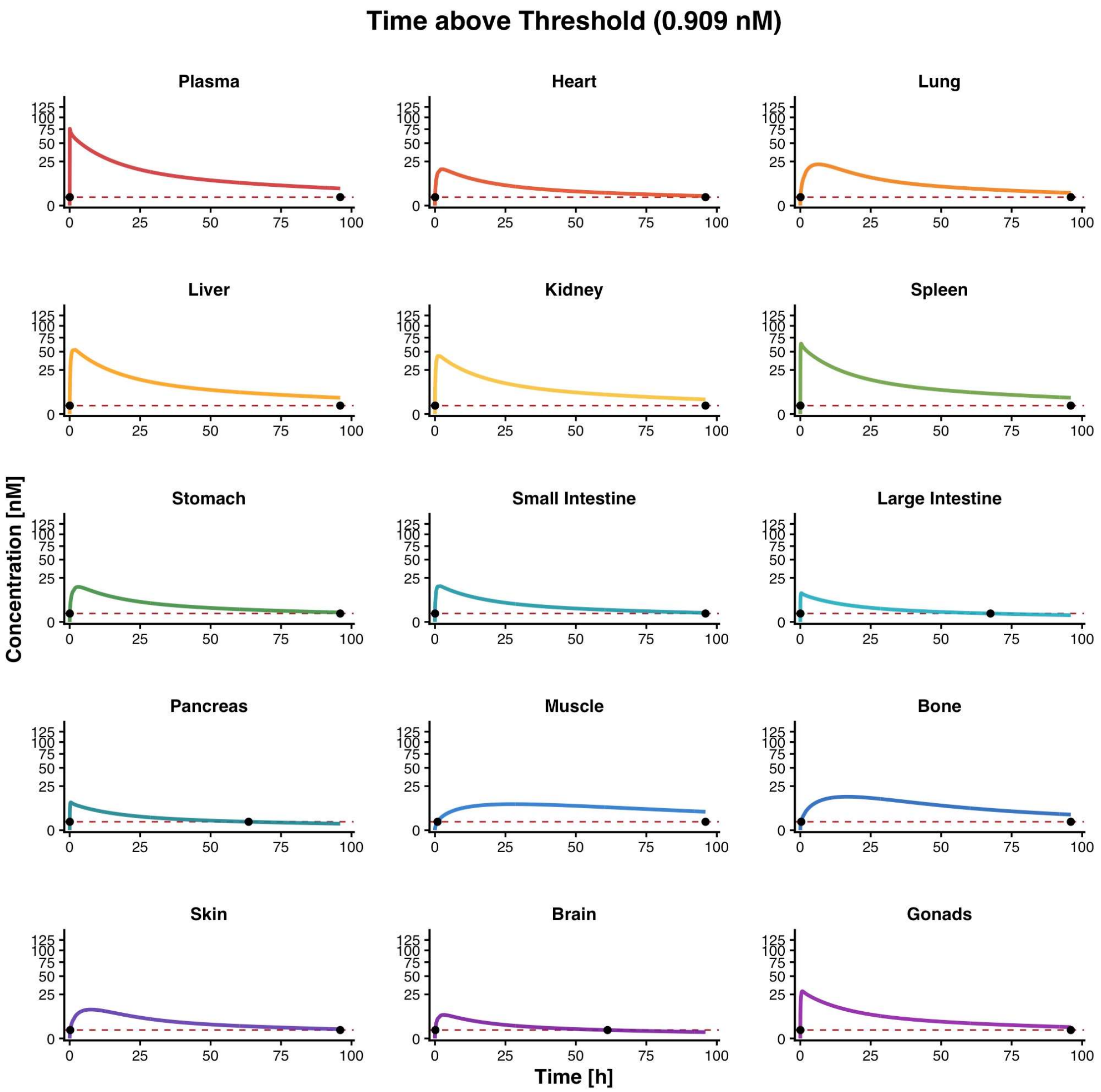

Figure S6. Time intervals during which FIX concentrations exceeded 1% in different tissues after administration of 75 IU/kg. The y-axis represents FIX concentration (nM), and the x-axis represents time (t). The red line indicates the 1% concentration threshold. Black dots mark the start and end times at which FIX concentrations reached the 1% target.

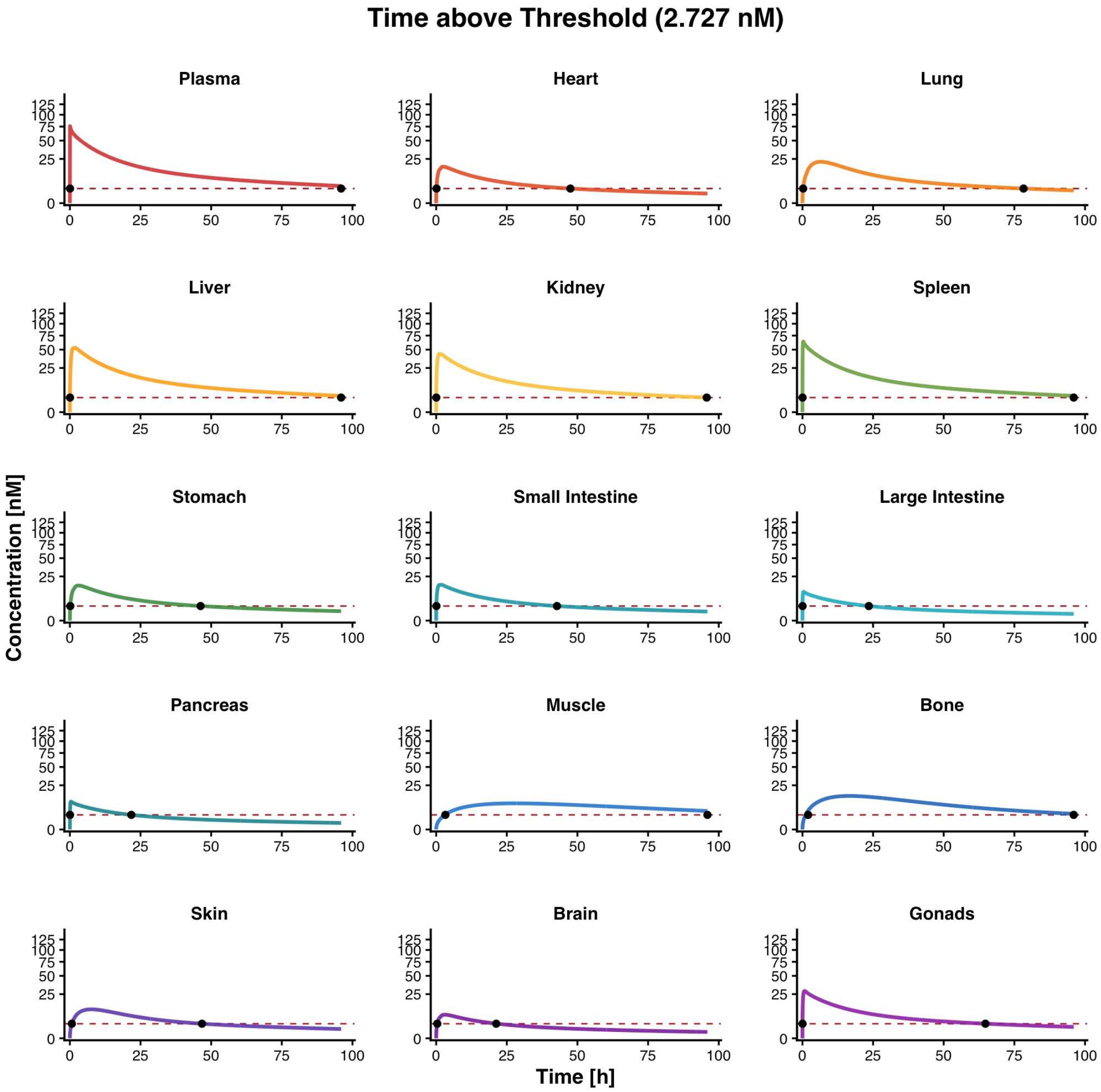

Figure S7. Time intervals during which FIX concentrations exceeded 3% in different tissues after administration of 75 IU/kg. The y-axis represents FIX concentration (nM), and the x-axis represents time (t). The red line indicates the 3% concentration threshold. Black dots mark the start and end times at which FIX concentrations reached the 3% target.

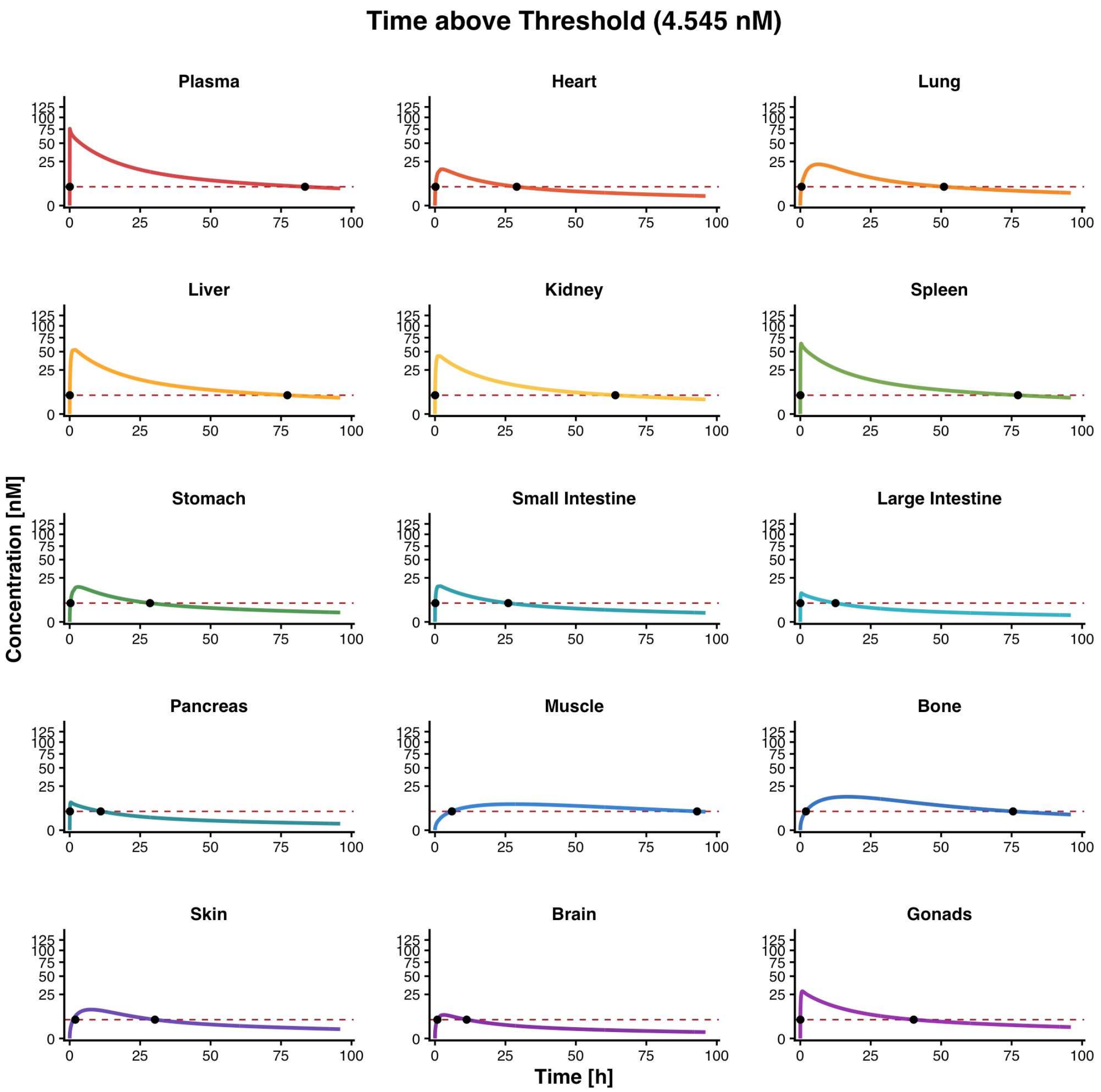

Figure S8. Time intervals during which FIX concentrations exceeded 5% in different tissues after administration of 75 IU/kg. The y-axis represents FIX concentration (nM), and the x-axis represents time (t). The red line indicates the 5% concentration threshold. Black dots mark the start and end times at which FIX concentrations reached the 5% target.

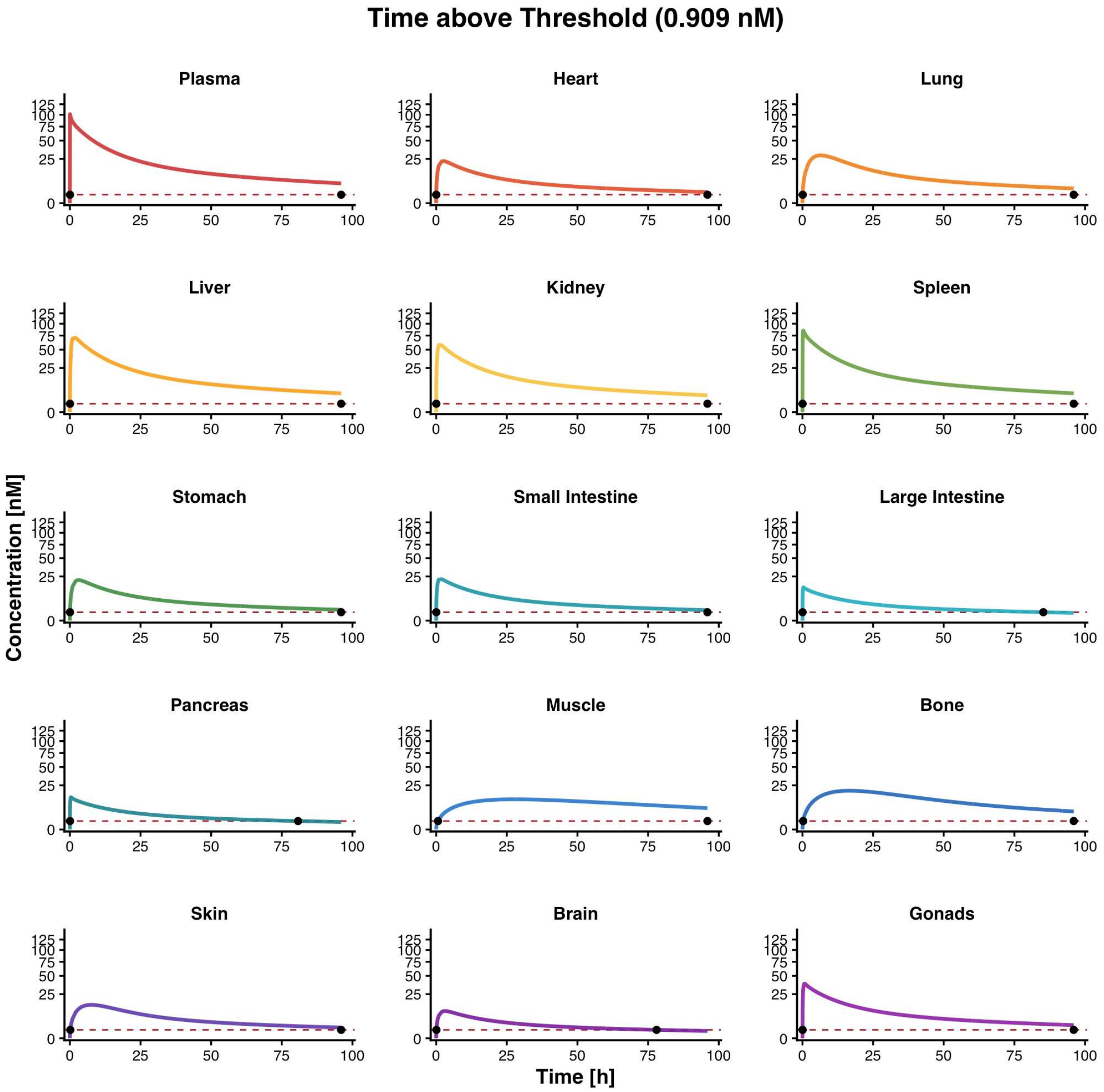

Figure S9. Time intervals during which FIX concentrations exceeded 1% in different tissues after administration of 100 IU/kg. The y-axis represents FIX concentration (nM), and the x-axis represents time (t). The red line indicates the 1% concentration threshold. Black dots mark the start and end times at which FIX concentrations reached the 1% target.

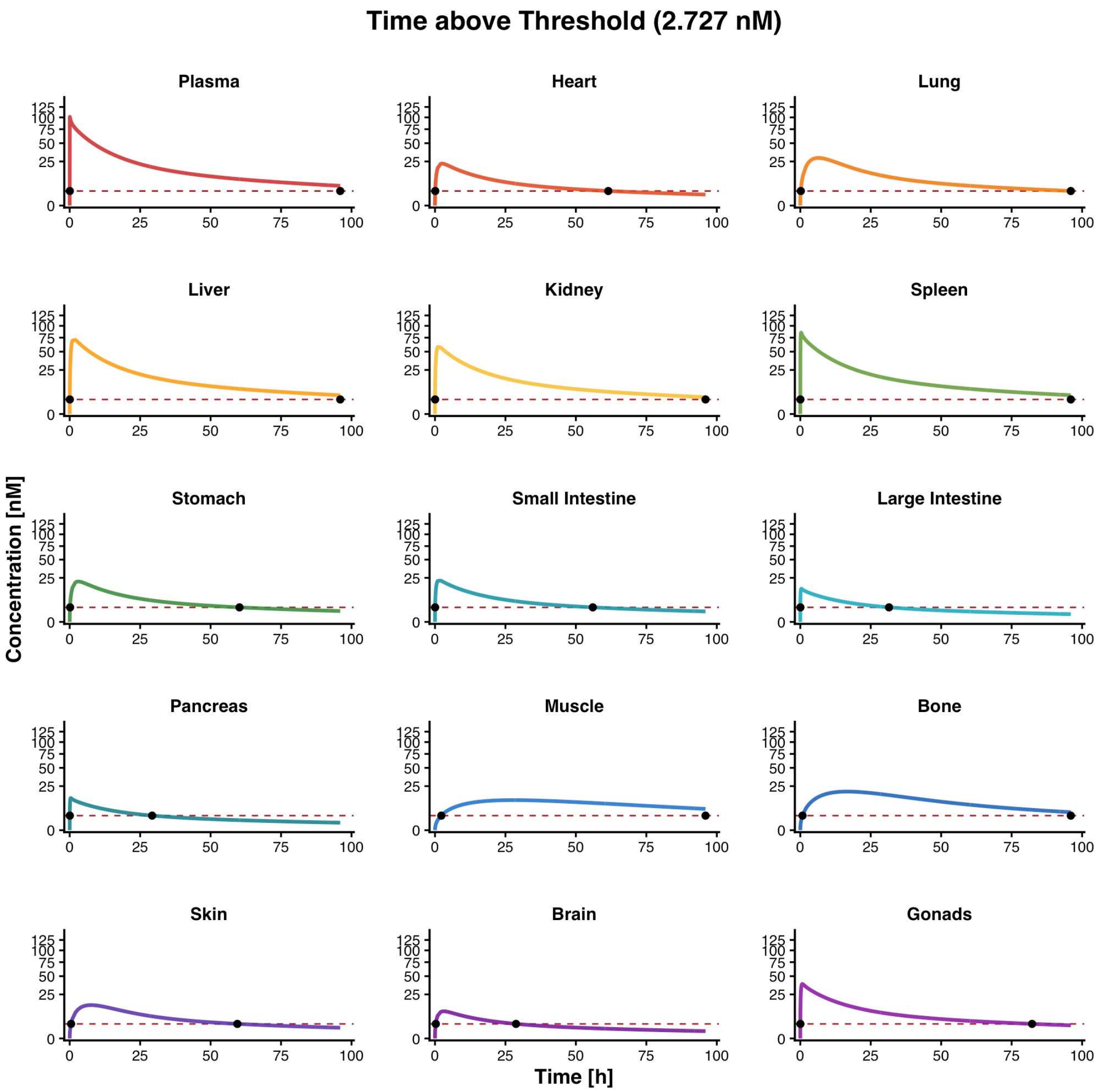

Figure S10. Time intervals during which FIX concentrations exceeded 3% in different tissues after administration of 100 IU/kg. The y-axis represents FIX concentration (nM), and the x-axis represents time (t). The red line indicates the 3% concentration threshold. Black dots mark the start and end times at which FIX concentrations reached the 3% target.

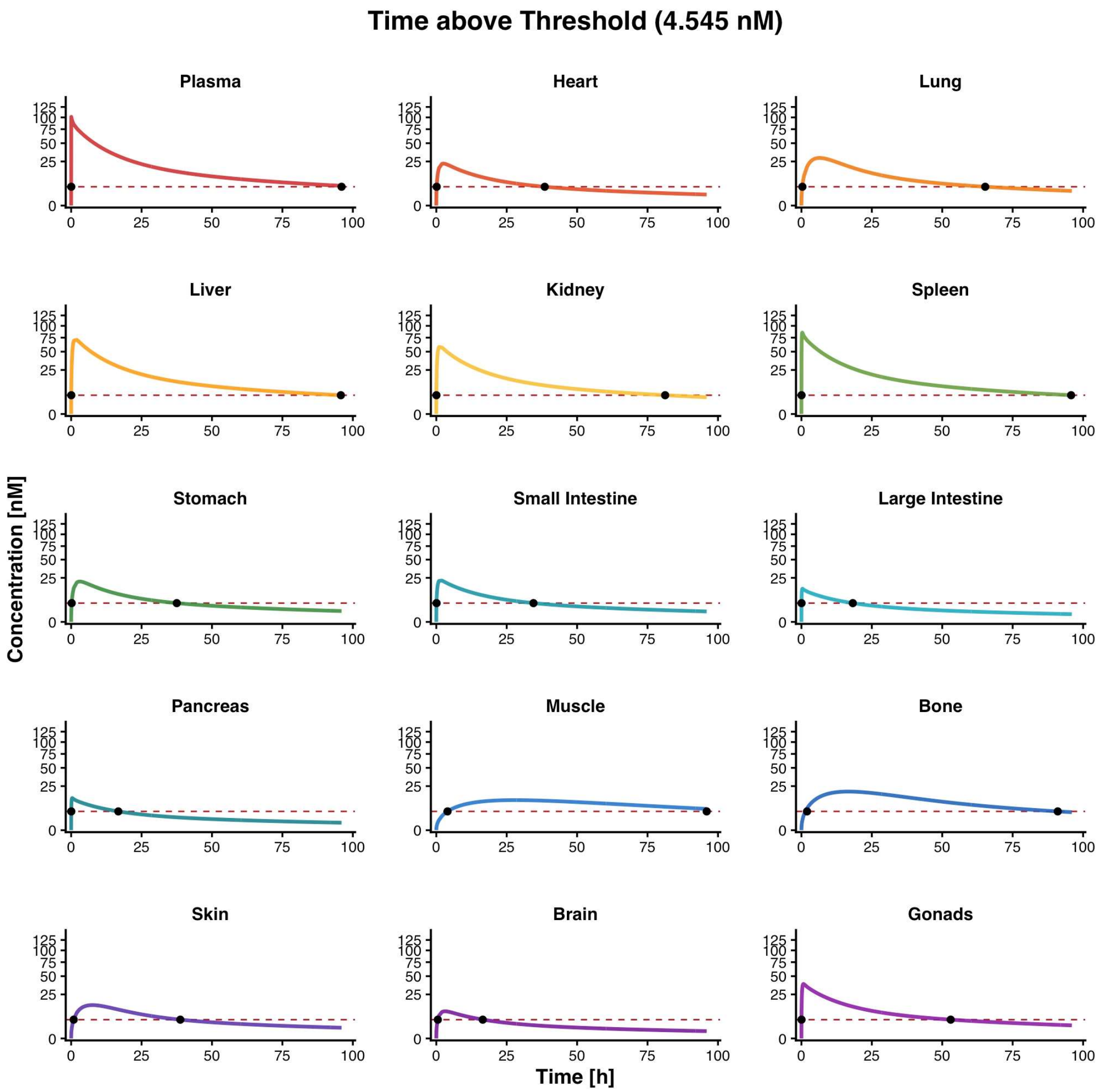

Figure S11. Time intervals during which FIX concentrations exceeded 5% in different tissues after administration of 100 IU/kg. The y-axis represents FIX concentration (nM), and the x-axis represents time (t). The red line indicates the 5% concentration threshold. Black dots mark the start and end times at which FIX concentrations reached the 5% target.

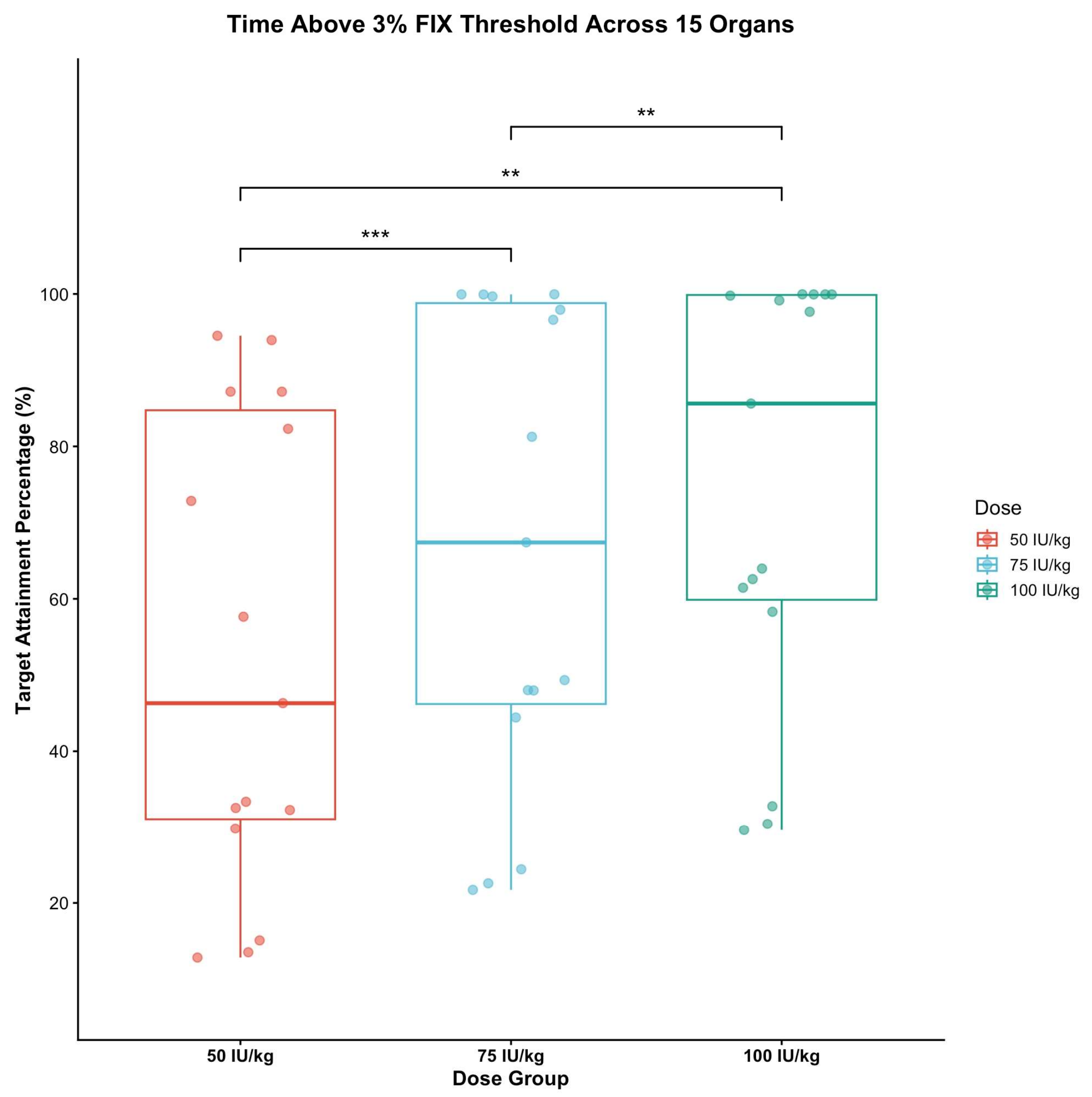

Figure S12. Box plots of target attainment rates (>3%) at different dose levels. This figure summarizes differences in the rate of achieving FIX concentrations above 3% across tissues among the different dose groups. Red represents the 50 IU/kg group, blue represents the 75 IU/kg group, and green represents the 100 IU/kg group.

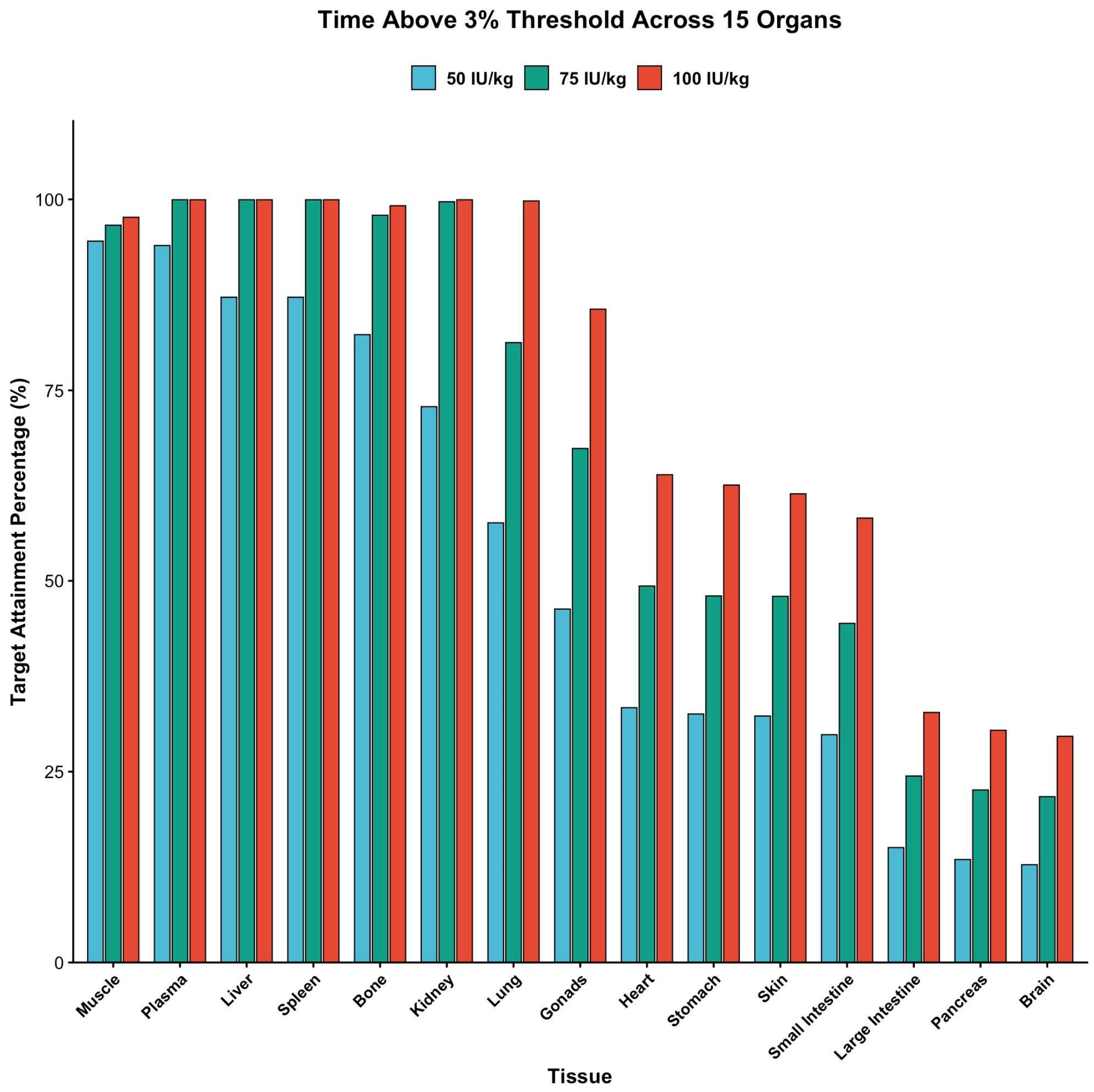

Figure S13. Bar plots of target attainment rates (>3%) at different dose levels. This figure shows the differences in the rate of achieving FIX concentrations above 3% across tissues under different dosing regimens. Blue represents 50 IU/kg, green represents 75 IU/kg, and red represents 100 IU/kg.

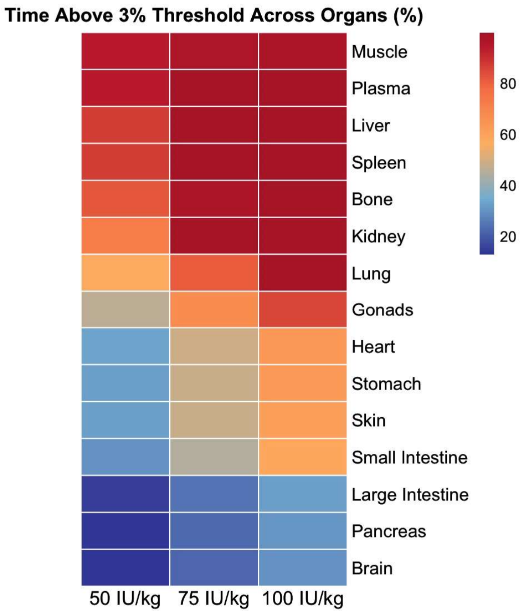

Figure S14. Heatmap of target attainment rates (>3%) at different dose levels. This figure shows the differences in the rate of achieving FIX concentrations above 3% across tissues under different dosing regimens. Colors range from blue to red, indicating increasing target attainment. From left to right, the panels represent the 50 IU/kg, 75 IU/kg, and 100 IU/kg dose groups.

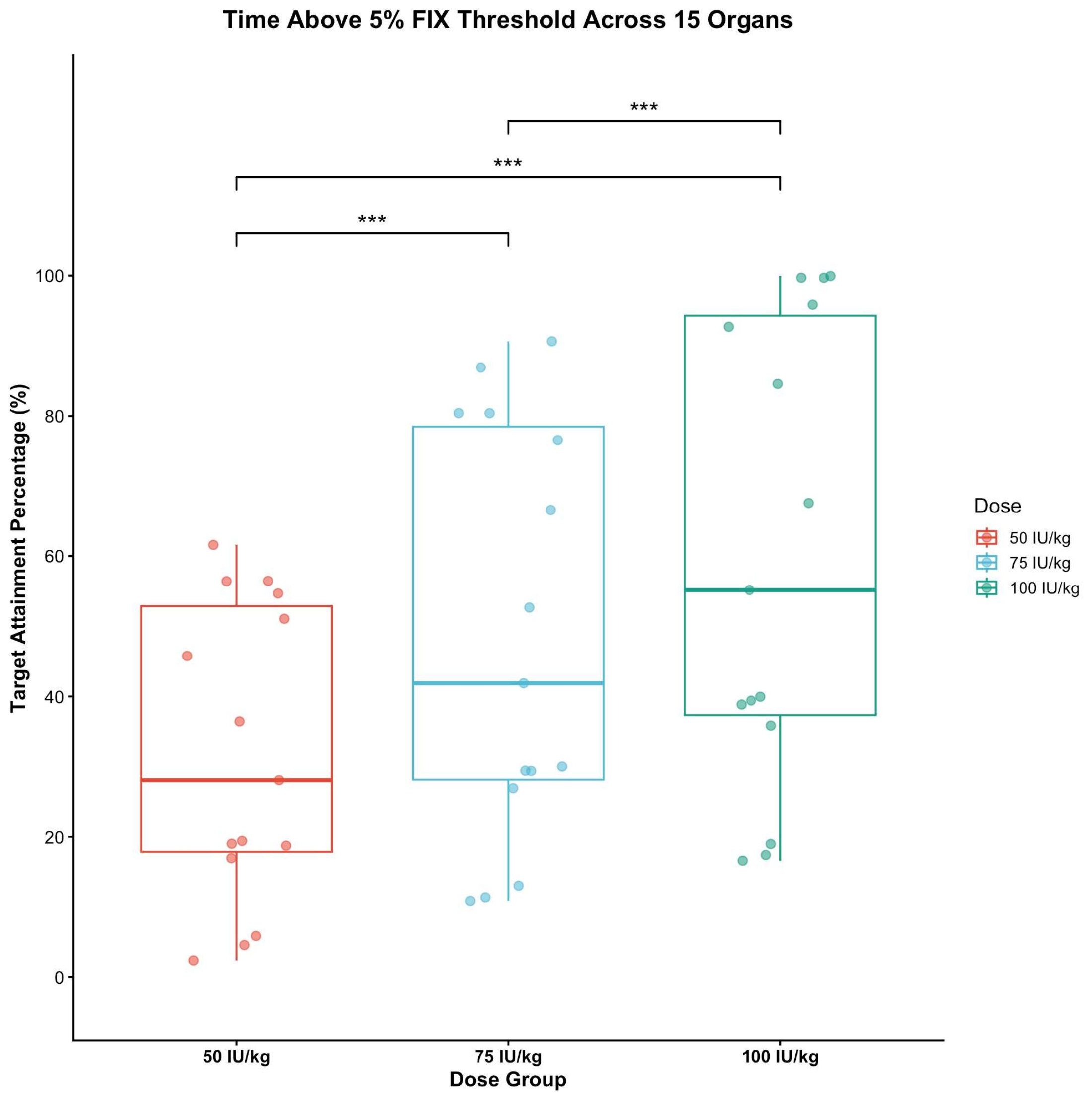

Figure S15. Box plots of target attainment rates (>5%) at different dose levels. This figure summarizes differences in the rate of achieving FIX concentrations above 5% across tissues among the different dose groups. Red represents the 50 IU/kg group, blue represents the 75 IU/kg group, and green represents the 100 IU/kg group.

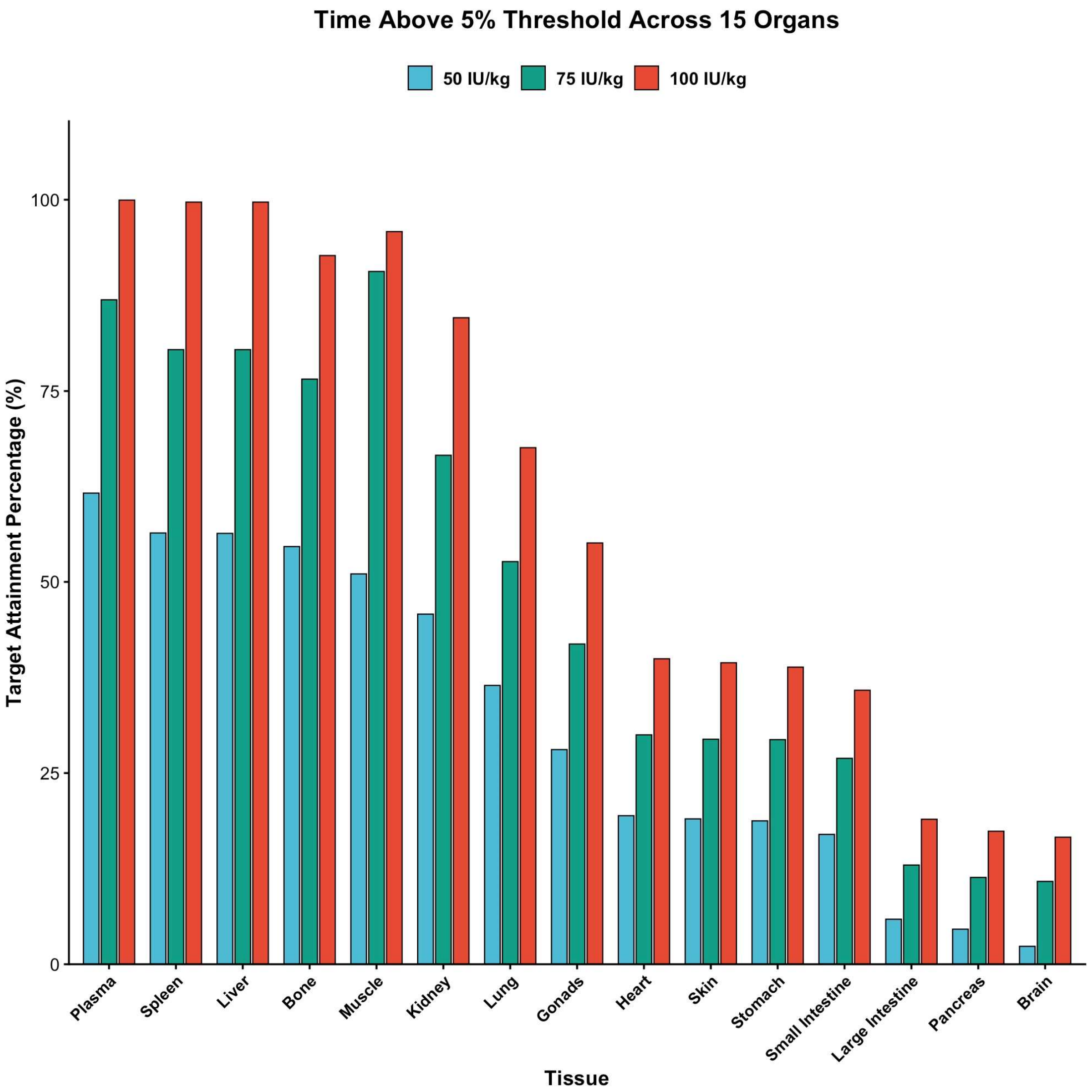

Figure S16. Bar plots of target attainment rates (>5%) at different dose levels. This figure shows the differences in the rate of achieving FIX concentrations above 5% across tissues under different dosing regimens. Blue represents 50 IU/kg, green represents 75 IU/kg, and red represents 100 IU/kg.

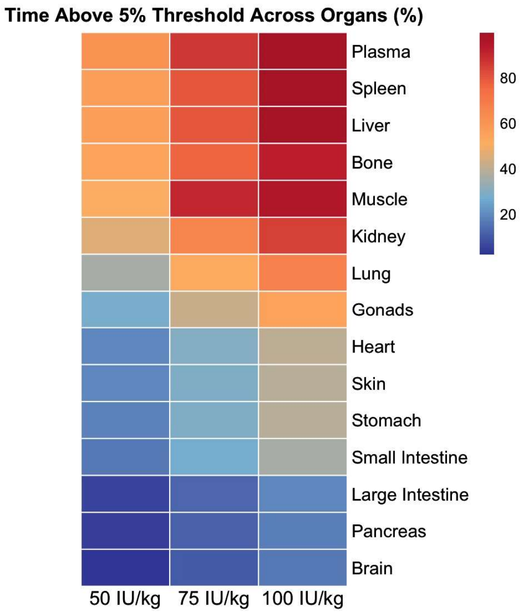

Figure S17. Heatmap of target attainment rates (>5%) at different dose levels. This figure shows the differences in the rate of achieving FIX concentrations above 5% across tissues under different dosing regimens. Colors range from blue to red, indicating increasing target attainment. From left to right, the panels represent the 50 IU/kg, 75 IU/kg, and 100 IU/kg dose groups.

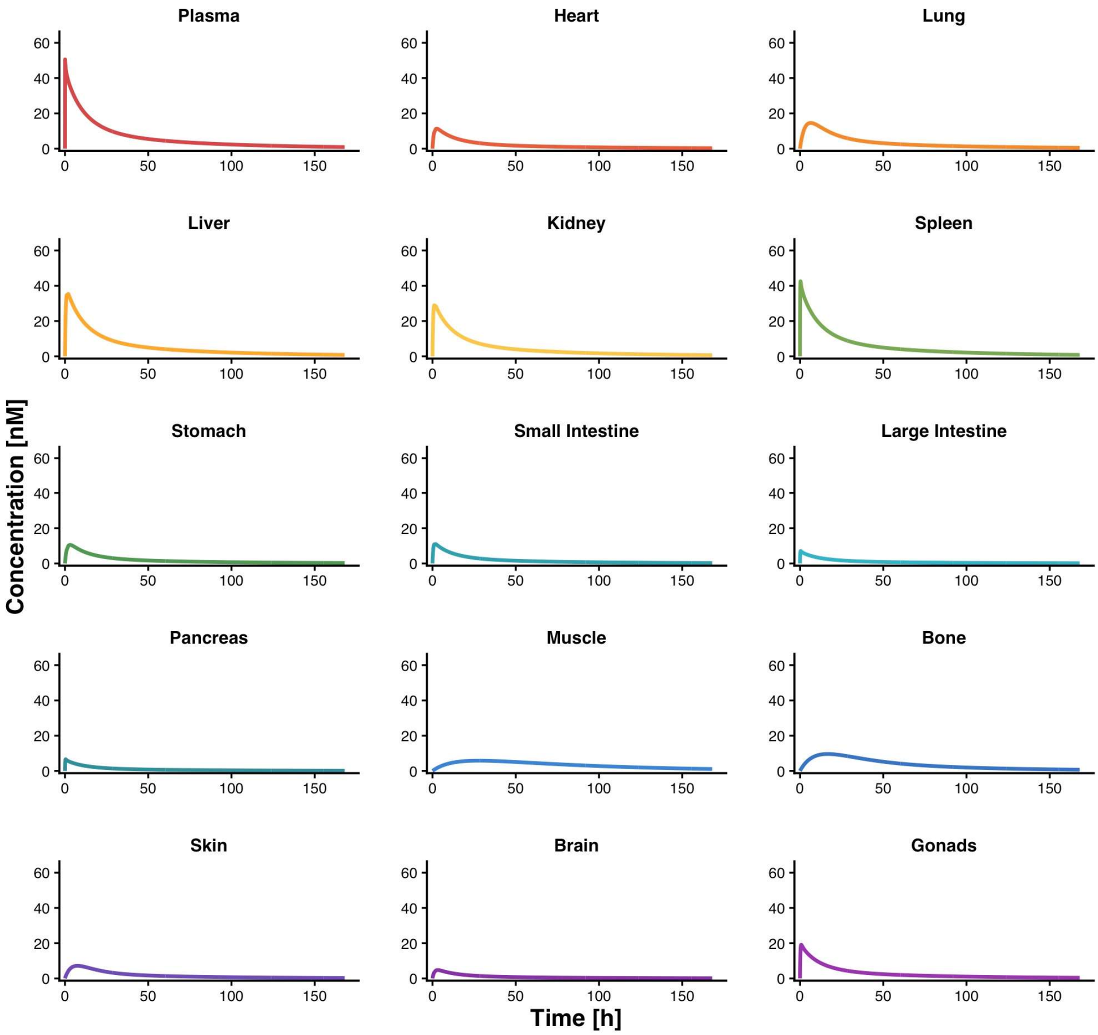

Figure S18. FIX distribution profiles under 50 IU/kg once-weekly dosing. The y-axis represents FIX concentration (nM), and the x-axis represents time (t). This figure shows the distribution of FIX across tissues under 50 IU/kg once-weekly dosing regimen.

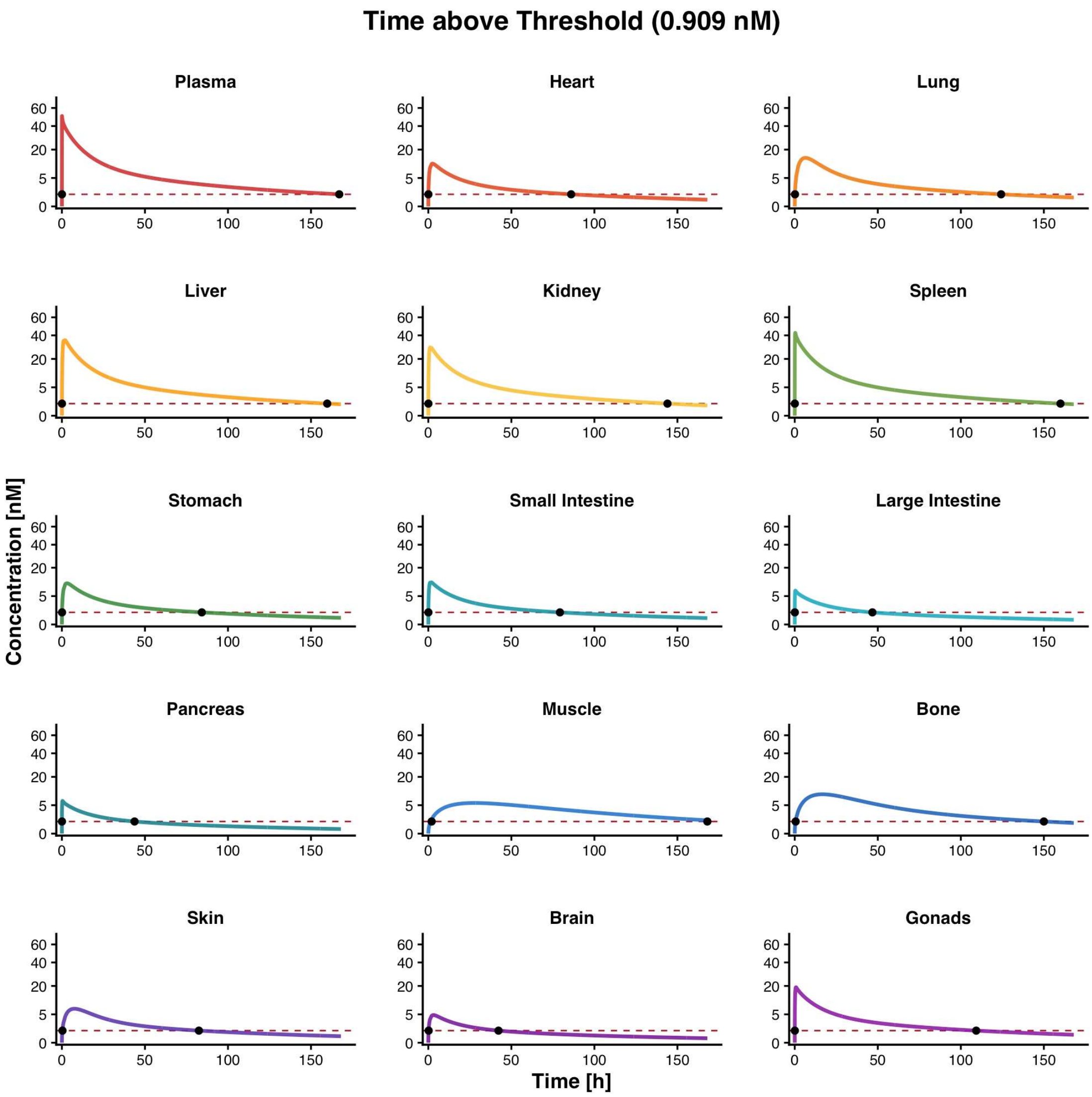

Figure S19. Time intervals during which FIX concentrations exceeded 1% in different tissues after once-weekly dosing at 50 IU/kg. The y-axis represents FIX concentration (nM), and the x-axis represents time (t). The red line indicates the 1% concentration threshold. Black dots mark the start and end times at which FIX concentrations reached the 1% target.

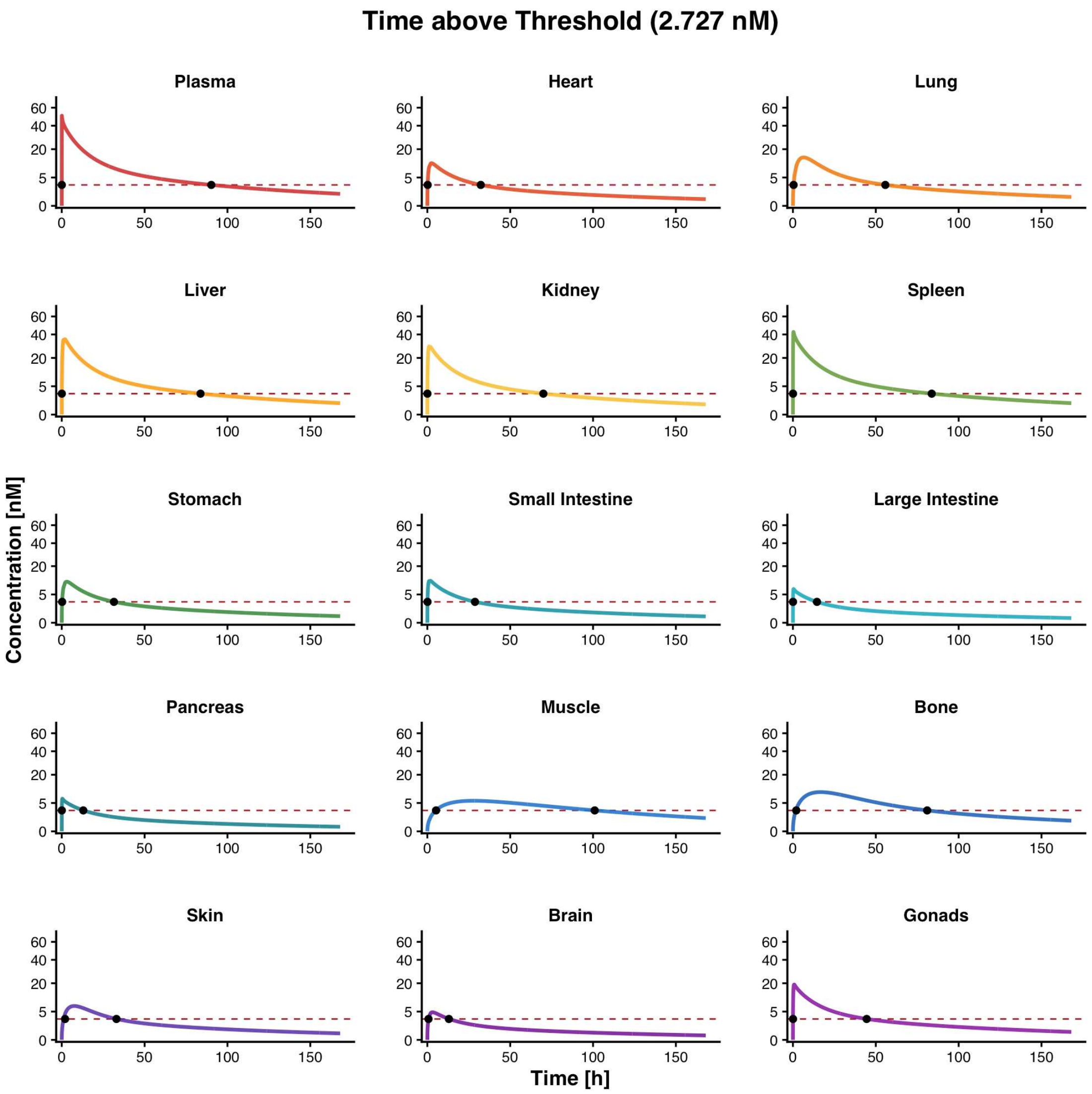

Figure S20. Time intervals during which FIX concentrations exceeded 3% in different tissues after once-weekly dosing at 50 IU/kg. The y-axis represents FIX concentration (nM), and the x-axis represents time (t). The red line indicates the 3% concentration threshold. Black dots mark the start and end times at which FIX concentrations reached the 3% target.

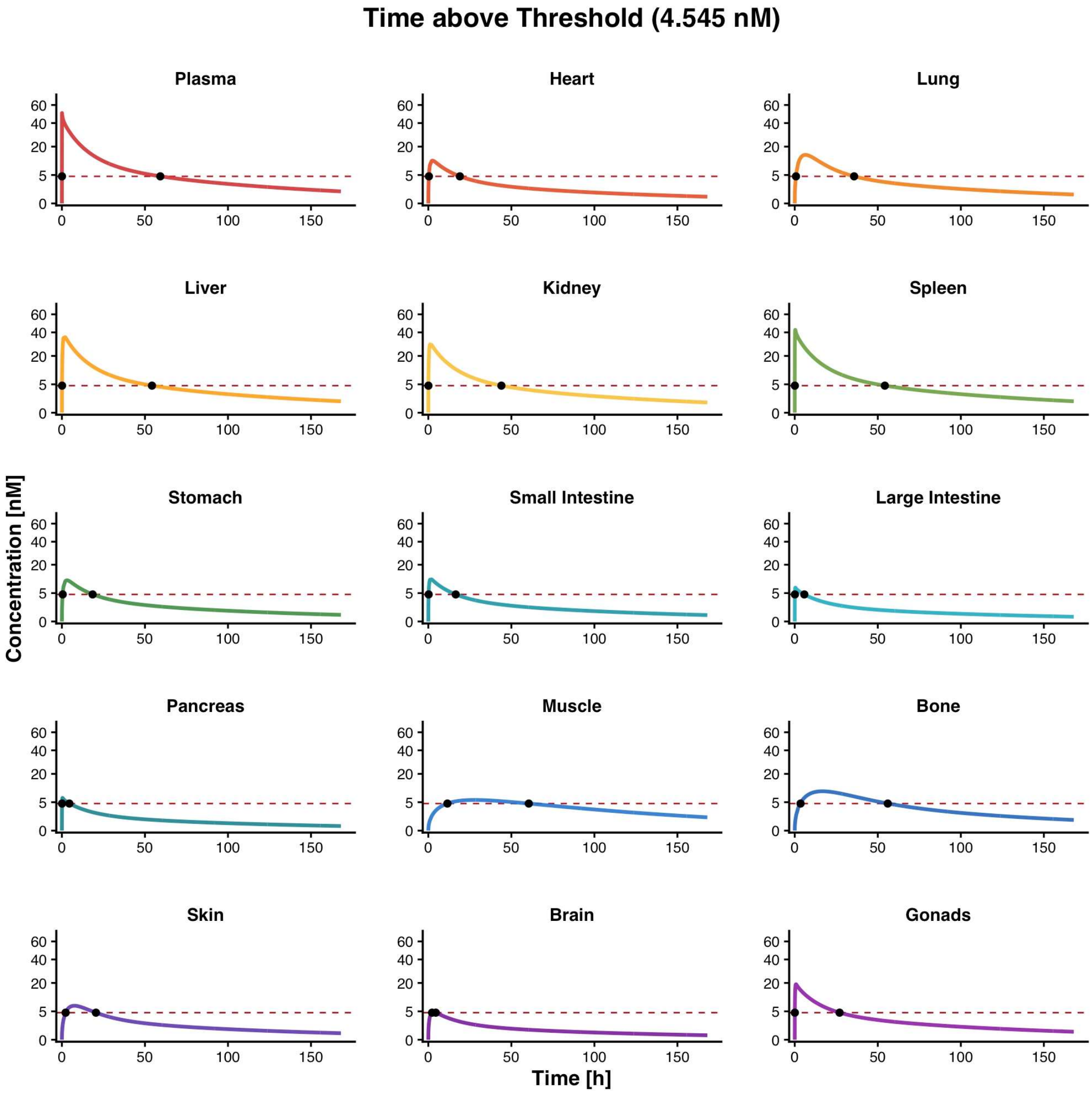

Figure S21. Time intervals during which FIX concentrations exceeded 5% in different tissues after once-weekly dosing at 50 IU/kg. The y-axis represents FIX concentration (nM), and the x-axis represents time (t). The red line indicates the 5% concentration threshold. Black dots mark the start and end times at which FIX concentrations reached the 5% target.

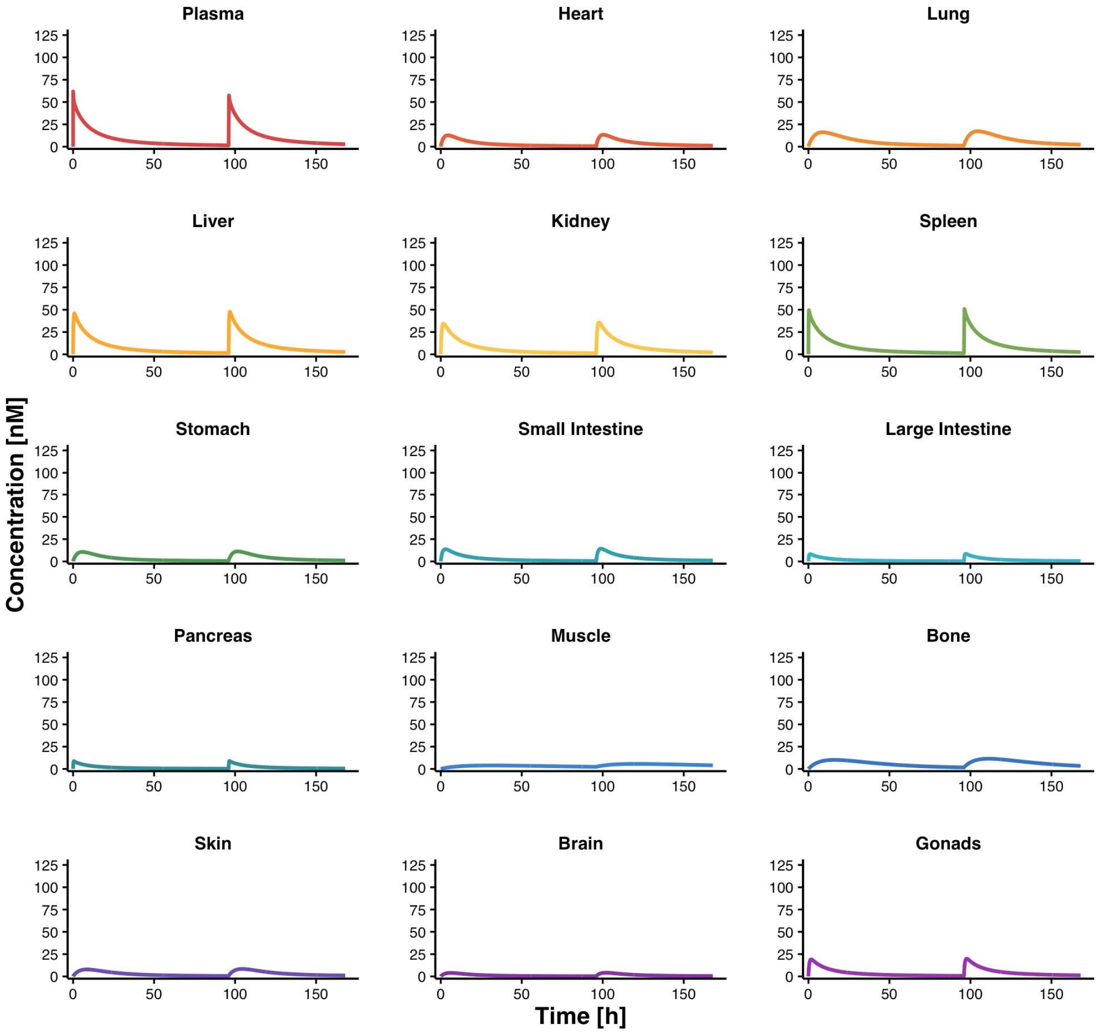

Figure S22. FIX distribution profiles under 50 IU/kg twice-weekly dosing. The y-axis represents FIX concentration (nM), and the x-axis represents time (t). This figure shows the distribution of FIX across tissues under 50 IU/kg twice-weekly dosing regimen.

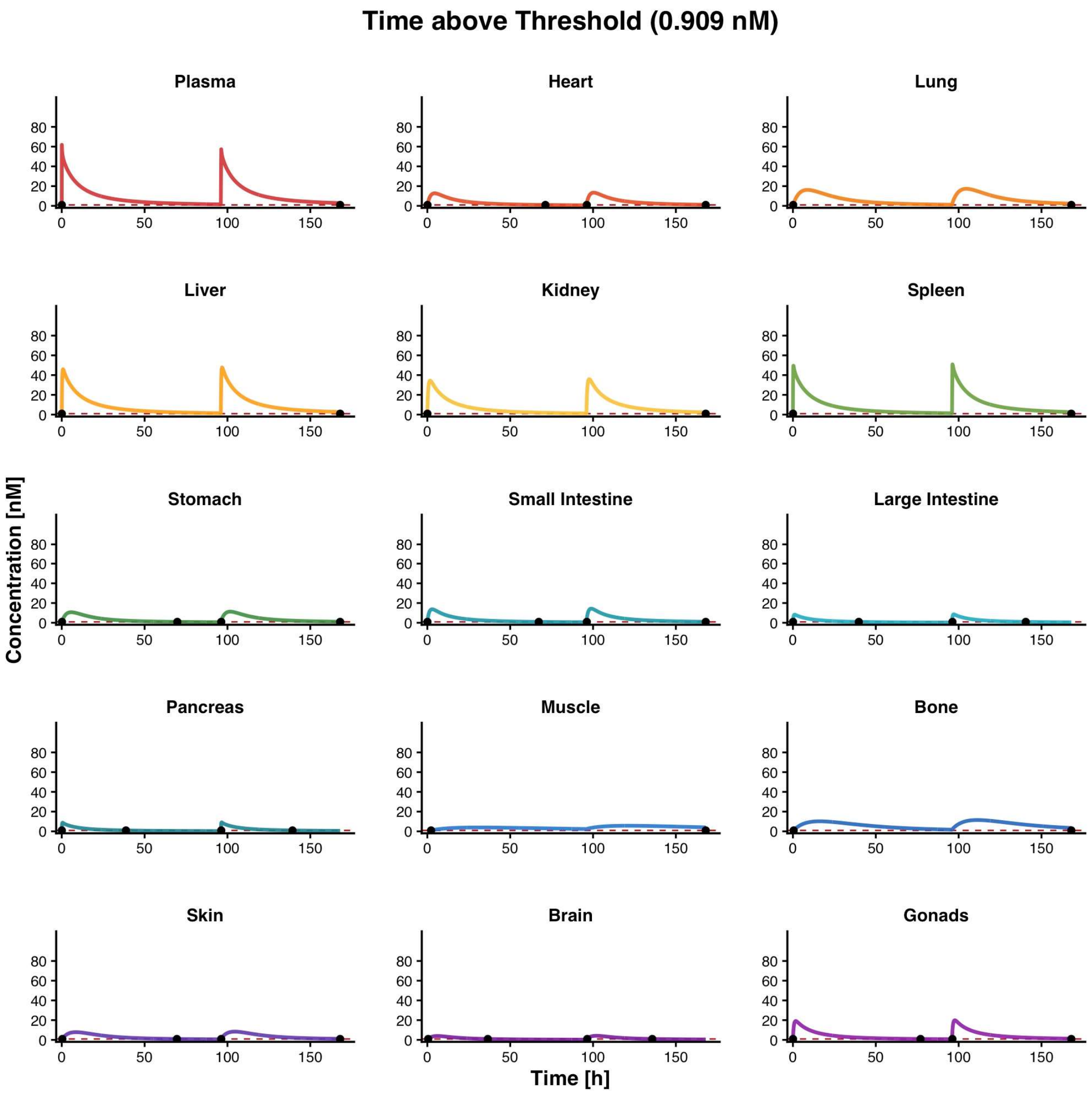

Figure S23. Time intervals during which FIX concentrations exceeded 1% in different tissues after twice-weekly dosing at 50 IU/kg. The y-axis represents FIX concentration (nM), and the x-axis represents time (t). The red line indicates the 1% concentration threshold. Black dots mark the start and end times at which FIX concentrations reached the 1% target.

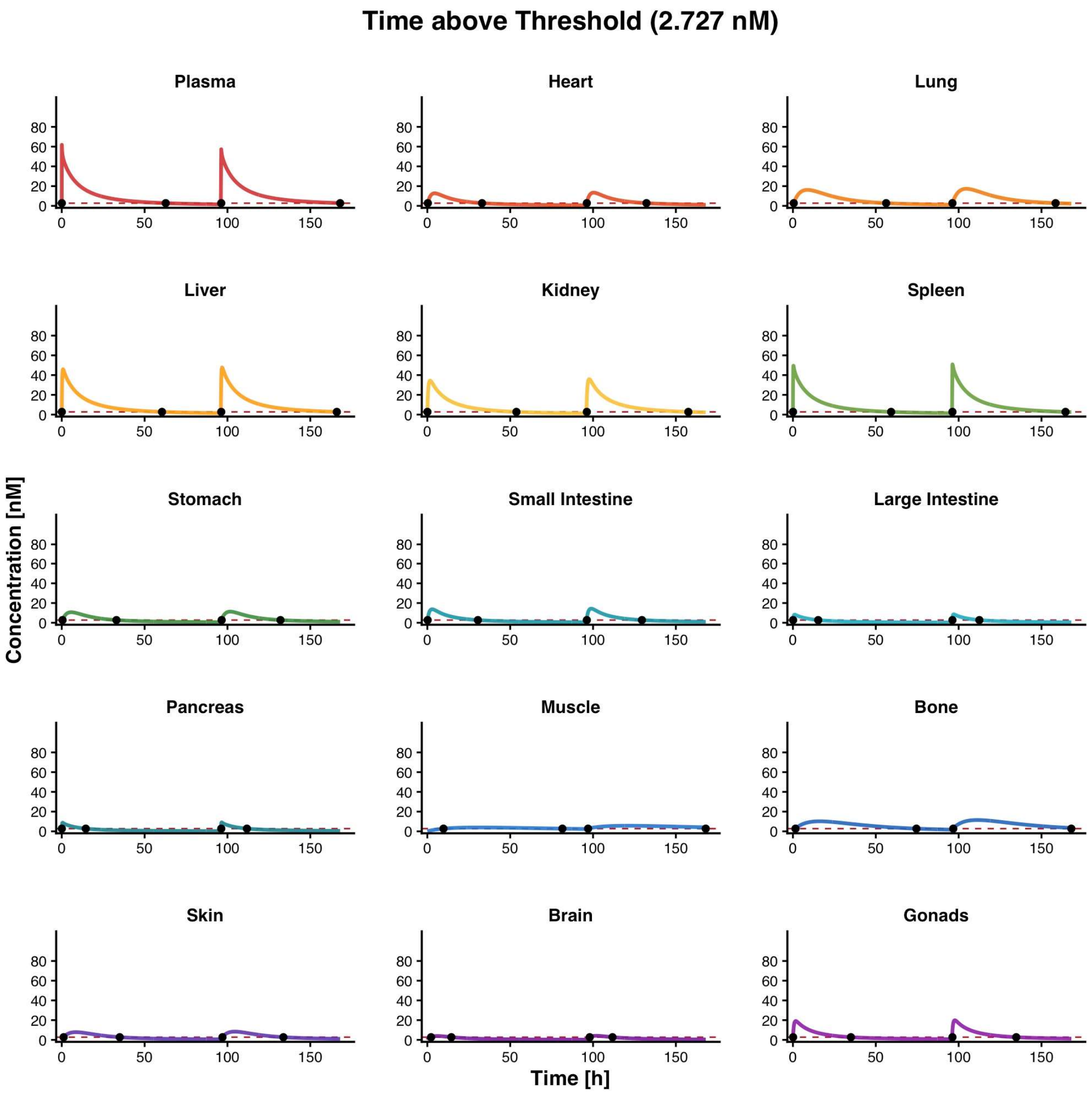

Figure S24. Time intervals during which FIX concentrations exceeded 3% in different tissues after twice-weekly dosing at 50 IU/kg. The y-axis represents FIX concentration (nM), and the x-axis represents time (t). The red line indicates the 3% concentration threshold. Black dots mark the start and end times at which FIX concentrations reached the 3% target.

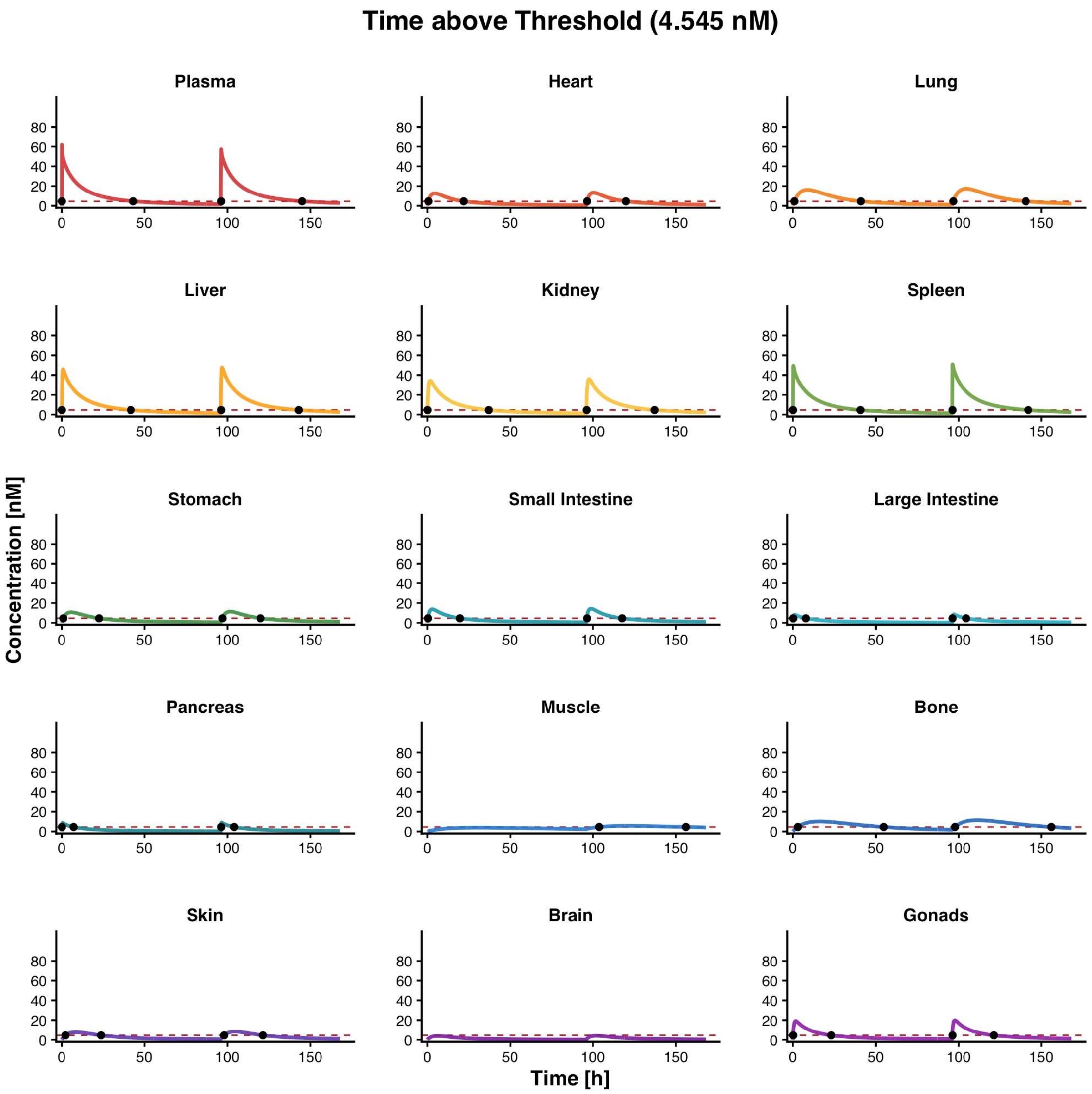

Figure S25. Time intervals during which FIX concentrations exceeded 5% in different tissues after twice-weekly dosing at 50 IU/kg. The y-axis represents FIX concentration (nM), and the x-axis represents time (t). The red line indicates the 5% concentration threshold. Black dots mark the start and end times at which FIX concentrations reached the 5% target.

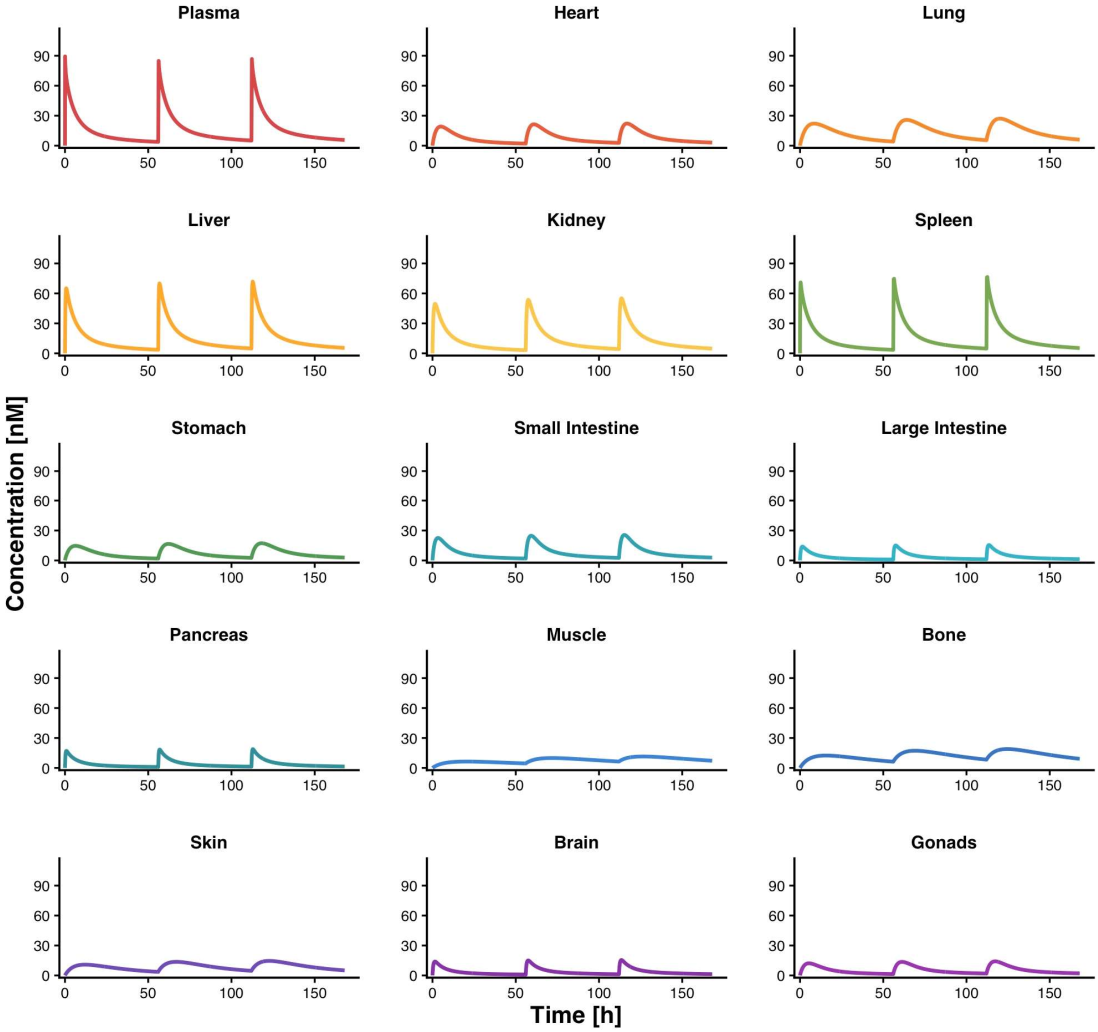

Figure S26. FIX distribution profiles under 50 IU/kg three times-weekly dosing. The y-axis represents FIX concentration (nM), and the x-axis represents time (t). This figure shows the distribution of FIX across tissues under 50 IU/kg three times-weekly dosing regimen.

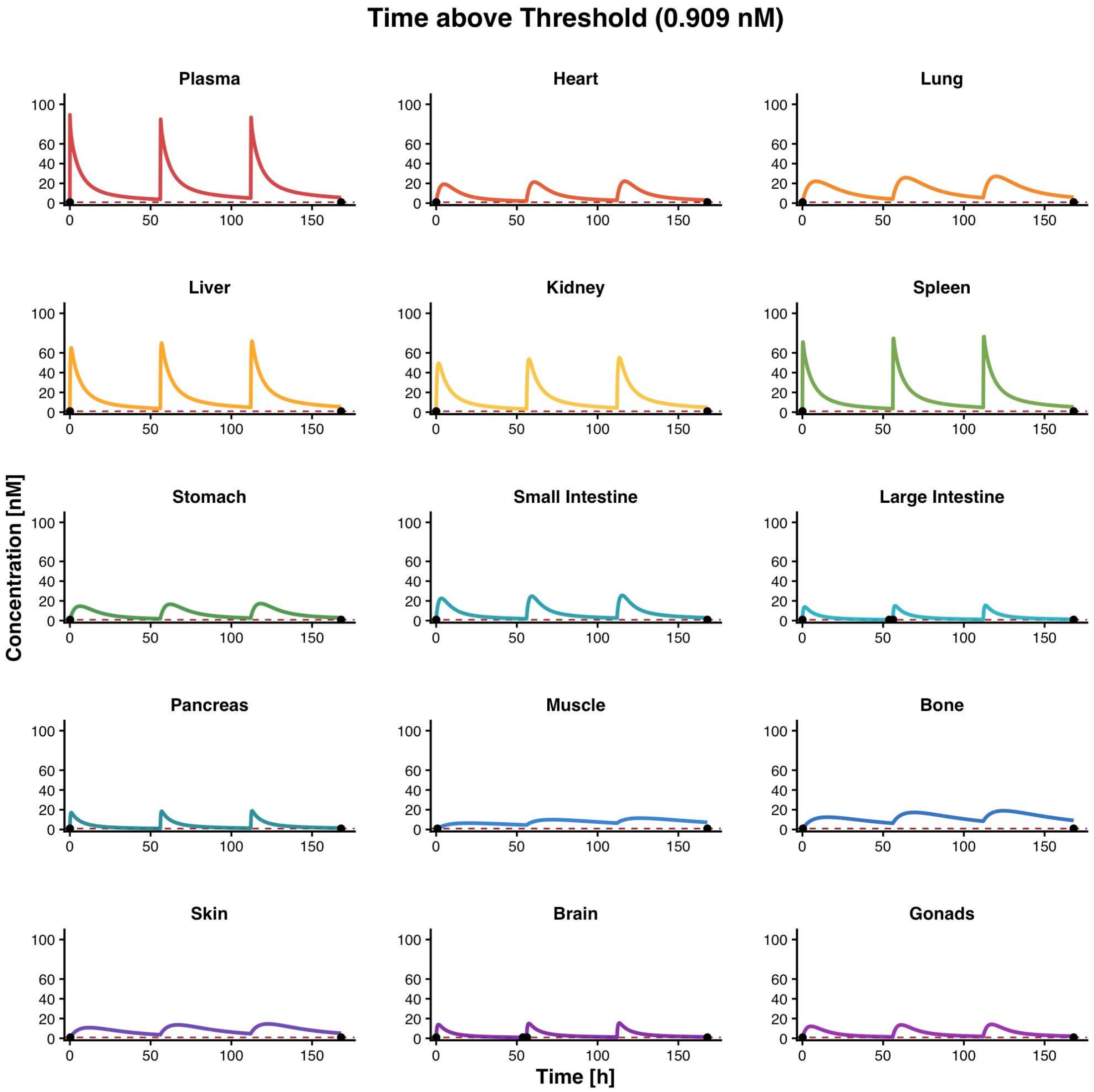

Figure S27. Time intervals during which FIX concentrations exceeded 1% in different tissues after three times-weekly dosing at 50 IU/kg. The y-axis represents FIX concentration (nM), and the x-axis represents time (t). The red line indicates the 1% concentration threshold. Black dots mark the start and end times at which FIX concentrations reached the 1% target.

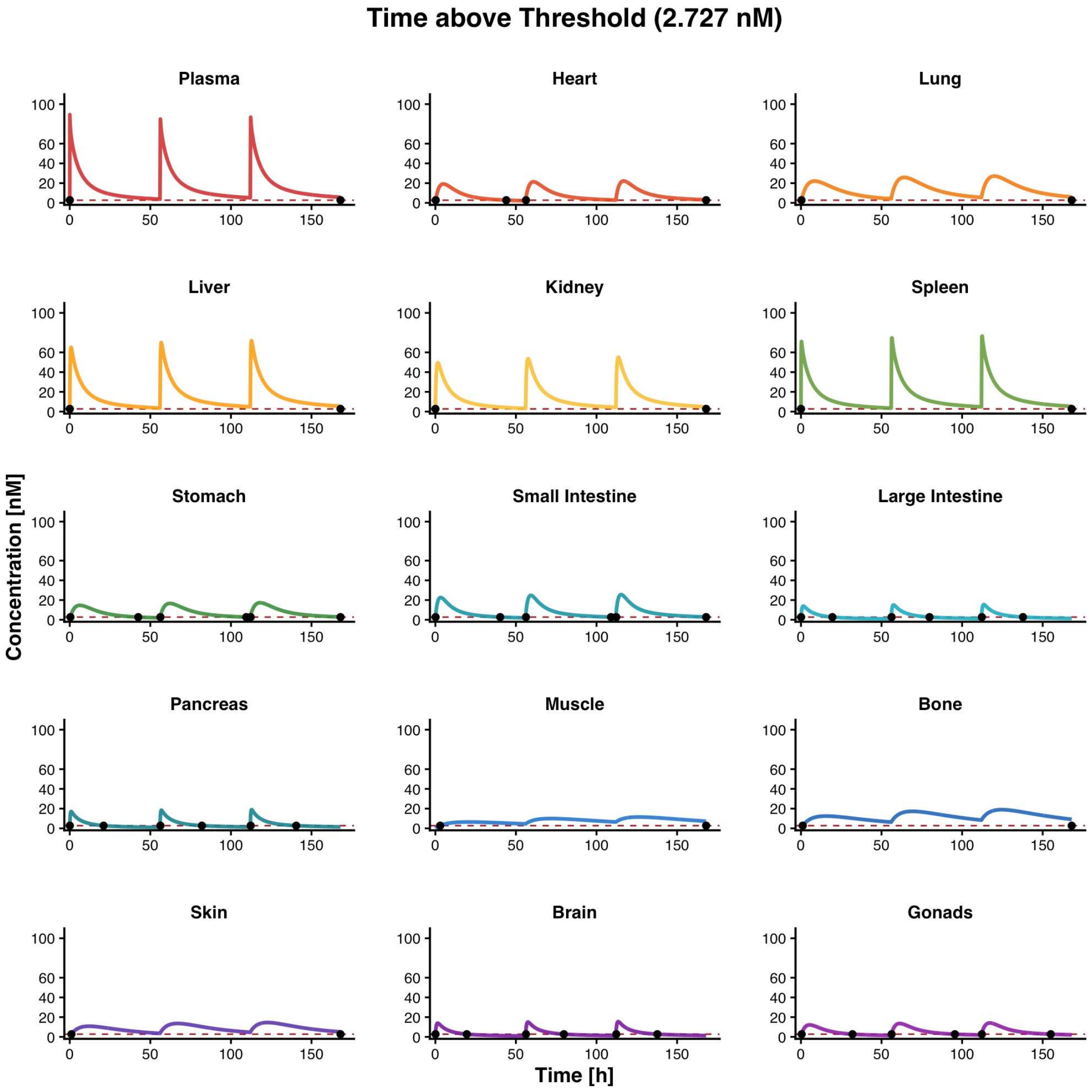

Figure S28. Time intervals during which FIX concentrations exceeded 3% in different tissues after three times-weekly dosing at 50 IU/kg. The y-axis represents FIX concentration (nM), and the x-axis represents time (t). The red line indicates the 3% concentration threshold. Black dots mark the start and end times at which FIX concentrations reached the 3% target.

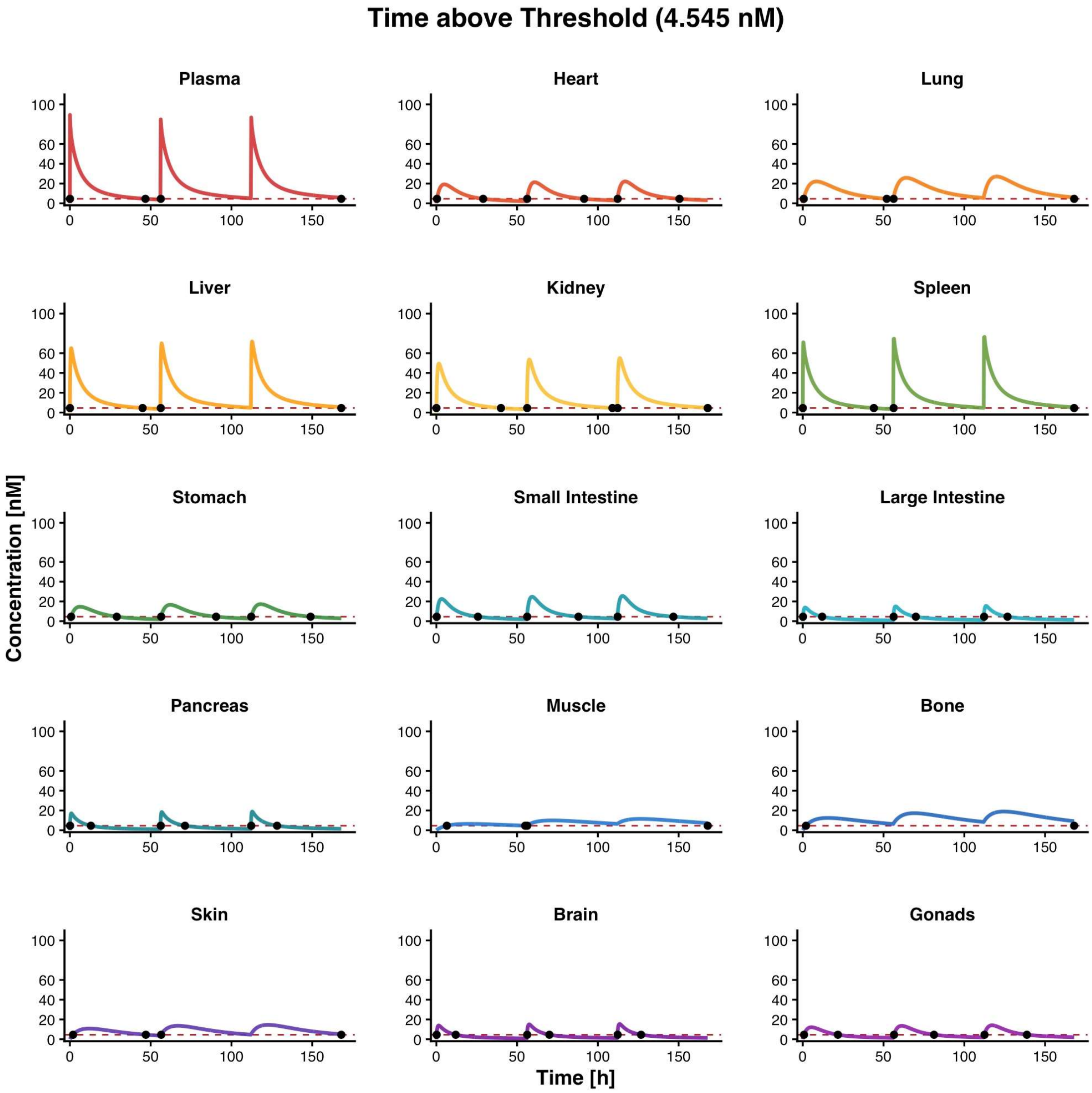

Figure S29. Time intervals during which FIX concentrations exceeded 5% in different tissues after three times-weekly dosing at 50 IU/kg. The y-axis represents FIX concentration (nM), and the x-axis represents time (t). The red line indicates the 5% concentration threshold. Black dots mark the start and end times at which FIX concentrations reached the 5% target.

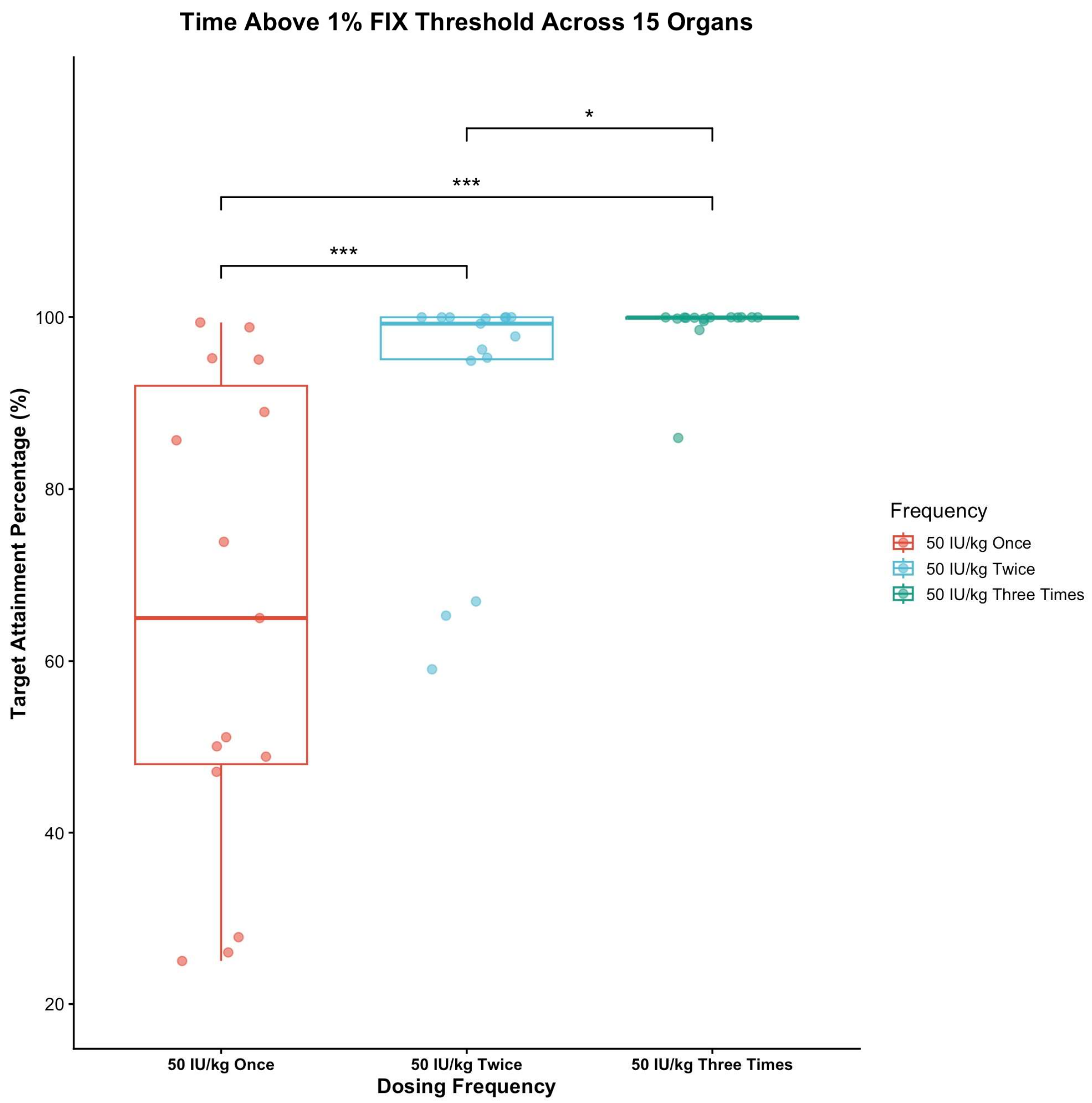

Figure S30. Box plots of target attainment rates (>1%) at different dosing frequencies. This figure summarizes differences in the rate of achieving FIX concentrations above 1% across tissues among the different dosing regimens. Red represents the 50 IU/kg once-weekly group, blue represents the 50 IU/kg twice-weekly group, and green represents the 50 IU/kg three-times-weekly group.

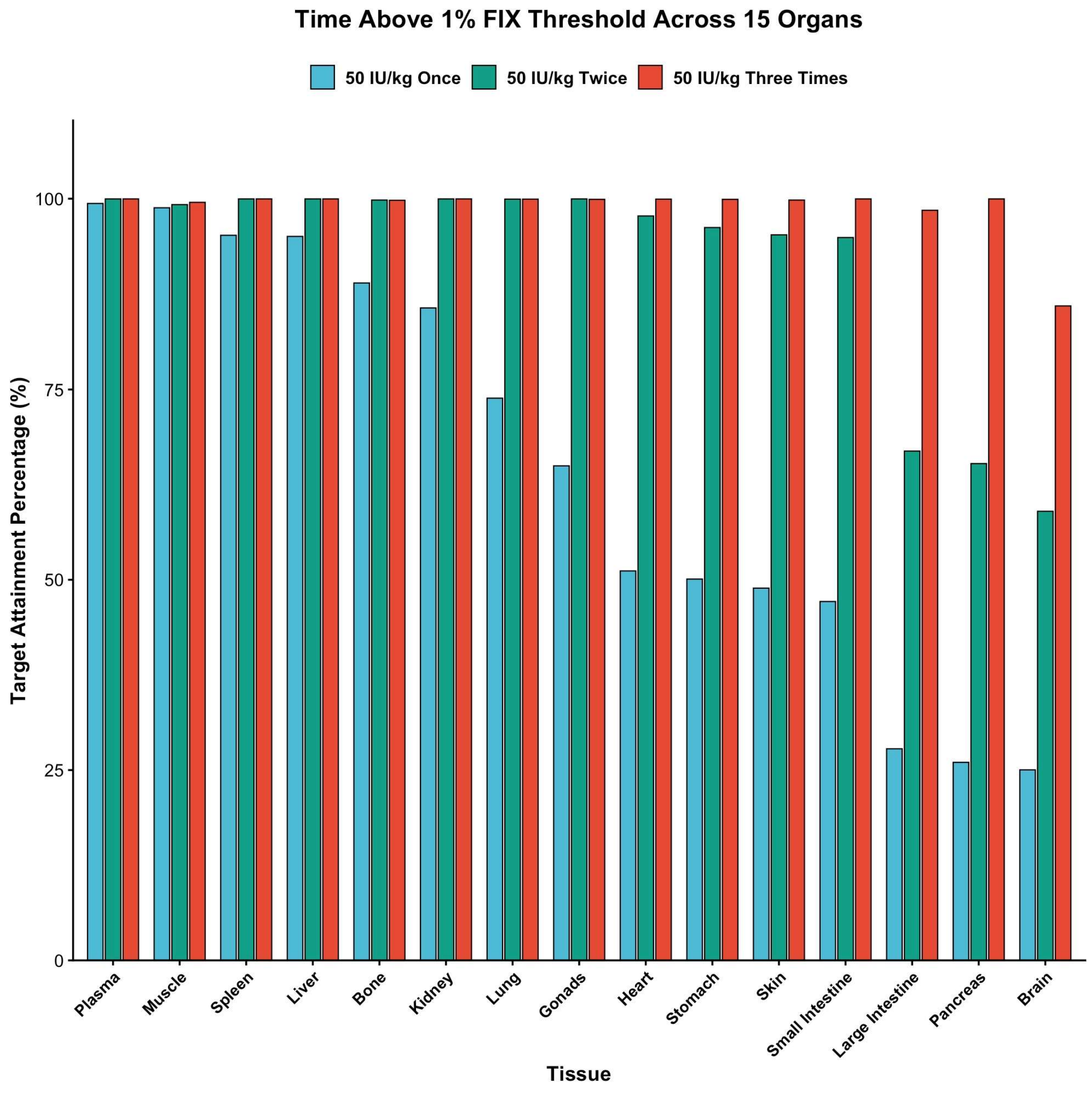

Figure S31. Bar plots of target attainment rates (>1%) at different dosing frequencies. This figure shows differences in the rate of achieving FIX concentrations above 1% across tissues under different dosing regimens. Blue represents the 50 IU/kg once-weekly group, green represents the 50 IU/kg twice-weekly group, and red represents the 50 IU/kg three-times-weekly group.

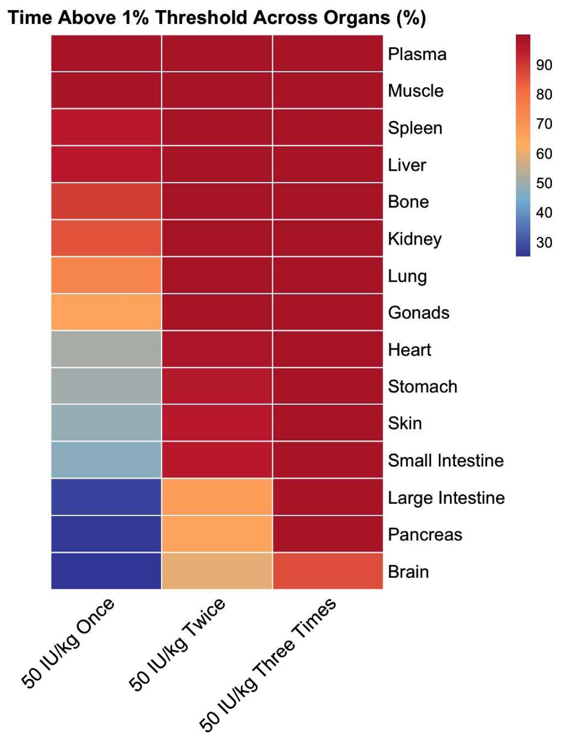

Figure S32. Heatmap of target attainment rates (>1%) at different dosing frequencies. This figure shows differences in the rate of achieving FIX concentrations above 1% across tissues under different dosing regimens. Colors range from blue to red, indicating increasing target attainment. From left to right, the panels represent the 50 IU/kg once-weekly, 50 IU/kg twice-weekly, and 50 IU/kg three-times-weekly groups.

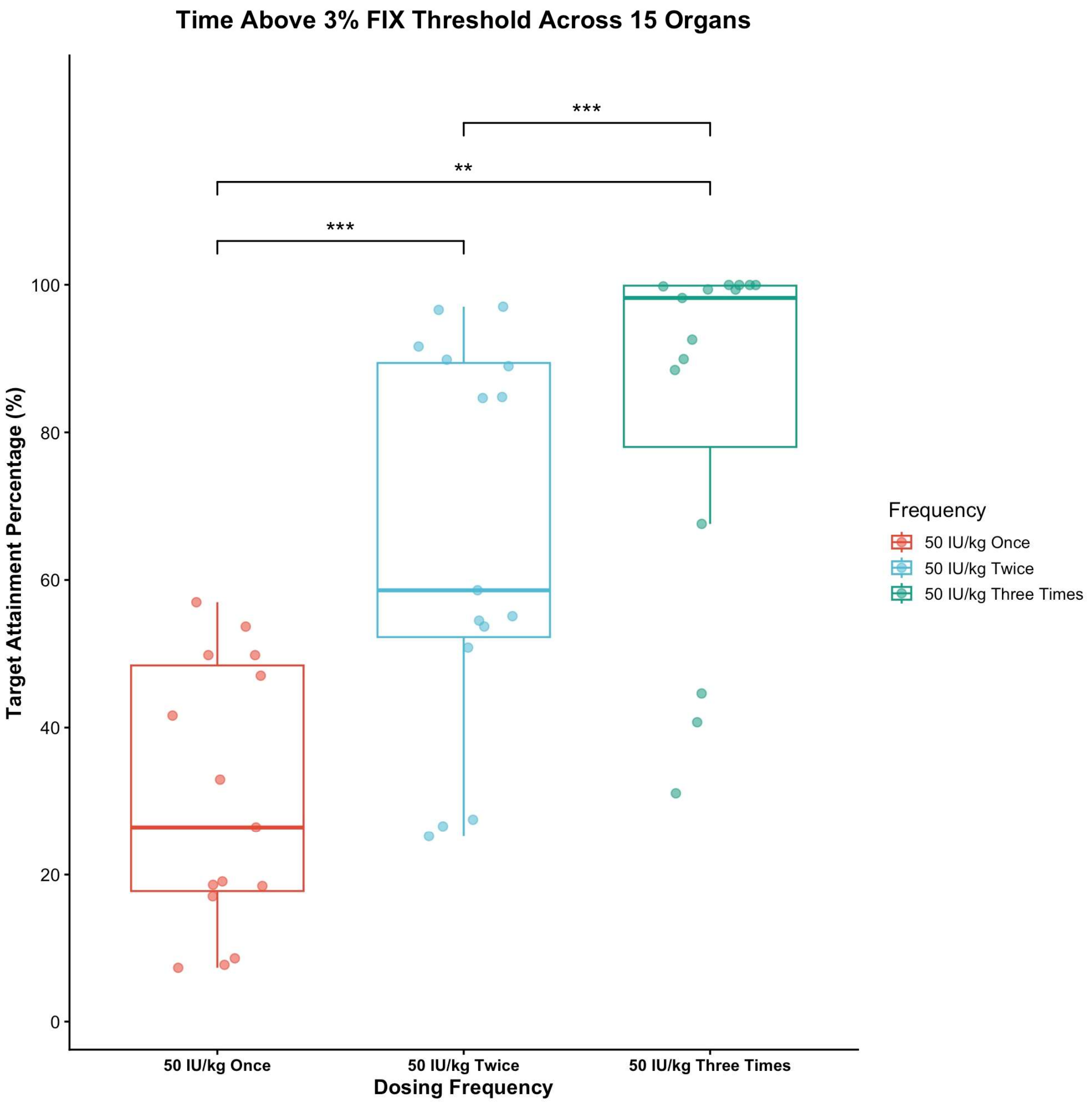

Figure S33. Box plots of target attainment rates (>3%) at different dosing frequencies. This figure summarizes differences in the rate of achieving FIX concentrations above 3% across tissues among the different dosing regimens. Red represents the 50 IU/kg once-weekly group, blue represents the 50 IU/kg twice-weekly group, and green represents the 50 IU/kg three-times-weekly group.

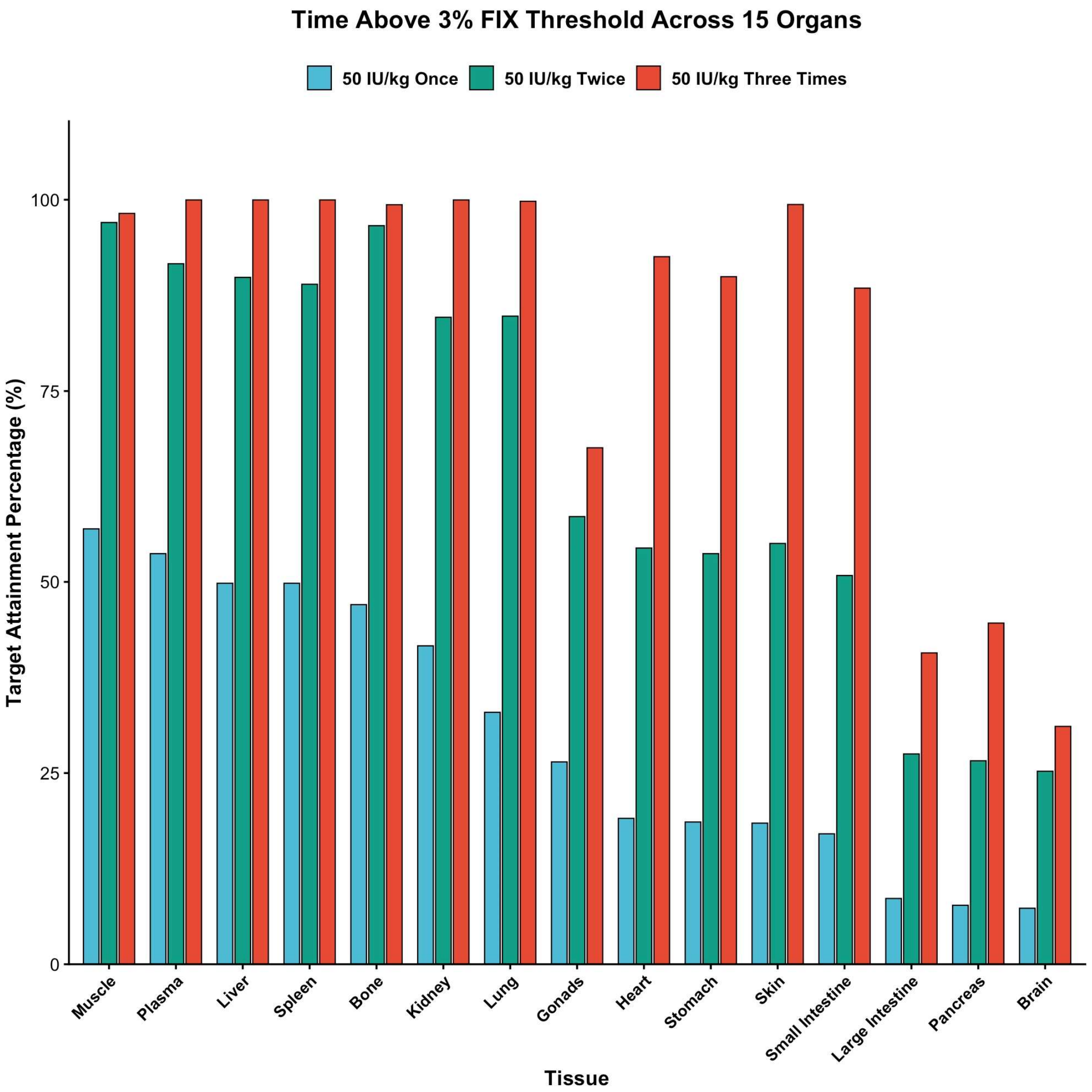

Figure S34. Bar plots of target attainment rates (>3%) at different dosing frequencies. This figure shows differences in the rate of achieving FIX concentrations above 3% across tissues under different dosing regimens. Blue represents the 50 IU/kg once-weekly group, green represents the 50 IU/kg twice-weekly group, and red represents the 50 IU/kg three-times-weekly group.

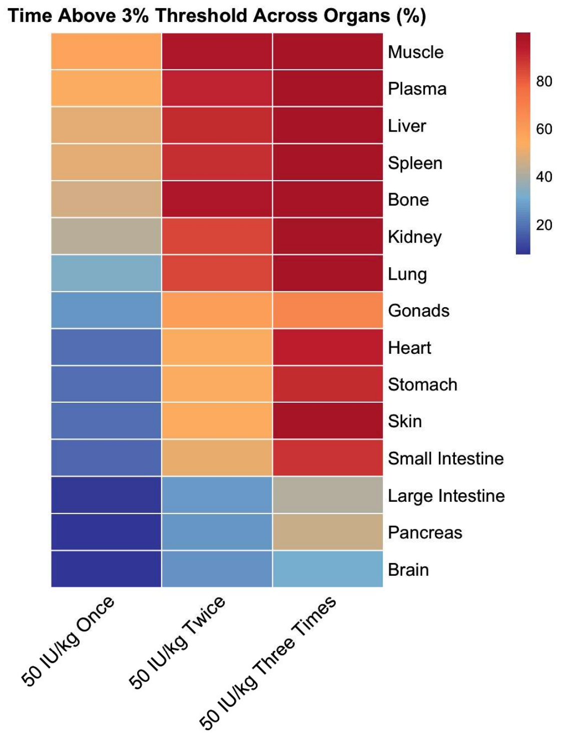

Figure S35. Heatmap of target attainment rates (>3%) at different dosing frequencies. This figure shows differences in the rate of achieving FIX concentrations above 3% across tissues under different dosing regimens. Colors range from blue to red, indicating increasing target attainment. From left to right, the panels represent the 50 IU/kg once-weekly, 50 IU/kg twice-weekly, and 50 IU/kg three-times-weekly groups.

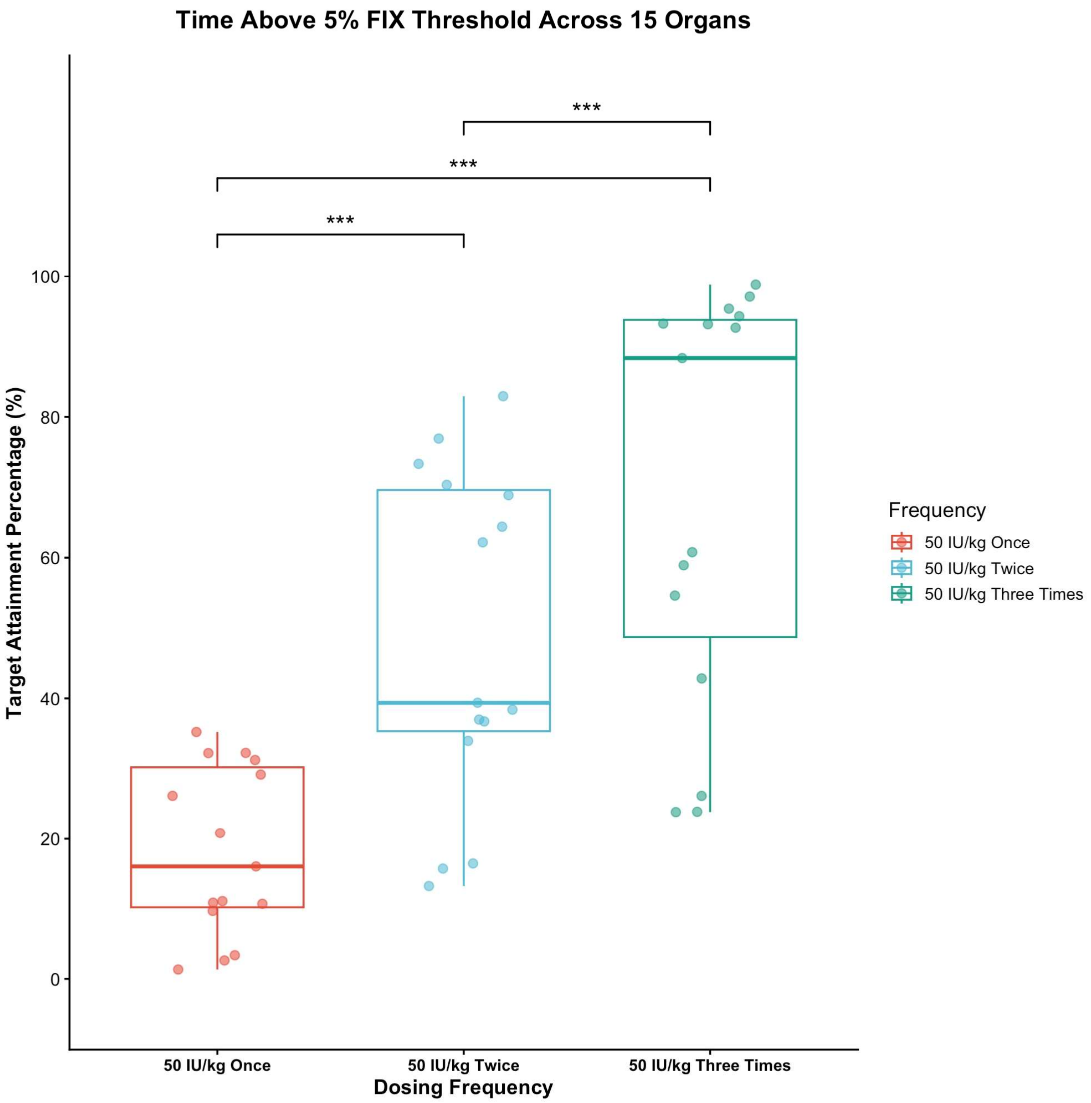

Figure S36. Box plots of target attainment rates (>5%) at different dosing frequencies. This figure summarizes differences in the rate of achieving FIX concentrations above 5% across tissues among the different dosing regimens. Red represents the 50 IU/kg once-weekly group, blue represents the 50 IU/kg twice-weekly group, and green represents the 50 IU/kg three-times-weekly group.

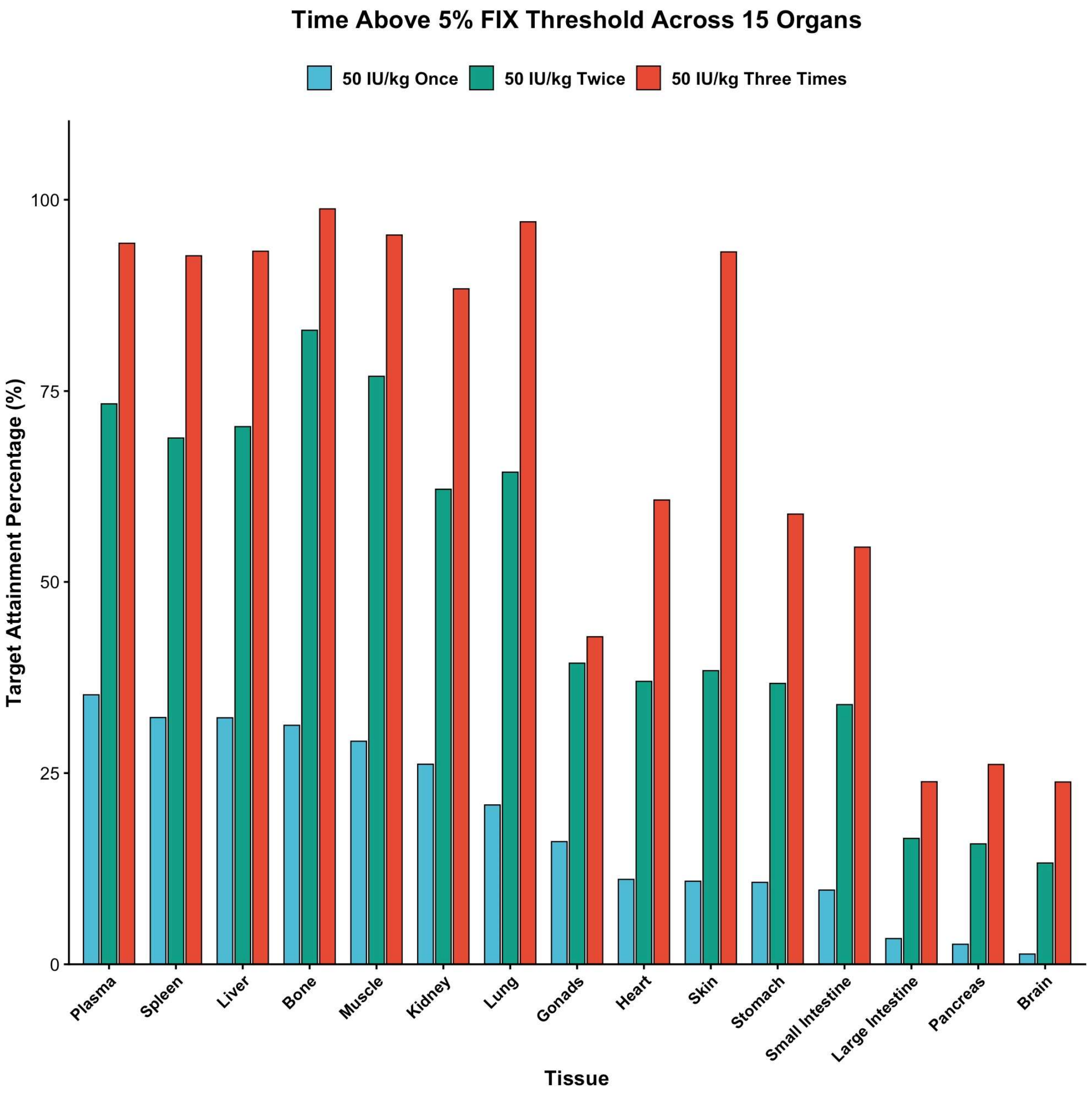

Figure S37. Bar plots of target attainment rates (>5%) at different dosing frequencies. This figure shows differences in the rate of achieving FIX concentrations above 5% across tissues under different dosing regimens. Blue represents the 50 IU/kg once-weekly group, green represents the 50 IU/kg twice-weekly group, and red represents the 50 IU/kg three-times-weekly group.

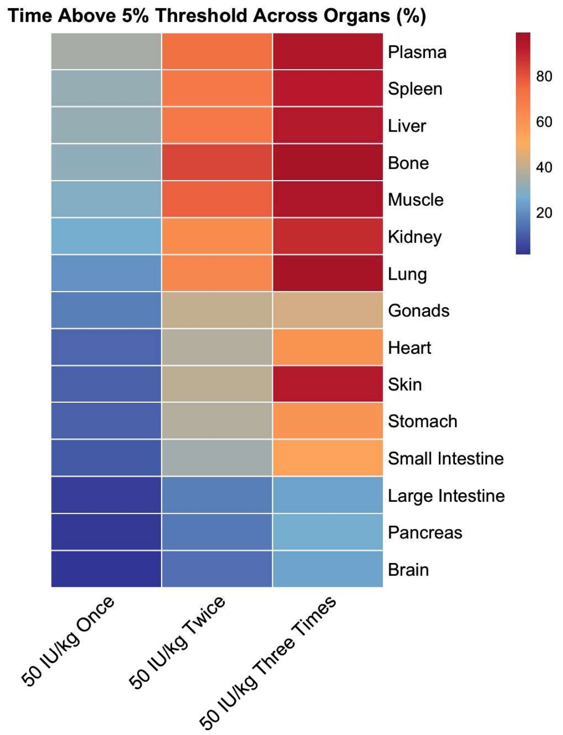

Figure S38. Heatmap of target attainment rates (>5%) at different dosing frequencies. This figure shows differences in the rate of achieving FIX concentrations above 5% across tissues under different dosing regimens. Colors range from blue to red, indicating increasing target attainment. From left to right, the panels represent the 50 IU/kg once-weekly, 50 IU/kg twice-weekly, and 50 IU/kg three-times-weekly groups.

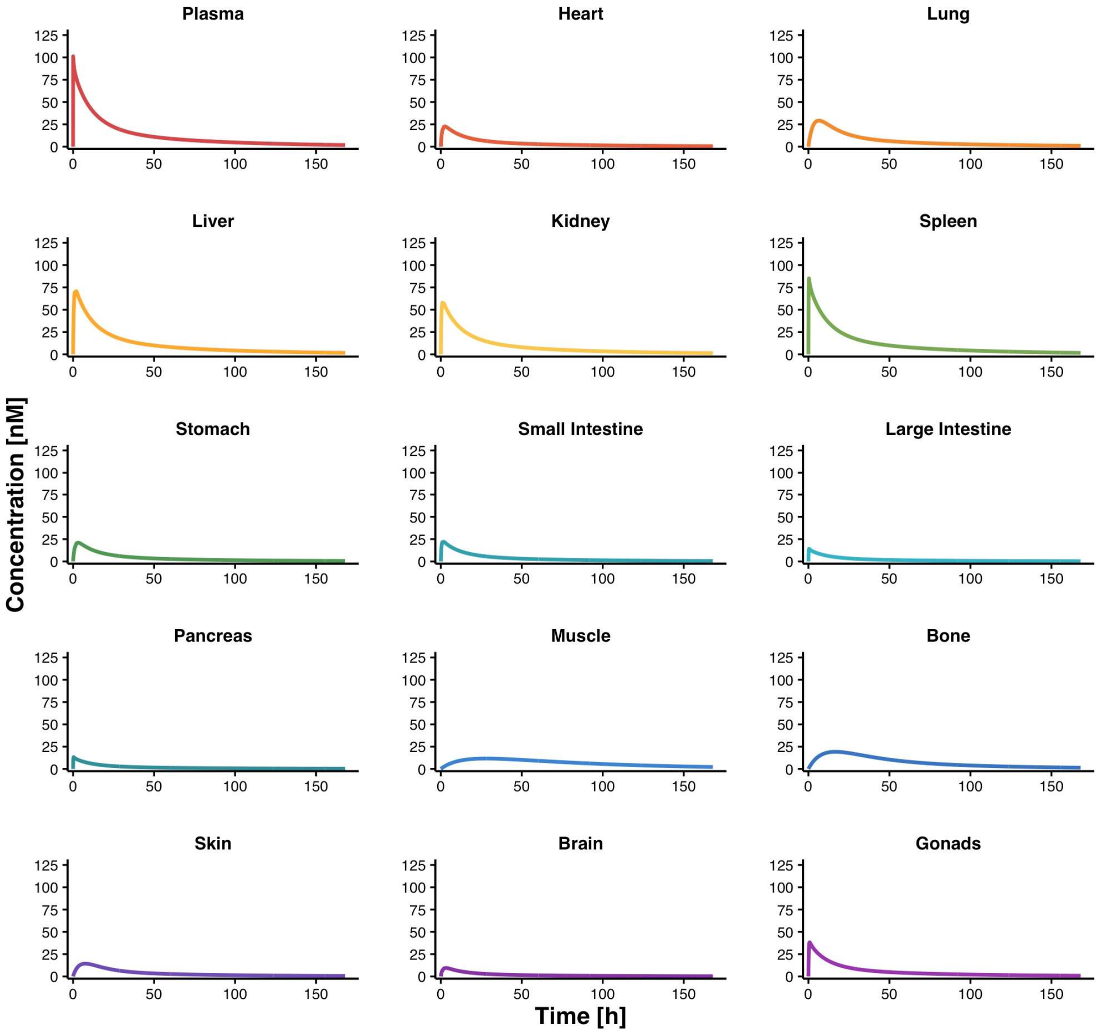

Figure S39. FIX distribution profiles under 100 IU/kg Once-weekly dosing. The y-axis represents FIX concentration (nM), and the x-axis represents time (t). This figure shows the distribution of FIX across tissues under 100 IU/kg Once-weekly dosing regimen.

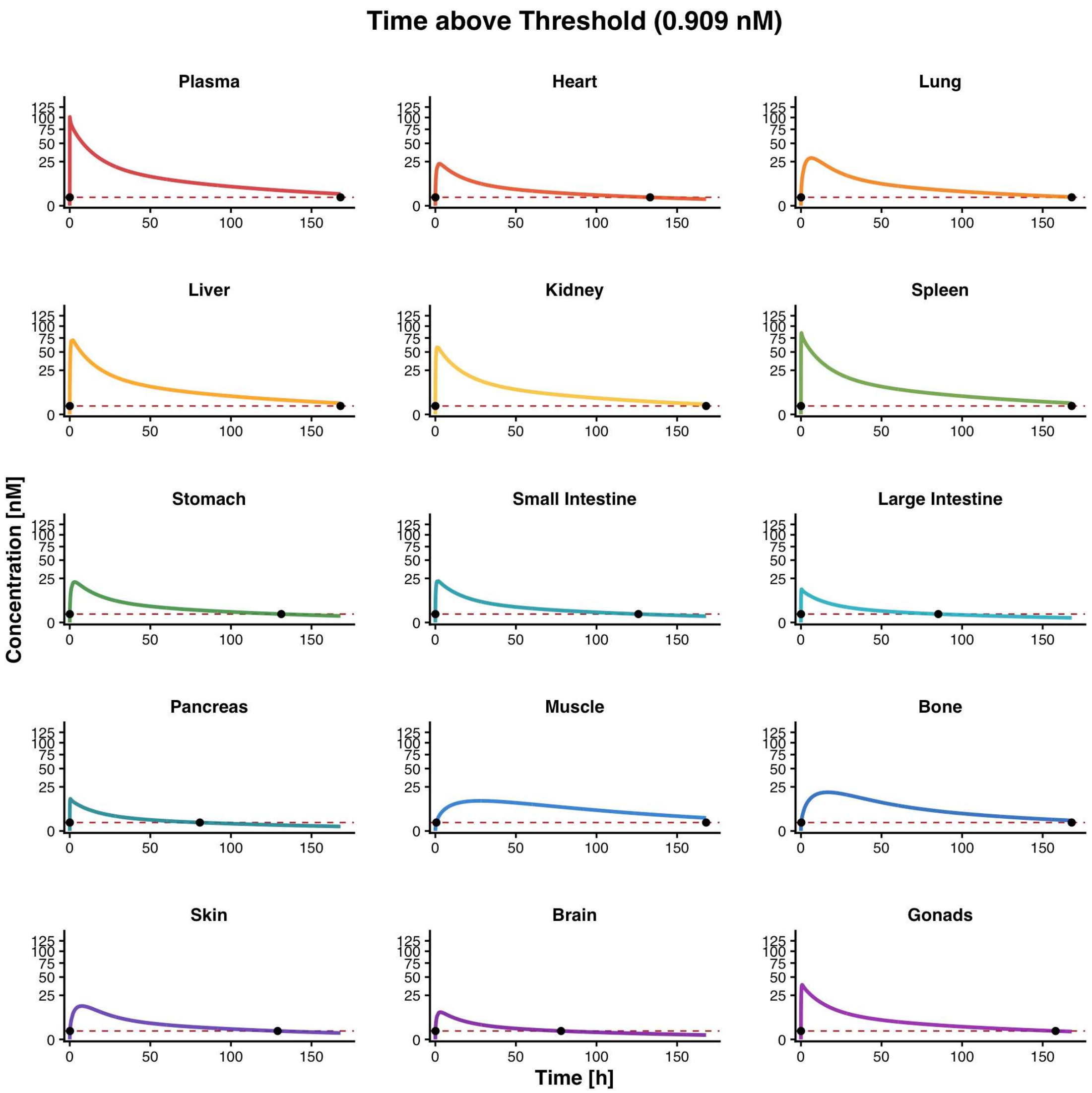

Figure S40. Time intervals during which FIX concentrations exceeded 1% in different tissues after Once-weekly dosing at 100 IU/kg. The y-axis represents FIX concentration (nM), and the x-axis represents time (t). The red line indicates the 1% concentration threshold. Black dots mark the start and end times at which FIX concentrations reached the 1% target.

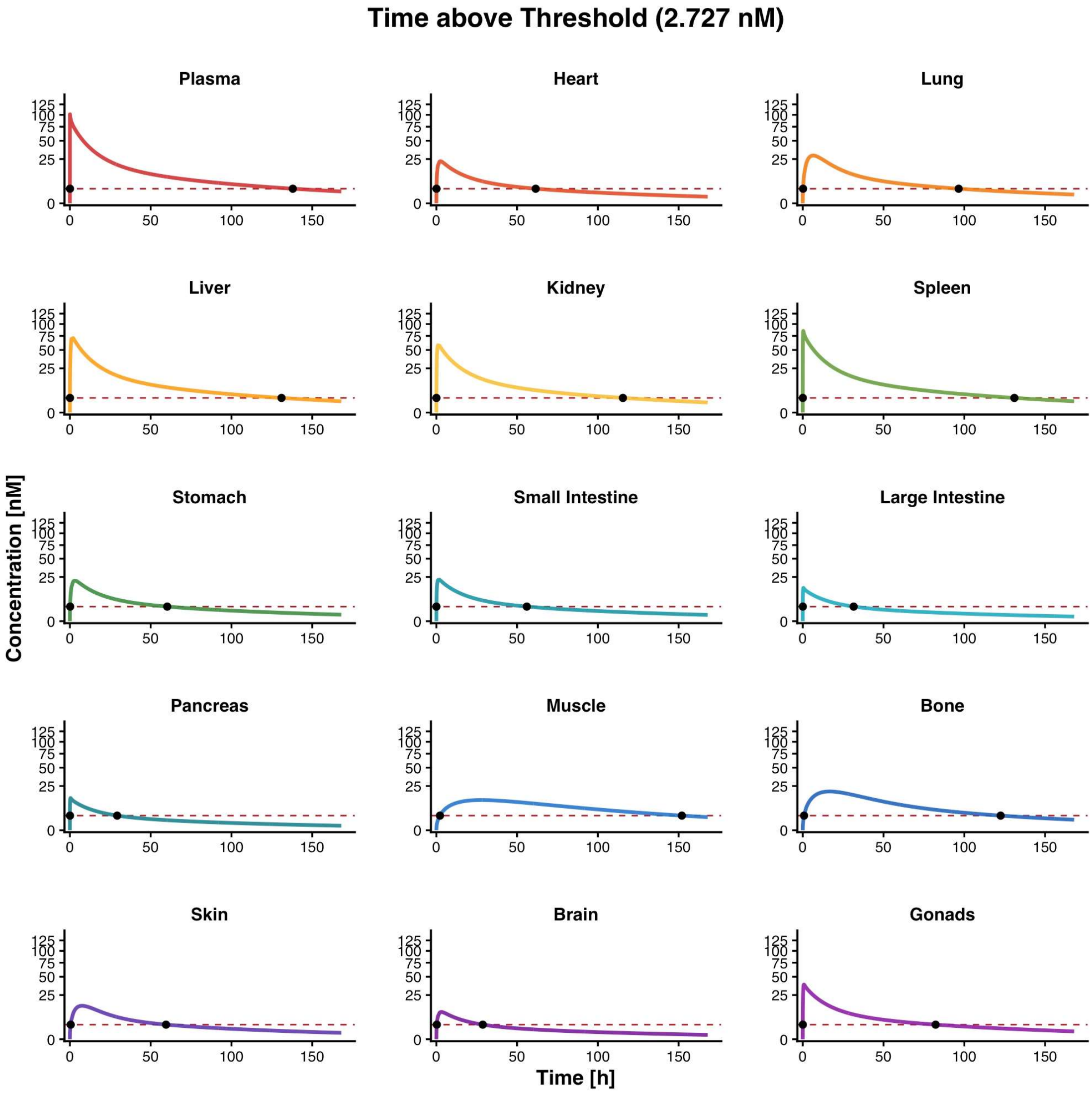

Figure S41. Time intervals during which FIX concentrations exceeded 3% in different tissues after Once-weekly dosing at 100 IU/kg. The y-axis represents FIX concentration (nM), and the x-axis represents time (t). The red line indicates the 3% concentration threshold. Black dots mark the start and end times at which FIX concentrations reached the 3% target.

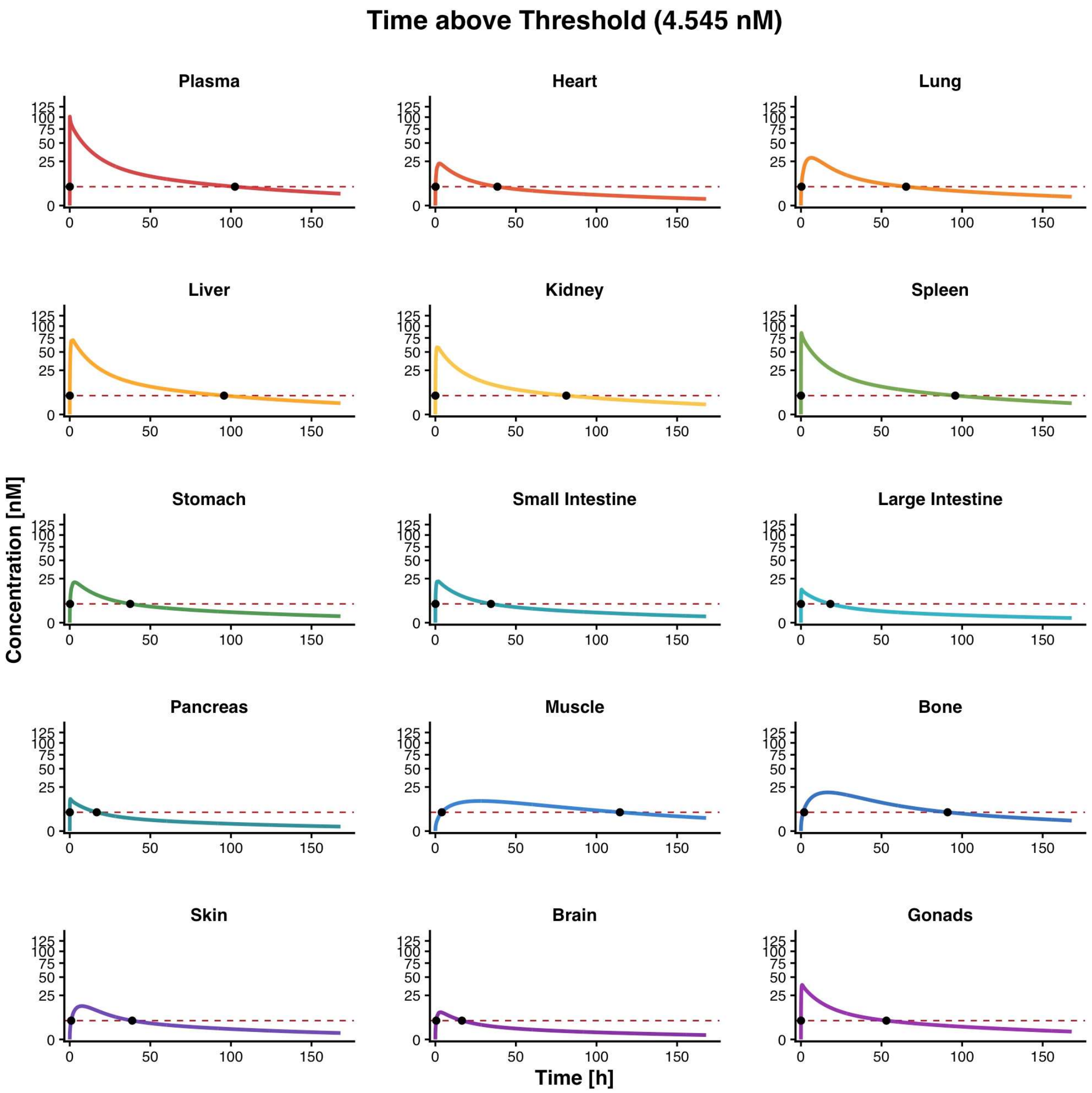

Figure S42. Time intervals during which FIX concentrations exceeded 5% in different tissues after Once-weekly dosing at 100 IU/kg. The y-axis represents FIX concentration (nM), and the x-axis represents time (t). The red line indicates the 5% concentration threshold. Black dots mark the start and end times at which FIX concentrations reached the 5% target.

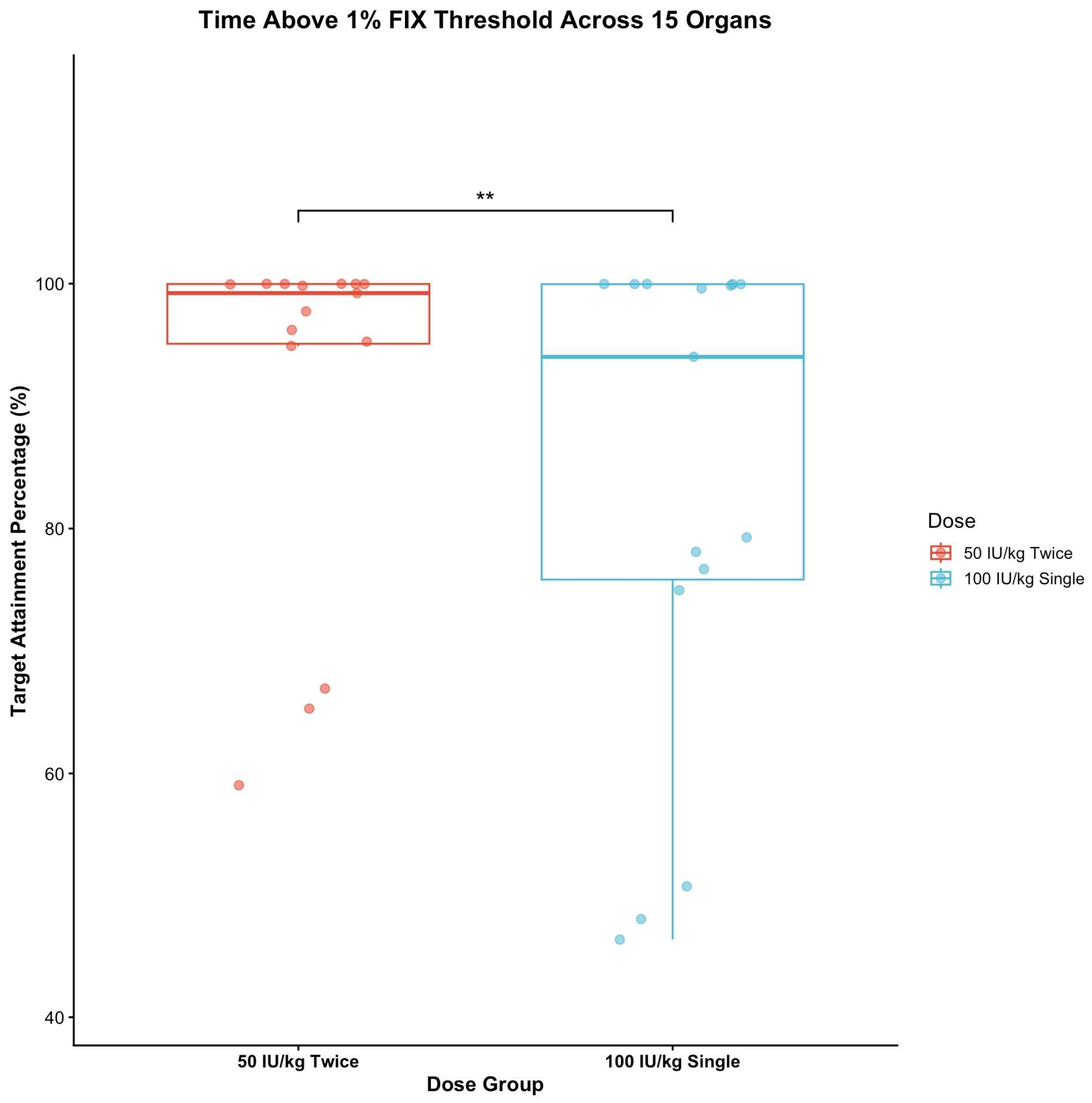

Figure S43. Box plots of target attainment rates (>1%) comparing dose doubling with increased dosing frequency under the same total weekly dose. This figure summarizes differences in the rate of achieving FIX concentrations above 1% across tissues between the two dosing regimens. Red represents the 50 IU/kg twice-weekly group, and blue represents the 100 IU/kg once-weekly group.

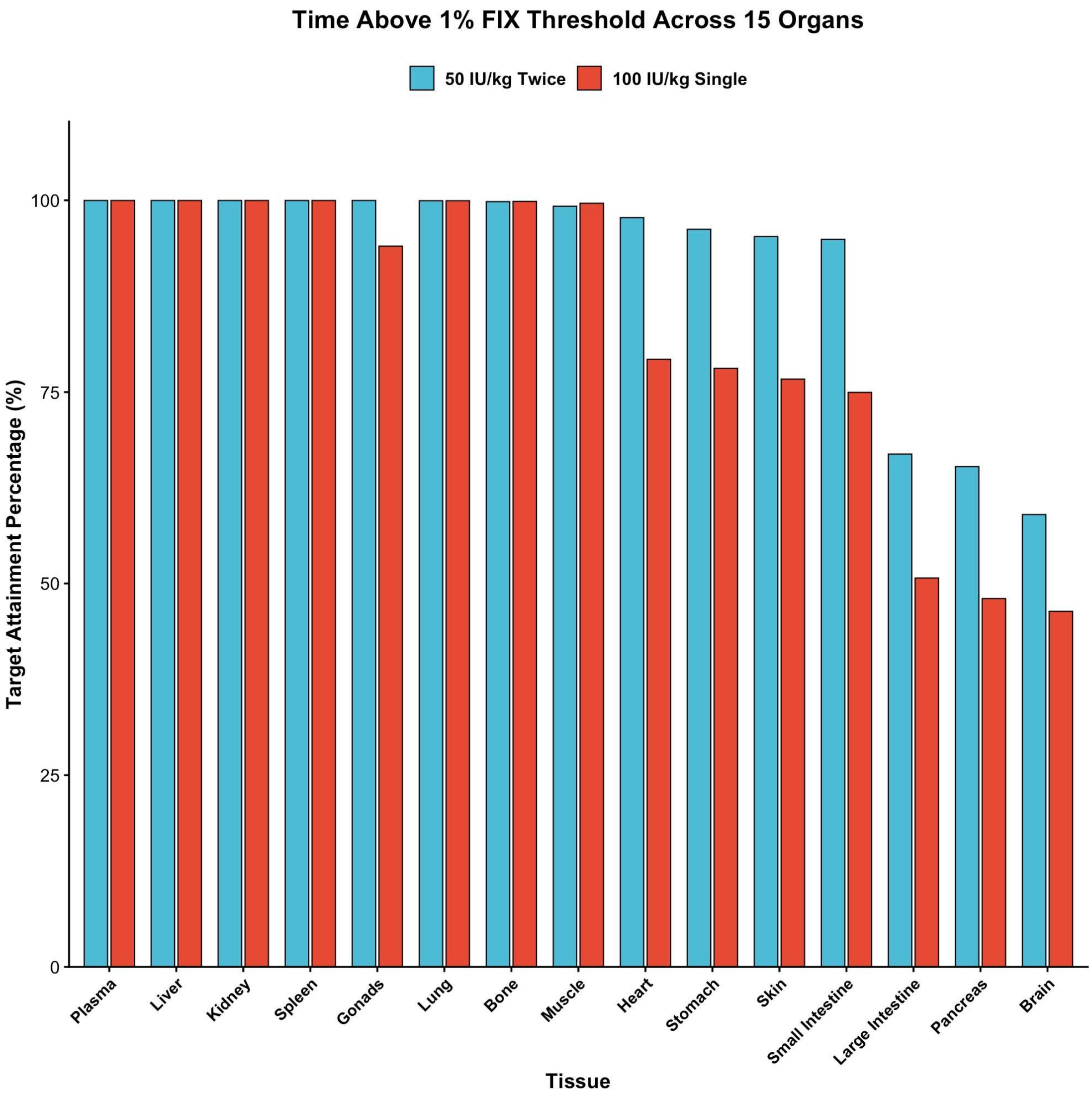

Figure S44. Bar plots of target attainment rates (>1%) comparing dose doubling with increased dosing frequency under the same total weekly dose. This figure shows differences in the rate of achieving FIX concentrations above 1% across tissues under different dosing regimens. Blue represents the 50 IU/kg twice-weekly group, and red represents the 100 IU/kg once-weekly group.

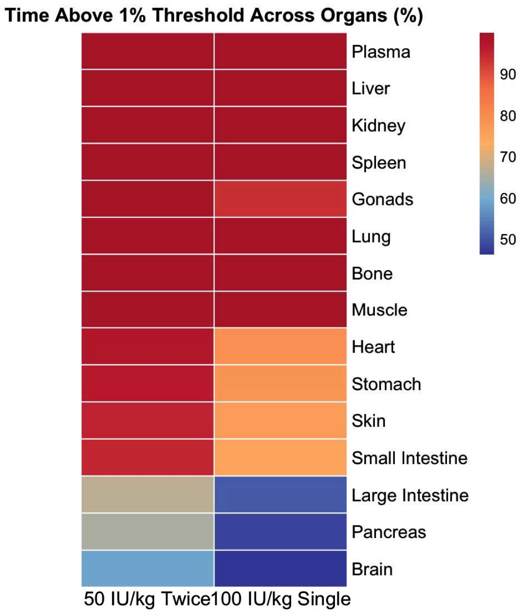

Figure S45. Heatmap of target attainment rates (>1%) comparing dose doubling with increased dosing frequency under the same total weekly dose. This figure shows differences in the rate of achieving FIX concentrations above 1% across tissues under different dosing regimens. Colors range from blue to red, indicating increasing target attainment. From left to right, the panels represent the 50 IU/kg twice-weekly, and 100 IU/kg once-weekly groups.

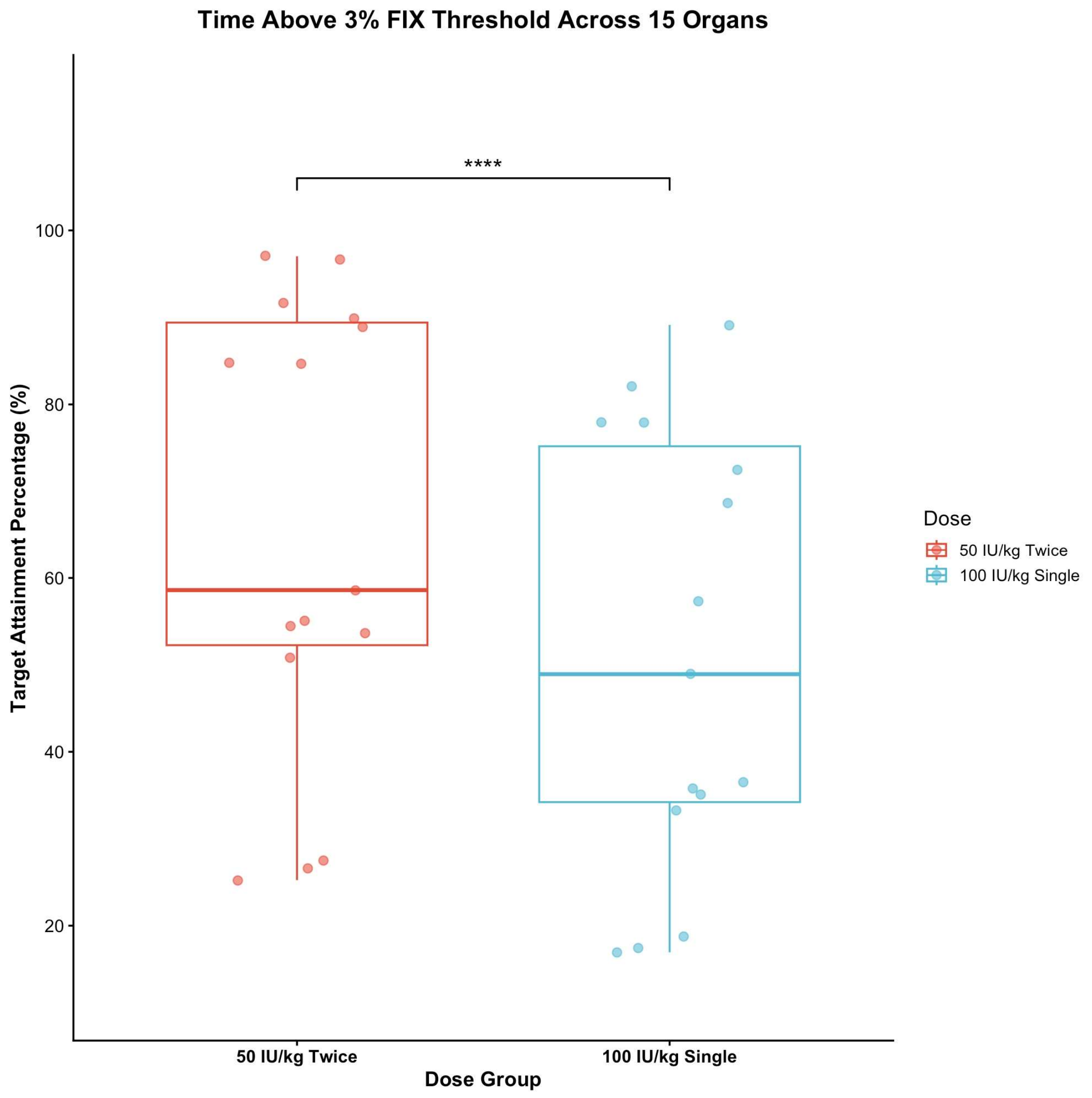

Figure S46. Box plots of target attainment rates (>3%) comparing dose doubling with increased dosing frequency under the same total weekly dose. This figure summarizes differences in the rate of achieving FIX concentrations above 3% across tissues between the two dosing regimens. Red represents the 50 IU/kg twice-weekly group, and blue represents the 100 IU/kg once-weekly group.

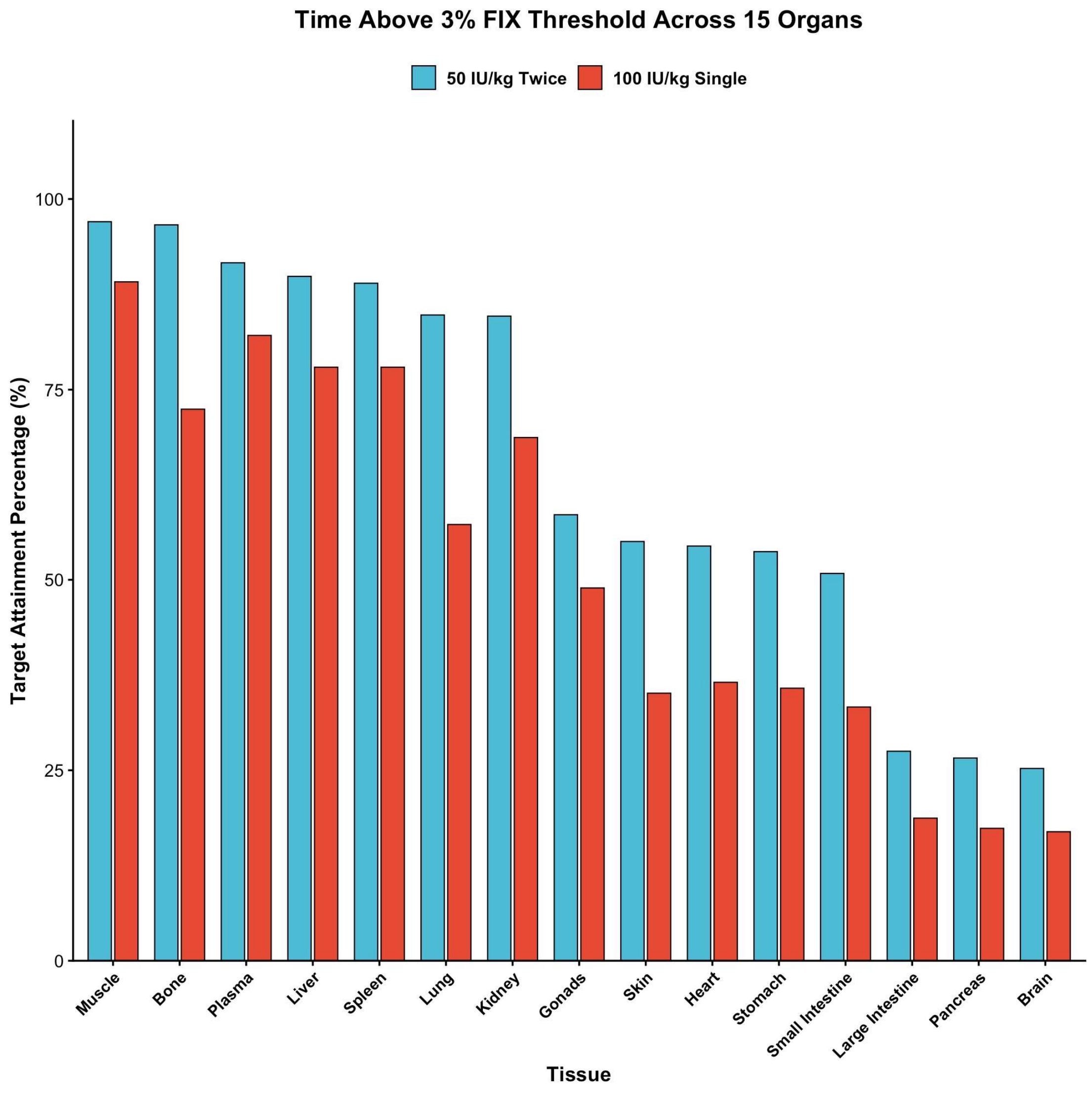

Figure S47. Bar plots of target attainment rates (>3%) comparing dose doubling with increased dosing frequency under the same total weekly dose. This figure shows differences in the rate of achieving FIX concentrations above 3% across tissues under different dosing regimens. Blue represents the 50 IU/kg twice-weekly group, and red represents the 100 IU/kg once-weekly group.

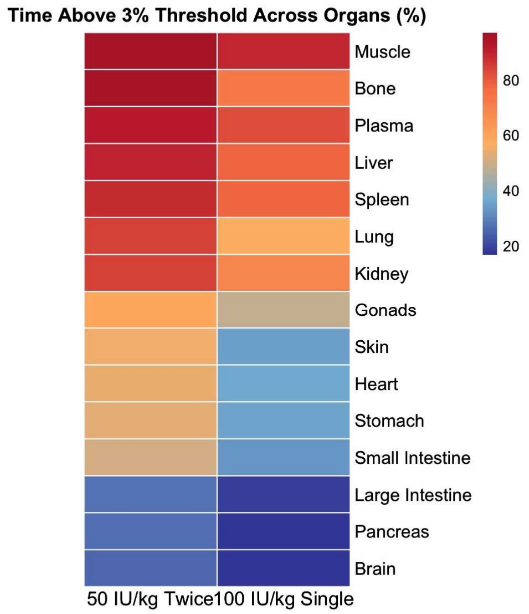

Figure S48. Heatmap of target attainment rates (>3%) comparing dose doubling with increased dosing frequency under the same total weekly dose. This figure shows differences in the rate of achieving FIX concentrations above 3% across tissues under different dosing regimens. Colors range from blue to red, indicating increasing target attainment. From left to right, the panels represent the 50 IU/kg twice-weekly, and 100 IU/kg once-weekly groups.

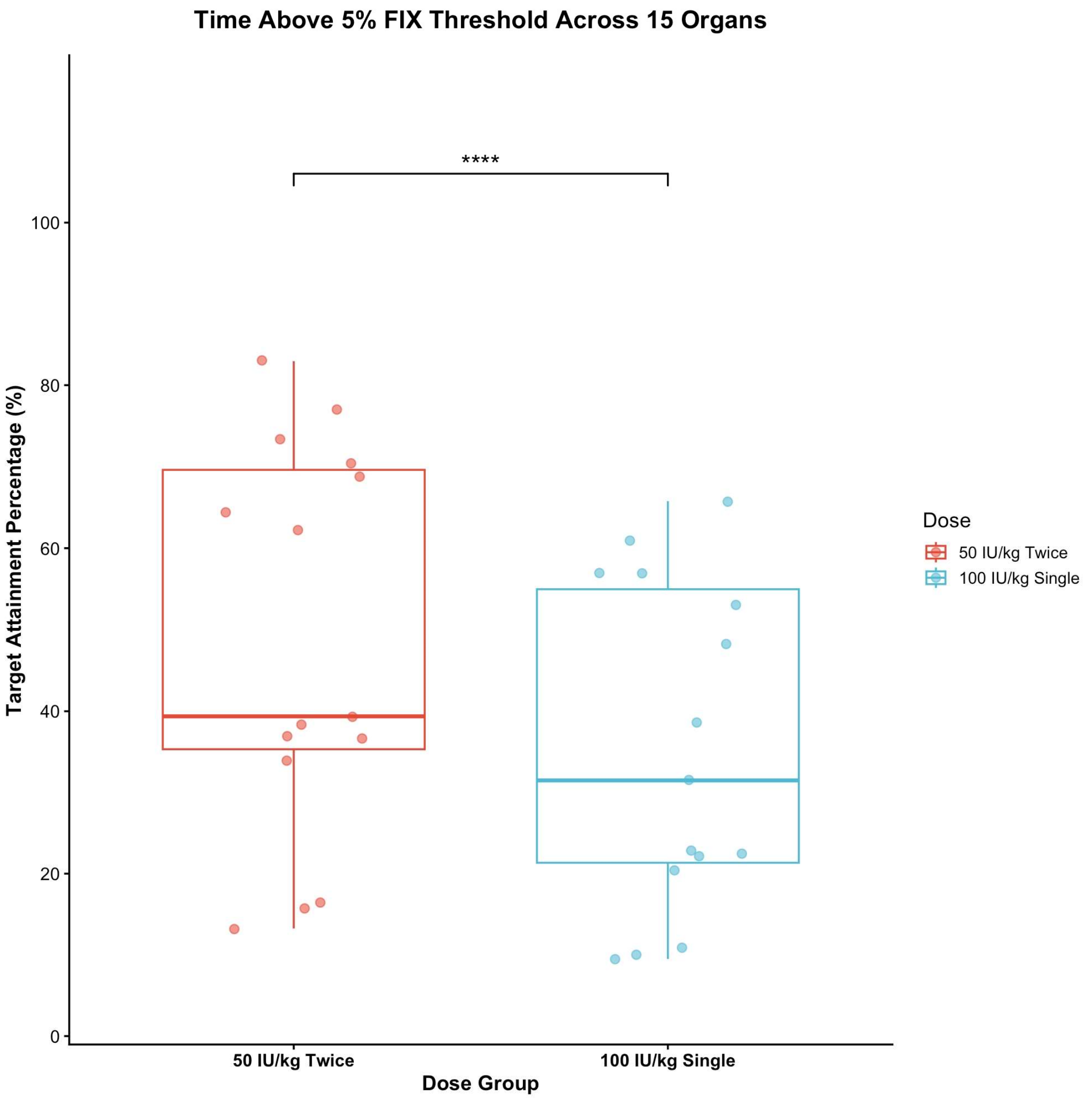

Figure S49. Box plots of target attainment rates (>5%) comparing dose doubling with increased dosing frequency under the same total weekly dose. This figure summarizes differences in the rate of achieving FIX concentrations above 5% across tissues between the two dosing regimens. Red represents the 50 IU/kg twice-weekly group, and blue represents the 100 IU/kg once-weekly group.

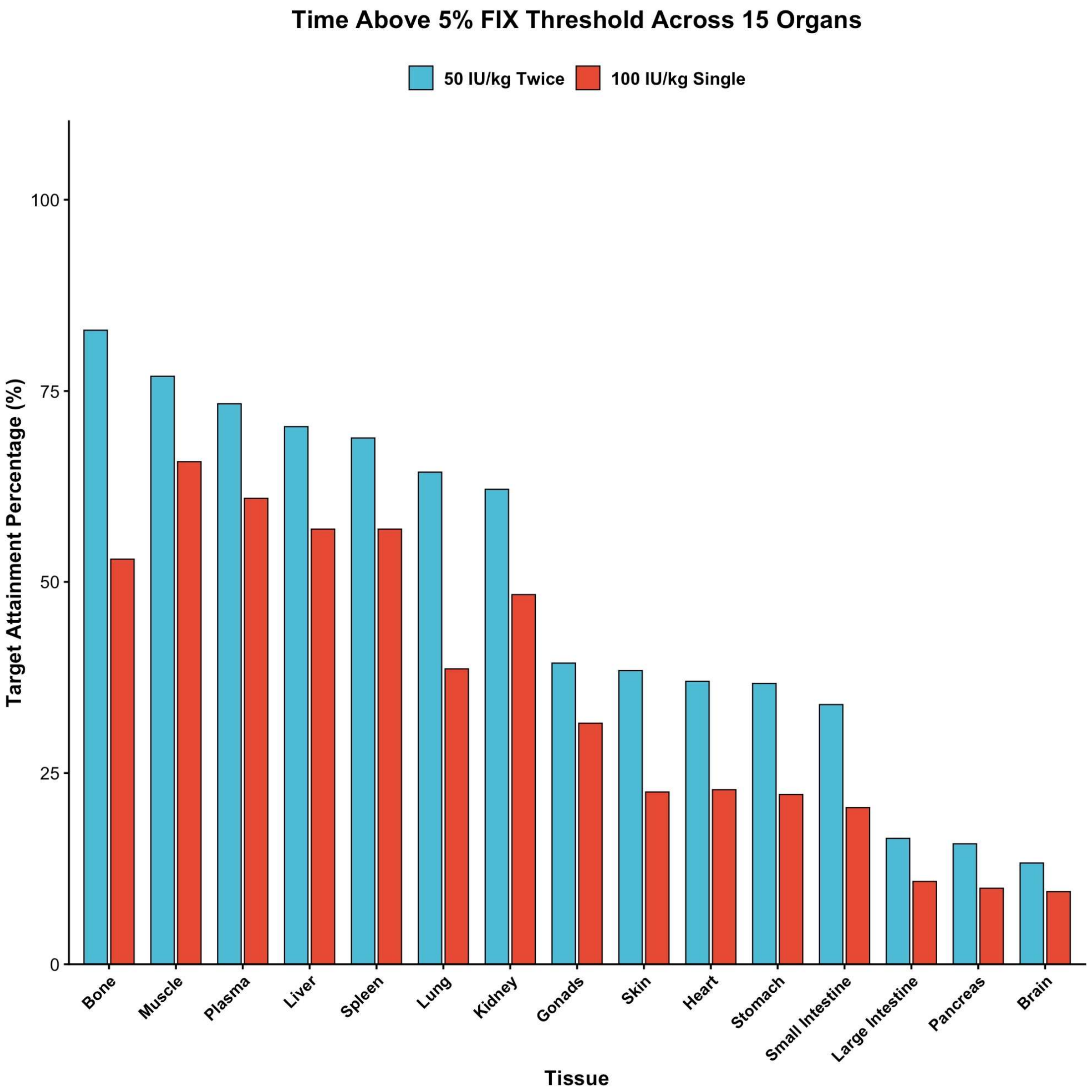

Figure S50. Bar plots of target attainment rates (>5%) comparing dose doubling with increased dosing frequency under the same total weekly dose. This figure shows differences in the rate of achieving FIX concentrations above 5% across tissues under different dosing regimens. Blue represents the 50 IU/kg twice-weekly group, and red represents the 100 IU/kg once-weekly group.

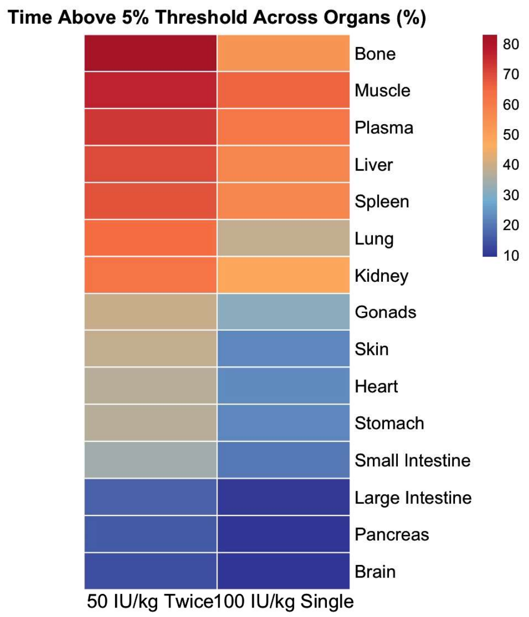

Figure S51. Heatmap of target attainment rates (>5%) comparing dose doubling with increased dosing frequency under the same total weekly dose. This figure shows differences in the rate of achieving FIX concentrations above 5% across tissues under different dosing regimens. Colors range from blue to red, indicating increasing target attainment. From left to right, the panels represent the 50 IU/kg twice-weekly, and 100 IU/kg once-weekly groups.

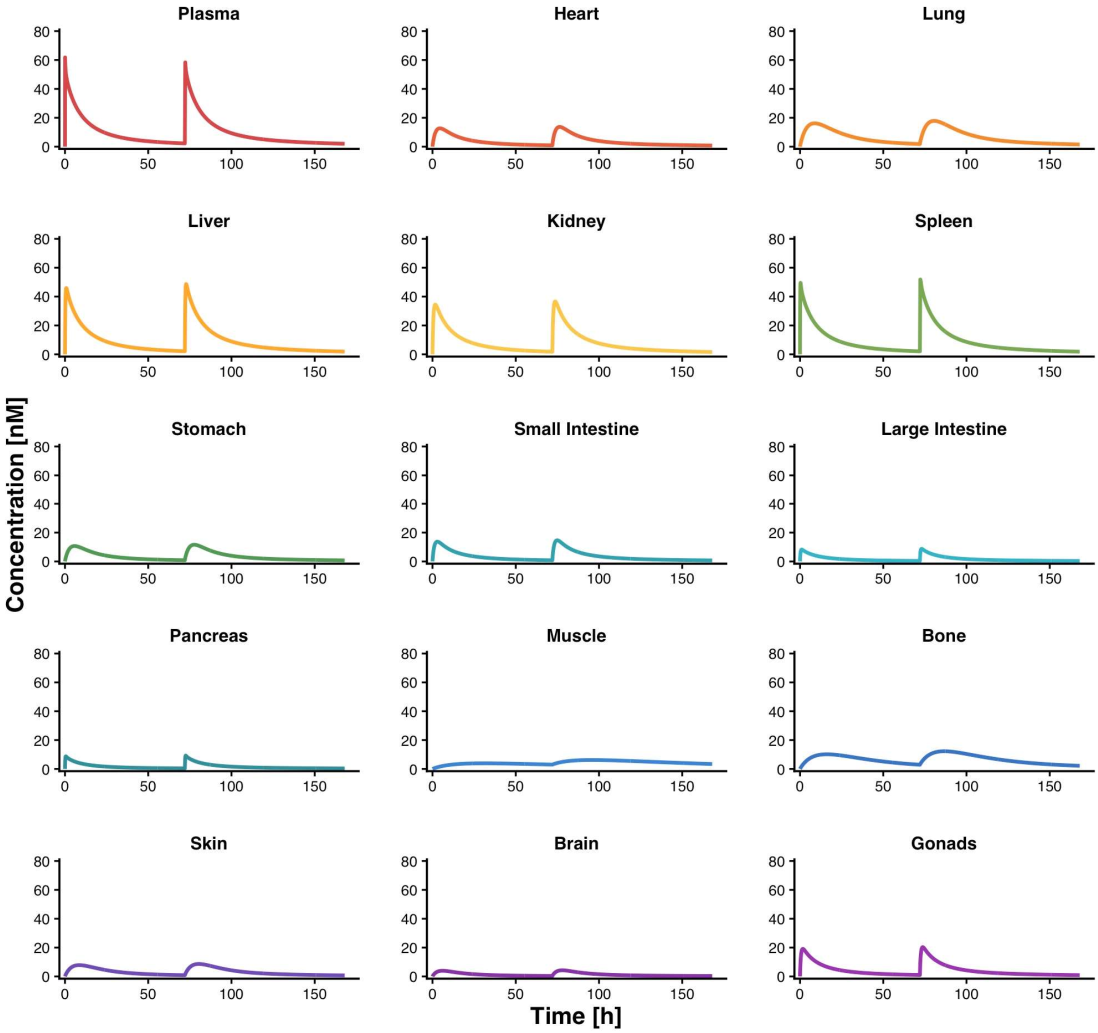

Figure S52. FIX distribution profiles under 100 IU/kg Once-weekly dosing. The y-axis represents FIX concentration (nM), and the x-axis represents time (t). This figure shows the distribution of FIX across tissues under 100 IU/kg Once-weekly dosing regimen.

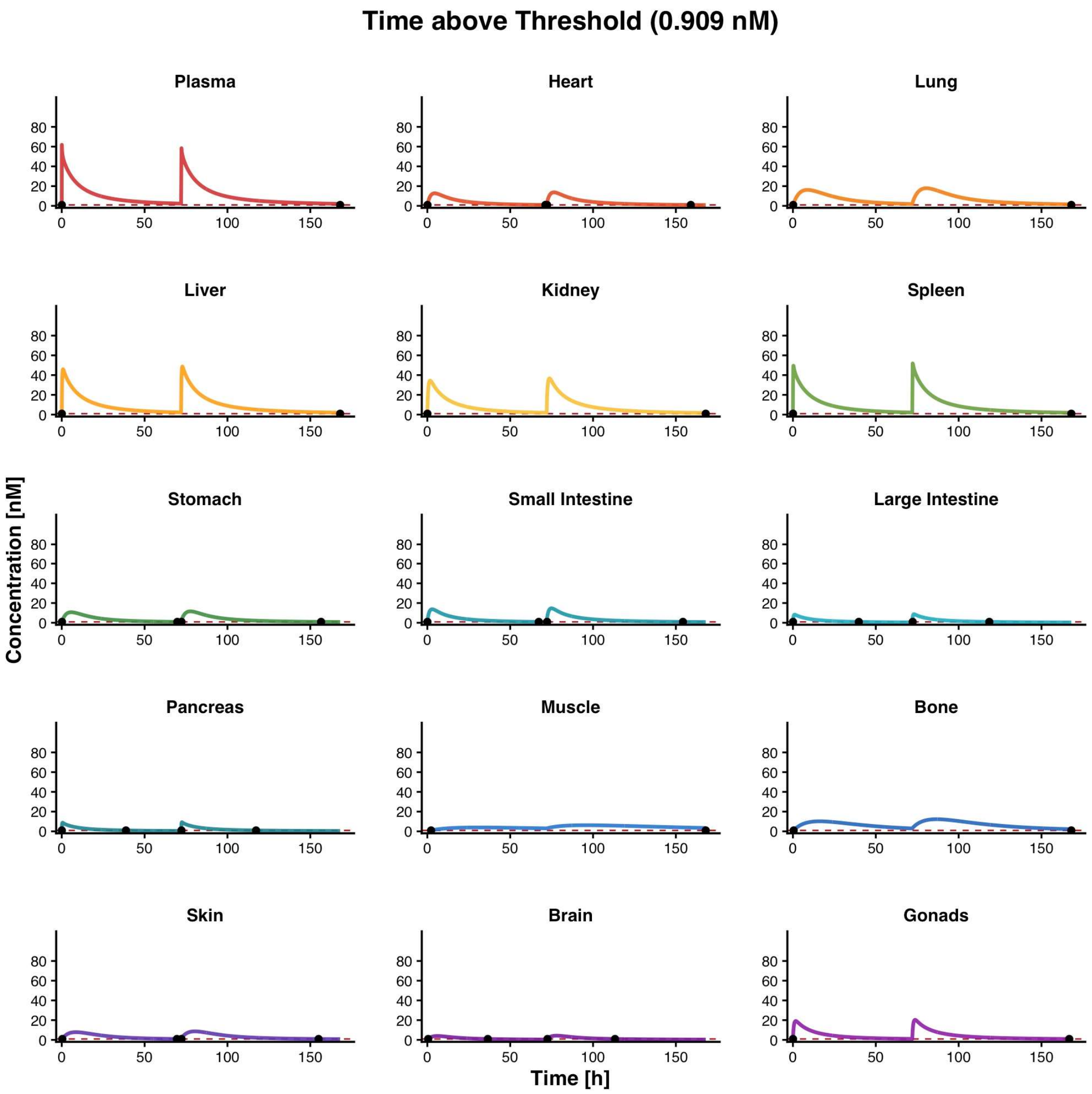

Figure S53. Time intervals during which FIX concentrations exceeded 1% in different tissues after twice-weekly dosing at 50 IU/kg (72h). The y-axis represents FIX concentration (nM), and the x-axis represents time (t). The red line indicates the 1% concentration threshold. Black dots mark the start and end times at which FIX concentrations reached the 1% target.

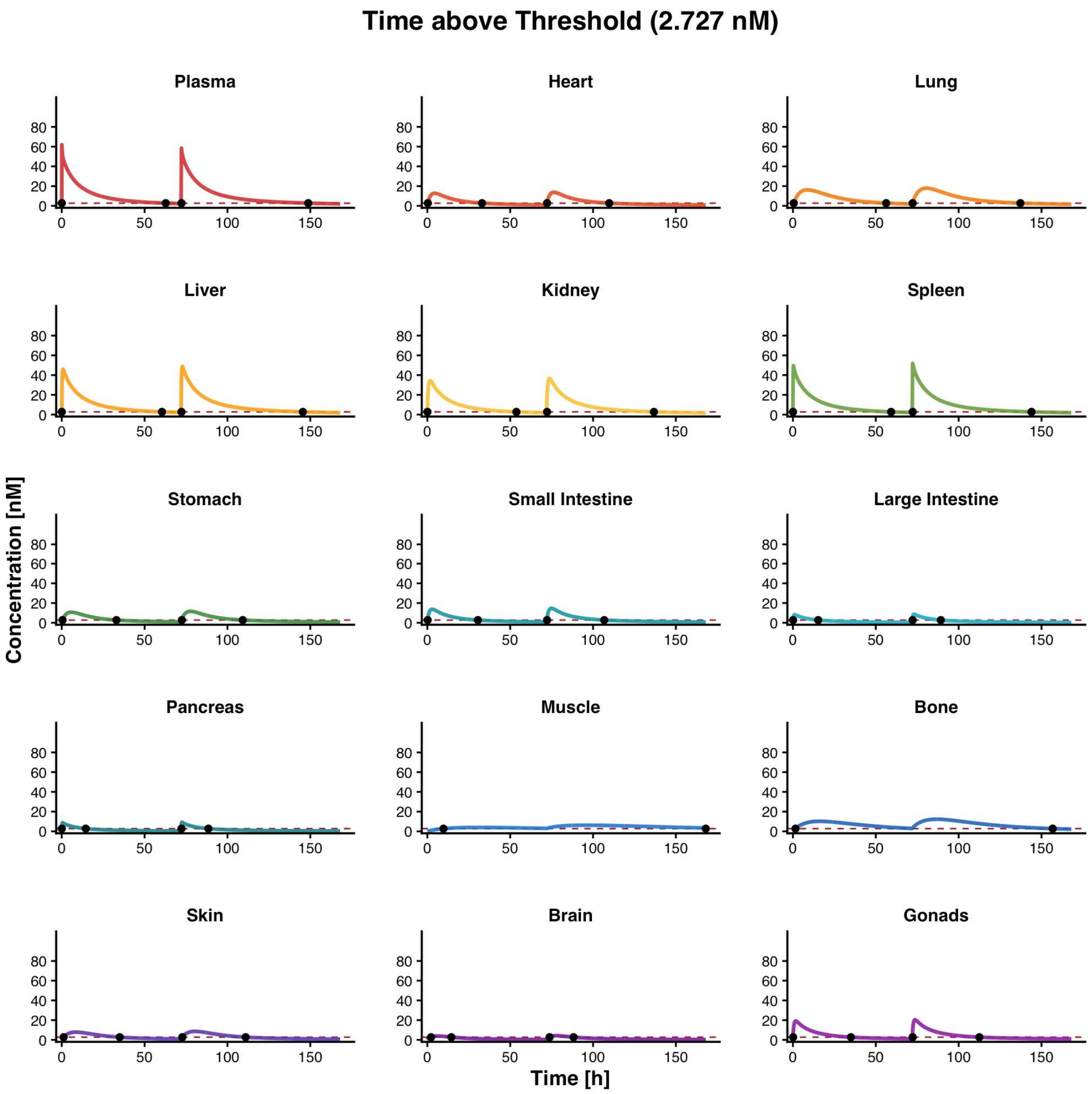

Figure S54. Time intervals during which FIX concentrations exceeded 3% in different tissues after twice-weekly dosing at 50 IU/kg (72h). The y-axis represents FIX concentration (nM), and the x-axis represents time (t). The red line indicates the 3% concentration threshold. Black dots mark the start and end times at which FIX concentrations reached the 3% target.

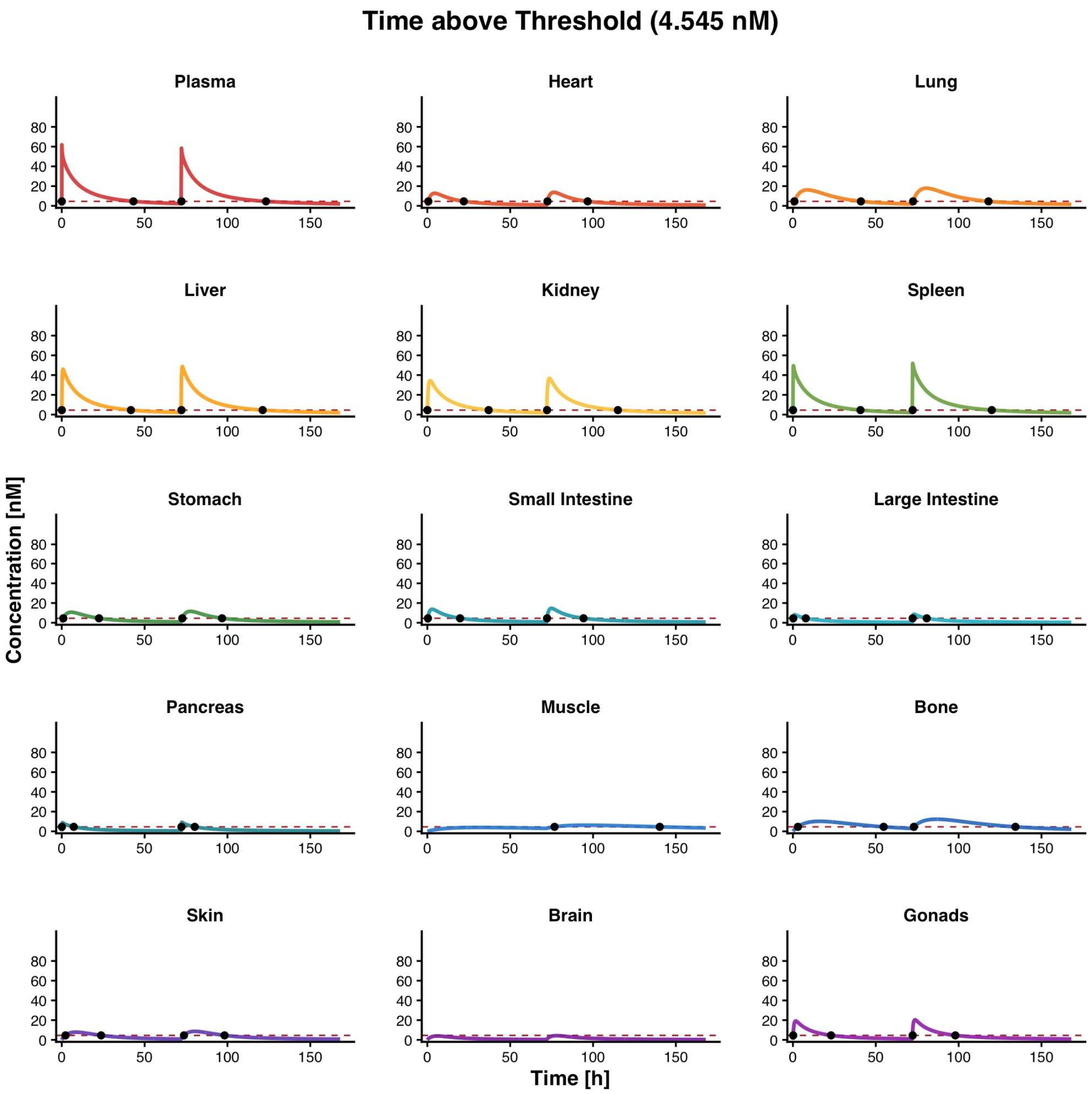

Figure S55. Time intervals during which FIX concentrations exceeded 5% in different tissues after twice-weekly dosing at 50 IU/kg (72h). The y-axis represents FIX concentration (nM), and the x-axis represents time (t). The red line indicates the 5% concentration threshold. Black dots mark the start and end times at which FIX concentrations reached the 5% target.

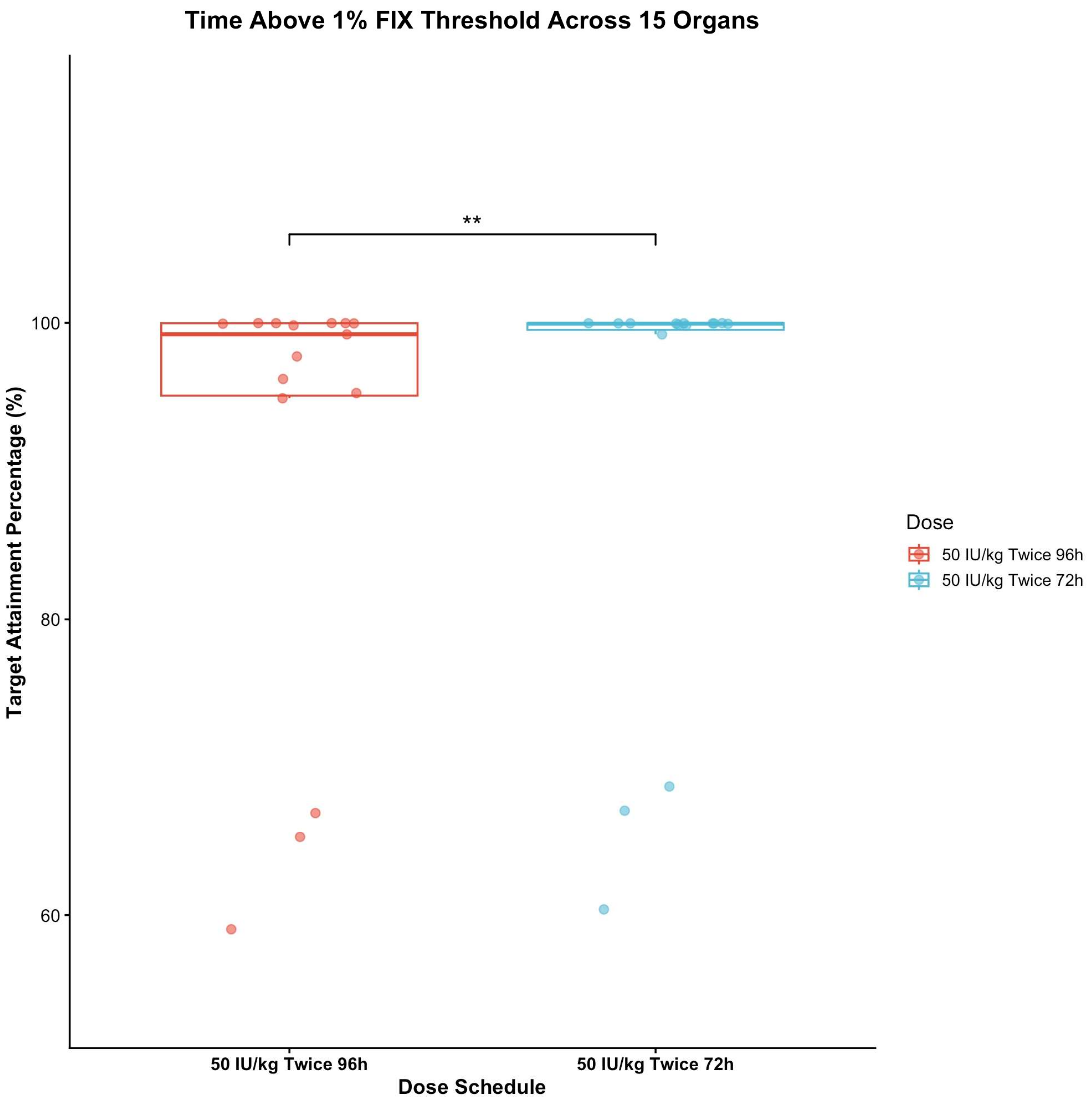

Figure S56. Box plots of target attainment rates (>1%) under different dosing schedules. This figure summarizes differences in the rate of achieving FIX concentrations above 1% across tissues between the two dosing regimens. Red represents the 50 IU/kg twice-weekly (96h) group, and blue represents the 50 IU/kg twice-weekly (72h) group.

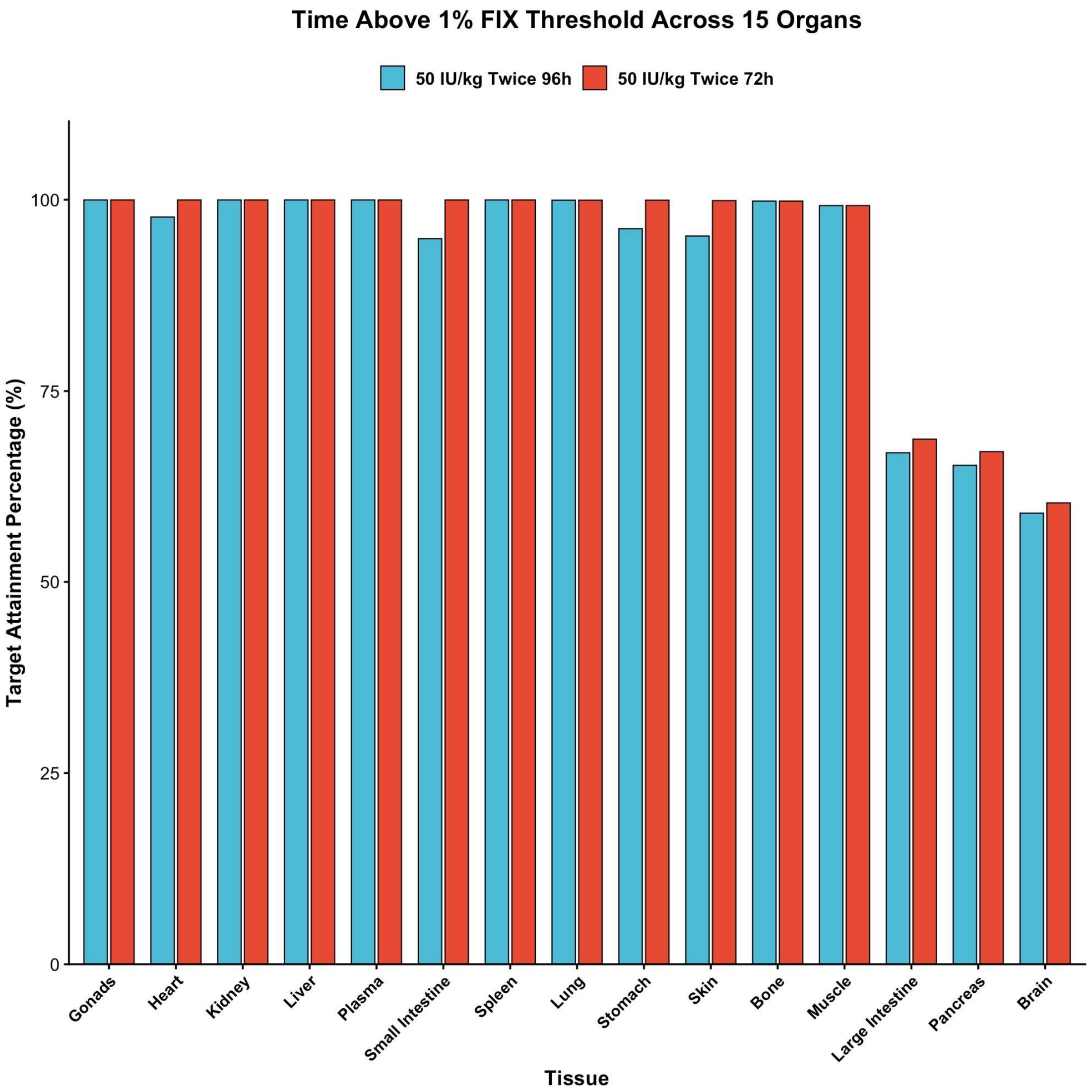

Figure S57. Bar plots of target attainment rates (>1%) under different dosing schedules. This figure shows differences in the rate of achieving FIX concentrations above 1% across tissues under different dosing regimens. Blue represents the 50 IU/kg twice-weekly (96h) group, and red represents the 50 IU/kg twice-weekly (72h) group.

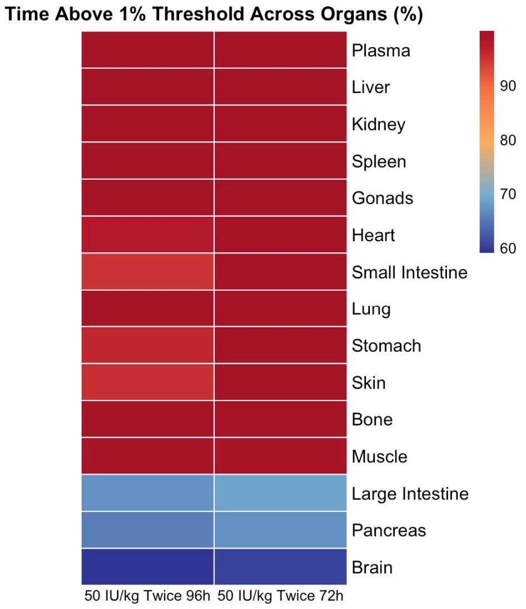

Figure S58. Heatmap of target attainment rates (>1%) under different dosing schedules. This figure shows differences in the rate of achieving FIX concentrations above 1% across tissues under different dosing regimens. Colors range from blue to red, indicating increasing target attainment. From left to right, the panels represent the 50 IU/kg twice-weekly (96h), and 50 IU/kg twice-weekly (72h) groups.

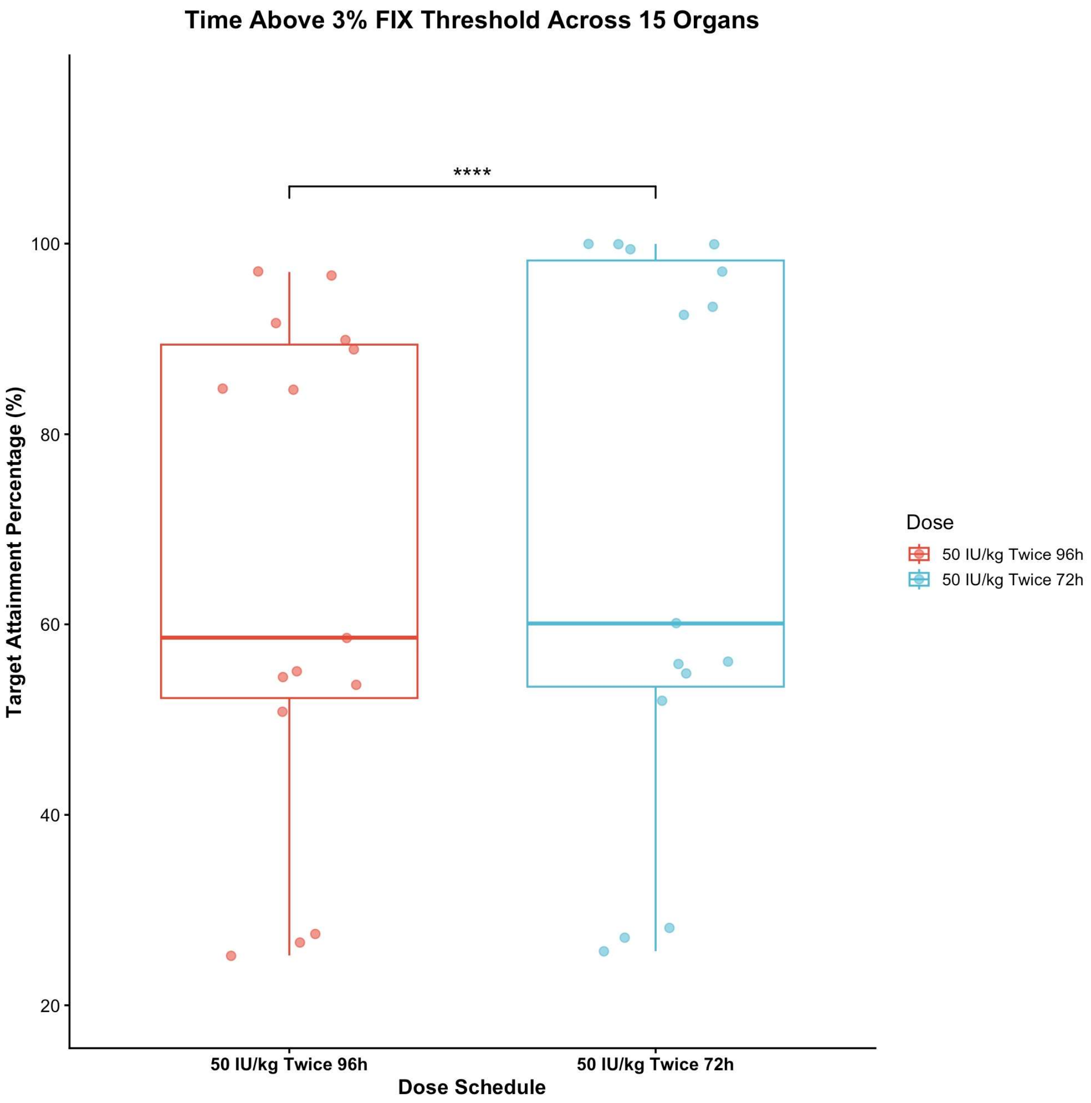

Figure S59. Box plots of target attainment rates (>3%) under different dosing schedules. This figure summarizes differences in the rate of achieving FIX concentrations above 3% across tissues between the two dosing regimens. Red represents the 50 IU/kg twice-weekly (96h) group, and blue represents the 50 IU/kg twice-weekly (72h) group.

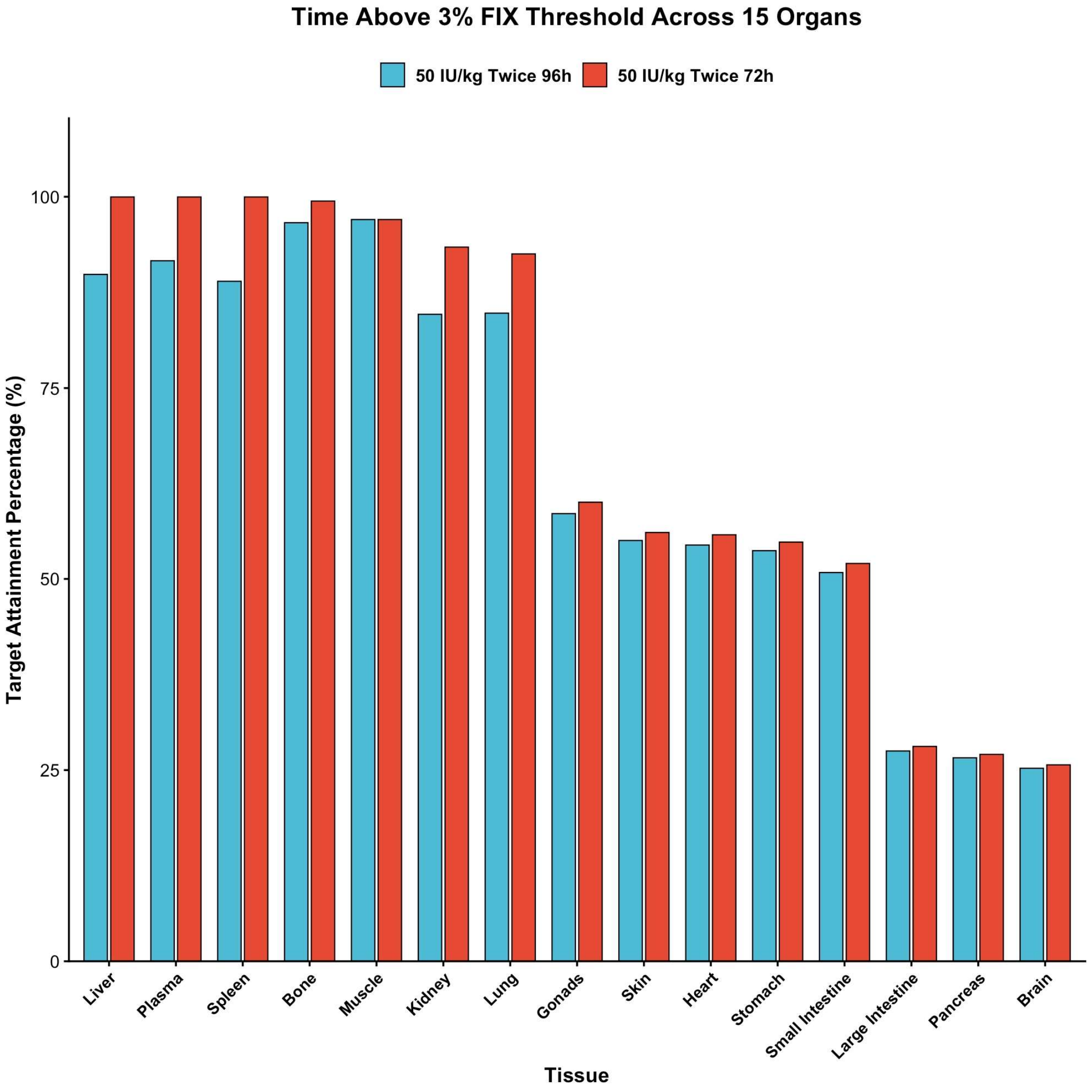

Figure S60. Bar plots of target attainment rates (>3%) under different dosing schedules. This figure shows differences in the rate of achieving FIX concentrations above 3% across tissues under different dosing regimens. Blue represents the 50 IU/kg twice-weekly (96h) group, and red represents the 50 IU/kg twice-weekly (72h) group.

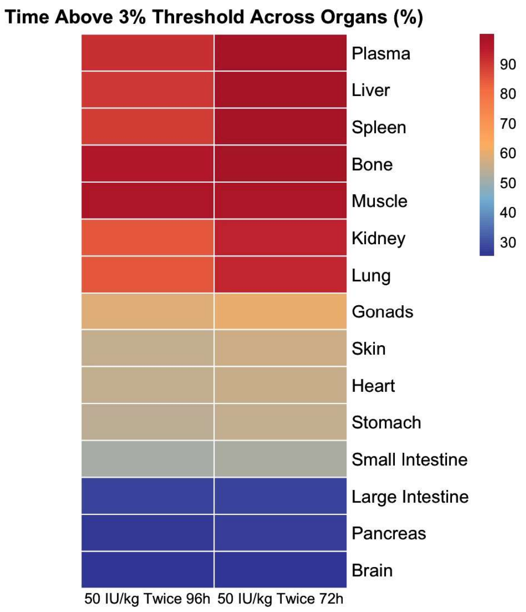

Figure S61. Heatmap of target attainment rates (>3%) under different dosing schedules. This figure shows differences in the rate of achieving FIX concentrations above 3% across tissues under different dosing regimens. Colors range from blue to red, indicating increasing target attainment. From left to right, the panels represent the 50 IU/kg twice-weekly (96h), and 50 IU/kg twice-weekly (72h) groups.

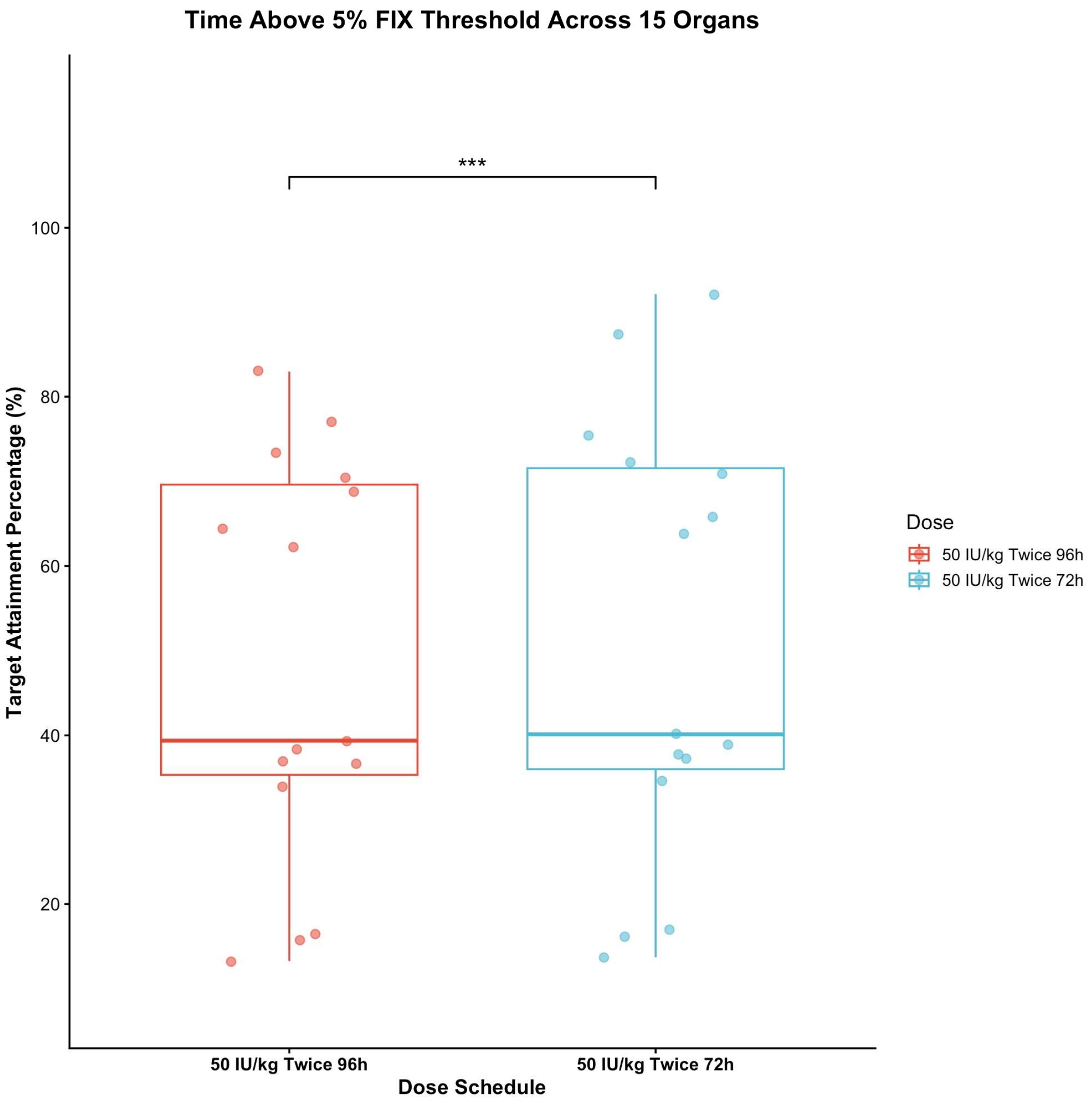

Figure S62. Box plots of target attainment rates (>5%) under different dosing schedules. This figure summarizes differences in the rate of achieving FIX concentrations above 5% across tissues between the two dosing regimens. Red represents the 50 IU/kg twice-weekly (96h) group, and blue represents the 50 IU/kg twice-weekly (72h) group.

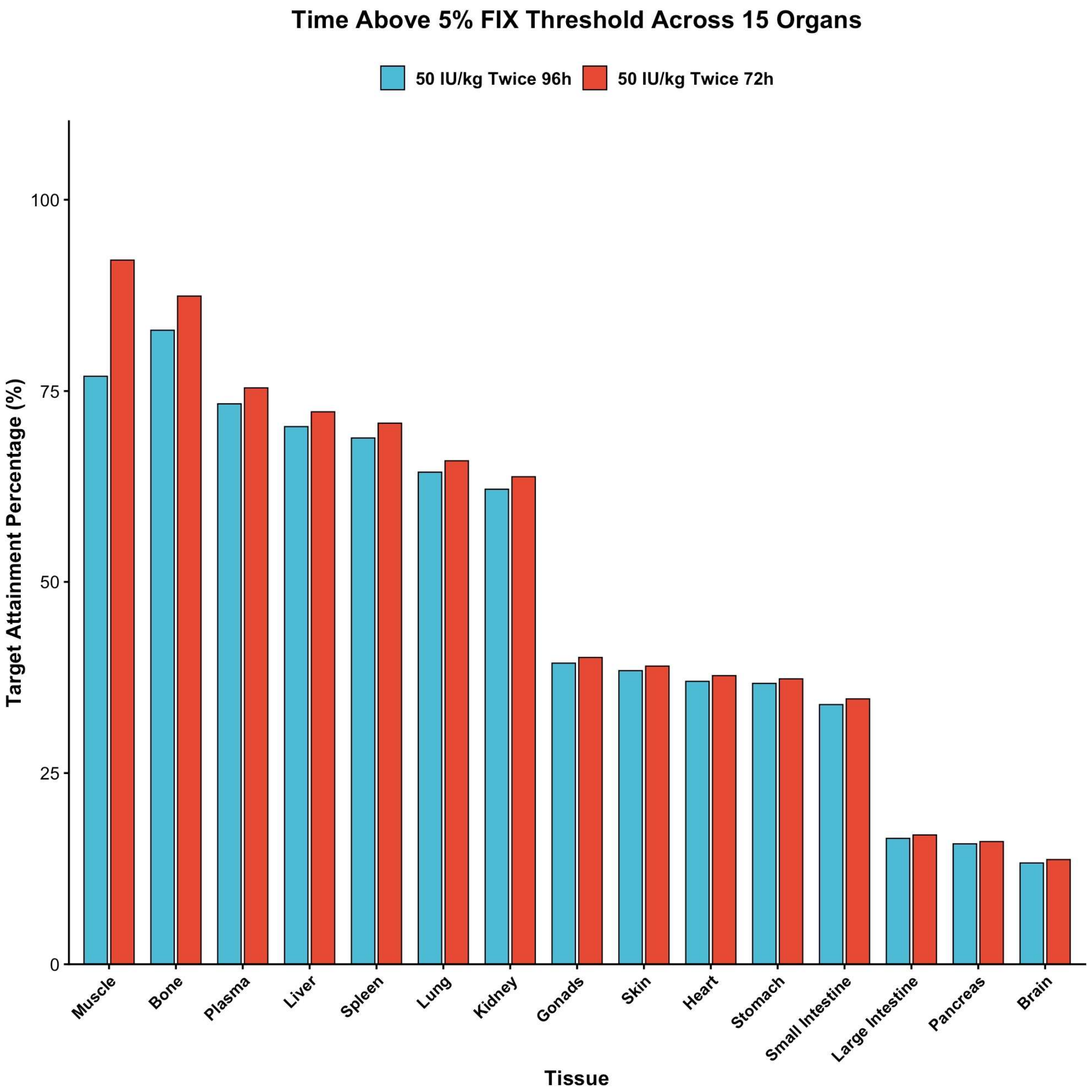

Figure S63. Bar plots of target attainment rates (>5%) under different dosing schedules. This figure shows differences in the rate of achieving FIX concentrations above 5% across tissues under different dosing regimens. Blue represents the 50 IU/kg twice-weekly (96h) group, and red represents the 50 IU/kg twice-weekly (72h) group.

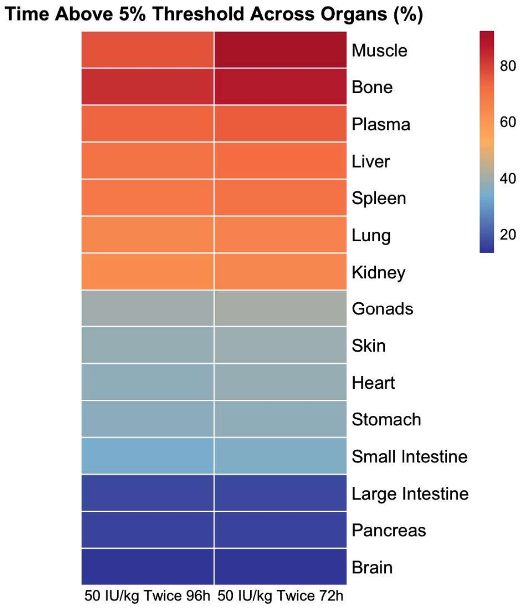

Figure S64. Heatmap of target attainment rates (>5%) under different dosing schedules. This figure shows differences in the rate of achieving FIX concentrations above 5% across tissues under different dosing regimens. Colors range from blue to red, indicating increasing target attainment. From left to right, the panels represent the 50 IU/kg twice-weekly (96h), and 50 IU/kg twice-weekly (72h) groups.

Supplementary Tables

TableS1. Patients' baseline characteristics and demographics

Characters	Mean ± SD/Count	Range
Gender (Male/Female)	20/0	
Age (years)	8.59±4.46	1.42-16.66
Weight (kg)	35.67±18.77	12.00-70.00
Height (cm)	134.30±29.85	107.50-148.00
Blood type（O/A/B/AB）	6/4/9/1	
Endogenous coagulation factor levels (IU/dL)	2.60±1.84	0.80-5.50

Table S2. Quantitative predictive performance of the pediatric FIX PBPK model.

Dataset	N	MPE (%)	MAPE (%)	RMSE (nM)	AAFE	95% PI coverage, n (%)	2-fold error coverage, n (%)
Testing	39	0.02	31.57	9.11	1.421	32 (82.05)	34 (87.18)

Table S3. Plasma pharmacokinetic parameters in adults and children.

Parameters	Age Group				
	1-2 y	2-6 y	6-12 y	12-18 y	Adult
Cmax (nM)	40.20	41.90	63.40	64.30	56.42
AUC (nM*h)	570.00	641.00	853.00	940.00	1188.00
CL (mL/h/kg)	7.02	5.44	5.24	3.03	3.60
Vss (mL/kg)	183.10	172.70	219.50	184.10	163.83
$t_{1/2}$ (h)	56.80	60.90	58.60	65.00	41.36
1% Target Attainment Rate	100.00%	100.00%	100.00%	100.00%	100.00%
3% Target Attainment Rate	66.70%	81.60%	100.00%	100.00%	100.00%

Parameters	Age Group				
	1-2 y	2-6 y	6-12 y	12-18 y	Adult
**5% Target Attainment Rate**	36.70%	43.80%	61.60%	77.30%	75.10%

Table S4. Time above the 1% target for FIX in each tissue in the pediatric model after administration of 50 IU/kg.

Tissue	Start time (h)	End time (h)	Duration (h)	target attainment rates (%)
**Plasma**	0.05	96.00	95.95	99.95
**Liver**	0.05	96.00	95.95	99.95
**Kidney**	0.05	96.00	95.95	99.95
**Spleen**	0.05	96.00	95.95	99.95
**Gonads**	0.05	96.00	95.95	99.95
**Lung**	0.15	96.00	95.85	99.84
**Bone**	0.55	96.00	95.45	99.43
**Muscle**	2.00	96.00	94.00	97.92
**Heart**	0.05	86.00	85.95	89.53
**Stomach**	0.10	84.25	84.15	87.66
**Skin**	0.35	82.5	82.15	85.57
**Small Intestine**	0.05	79.25	79.20	82.50
**Large Intestine**	0.05	46.75	46.70	48.65
**Pancreas**	0.05	43.75	43.70	45.52
**Brain**	0.20	42.25	42.05	43.80

Table S5. Time above the 3% target for FIX in each tissue in the pediatric model after administration of 50 IU/kg.

Tissue	Start time (h)	End time (h)	Duration (h)	target attainment rates (%)
**Muscle**	5.25	96.00	90.75	94.53
**Plasma**	0.05	90.00	89.95	93.70
**Liver**	0.05	83.75	83.70	87.19
**Spleen**	0.05	83.75	83.70	87.19
**Bone**	2.00	81.00	79.00	82.29
**Kidney**	0.05	70.00	69.95	72.86
**Lung**	0.40	55.75	55.35	57.66
**Gonads**	0.05	44.50	44.45	46.30
**Heart**	0.20	32.25	32.05	33.39
**Stomach**	0.25	31.50	31.25	32.55
**Skin**	2.00	33.00	31.00	32.29

**Small Intestine**	0.10	28.75	28.65	29.84
**Large Intestine**	0.05	14.50	14.45	15.05
**Pancreas**	0.05	13.00	12.95	13.49
**Brain**	0.70	13.00	12.30	12.81

Table S6. Time above the 5% target for FIX in each tissue in the pediatric model after administration of 50 IU/kg.

Tissue	Start time (h)	End time (h)	Duration (h)	target attainment rates (%)
**Plasma**	0.05	59.25	59.20	61.67
**Spleen**	0.05	54.25	54.20	56.46
**Liver**	0.10	54.25	54.15	56.41
**Bone**	3.50	56.00	52.50	54.69
**Muscle**	11.50	60.50	49.00	51.04
**Kidney**	0.05	44.00	43.95	45.78
**Lung**	0.75	35.75	35.00	36.46
**Gonads**	0.05	27.00	26.95	28.07
**Heart**	0.35	19.00	18.65	19.43
**Skin**	2.25	20.50	18.25	19.01
**Stomach**	0.50	18.50	18.00	18.75
**Small Intestine**	0.20	16.50	16.30	16.98
**Large Intestine**	0.10	5.75	5.65	5.89
**Pancreas**	0.10	4.50	4.40	4.58
**Brain**	2.25	4.50	2.25	2.34

Table S7. Time above the 1% target for FIX in each tissue in the pediatric model after administration of 75 IU/kg.

Tissue	Start time (h)	End time (h)	Duration (h)	target attainment rates (%)
**Gonads**	0.05	96.00	95.95	99.95
**Heart**	0.05	96.00	95.95	99.95
**Kidney**	0.05	96.00	95.95	99.95
**Liver**	0.05	96.00	95.95	99.95
**Plasma**	0.05	96.00	95.95	99.95
**Small Intestine**	0.05	96.00	95.95	99.95
**Spleen**	0.05	96.00	95.95	99.95
**Stomach**	0.05	96.00	95.95	99.95

**Lung**	0.10	96.00	95.90	99.90
**Skin**	0.20	96.00	95.80	99.79
**Bone**	0.35	96.00	95.65	99.64
**Muscle**	0.90	96.00	95.10	99.06
**Large Intestine**	0.05	67.50	67.45	70.26
**Pancreas**	0.05	63.50	63.45	66.09
**Brain**	0.15	61.25	61.10	63.65

Table S8. Time above the 3% target for FIX in each tissue in the pediatric model after administration of 75 IU/kg.

Tissue	Start time (h)	End time (h)	Duration (h)	target attainment rates (%)
**Liver**	0.05	96.00	95.95	99.95
**Plasma**	0.05	96.00	95.95	99.95
**Spleen**	0.05	96.00	95.95	99.95
**Kidney**	0.05	95.75	95.70	99.69
**Bone**	2.00	96.00	94.00	97.92
**Muscle**	3.25	96.00	92.75	96.61
**Lung**	0.25	78.25	78.00	81.25
**Gonads**	0.05	64.75	64.70	67.40
**Heart**	0.15	47.50	47.35	49.32
**Stomach**	0.15	46.25	46.10	48.02
**Skin**	0.70	46.75	46.05	47.97
**Small Intestine**	0.10	42.75	42.65	44.43
**Large Intestine**	0.05	23.50	23.45	24.43
**Pancreas**	0.05	21.75	21.70	22.60
**Brain**	0.40	21.25	20.85	21.72

Table S9. Time above the 5% target for FIX in each tissue in the pediatric model after administration of 75 IU/kg.

Tissue	Start time (h)	End time (h)	Duration (h)	target attainment rates (%)
**Muscle**	6.00	93.00	87.00	90.63
**Plasma**	0.05	83.50	83.45	86.93
**Liver**	0.05	77.25	77.20	80.42
**Spleen**	0.05	77.25	77.20	80.42
**Bone**	2.00	75.50	73.50	76.56
**Kidney**	0.05	64.00	63.95	66.61
**Lung**	0.45	51.00	50.55	52.66
**Gonads**	0.05	40.25	40.20	41.88
**Heart**	0.20	29.00	28.80	30.00
**Skin**	2.00	30.25	28.25	29.43
**Stomach**	0.30	28.50	28.20	29.38
**Small Intestine**	0.15	26.00	25.85	26.93

**Large Intestine**	0.05	12.50	12.45	12.97
**Pancreas**	0.10	11.00	10.90	11.35
**Brain**	0.85	11.25	10.40	10.83

Table S10. Time above the 1% target for FIX in each tissue in the pediatric model after administration of 100 IU/kg.

Tissue	Start time (h)	End time (h)	Duration (h)	target attainment rates (%)
**Gonads**	0.05	96.00	95.95	99.95
**Heart**	0.05	96.00	95.95	99.95
**Kidney**	0.05	96.00	95.95	99.95
**Liver**	0.05	96.00	95.95	99.95
**Plasma**	0.05	96.00	95.95	99.95
**Small Intestine**	0.05	96.00	95.95	99.95
**Spleen**	0.05	96.00	95.95	99.95
**Stomach**	0.05	96.00	95.95	99.95
**Lung**	0.10	96.00	95.90	99.90
**Skin**	0.15	96.00	95.85	99.84
**Bone**	0.25	96.00	95.75	99.74
**Muscle**	0.65	96.00	95.35	99.32
**Large Intestine**	0.05	85.25	85.20	88.75
**Pancreas**	0.05	80.75	80.70	84.06
**Brain**	0.10	78.00	77.90	81.15

Table S11. Time above the 3% target for FIX in each tissue in the pediatric model after administration of 100 IU/kg.

Tissue	Start time (h)	End time (h)	Duration (h)	target attainment rates (%)
**Kidney**	0.05	96.00	95.95	99.95
**Liver**	0.05	96.00	95.95	99.95
**Plasma**	0.05	96.00	95.95	99.95
**Spleen**	0.05	96.00	95.95	99.95
**Lung**	0.20	96.00	95.80	99.79
**Bone**	0.80	96.00	95.20	99.17
**Muscle**	2.25	96.00	93.75	97.66
**Gonads**	0.05	82.25	82.20	85.63
**Heart**	0.10	61.50	61.40	63.96
**Stomach**	0.15	60.25	60.10	62.60
**Skin**	0.50	59.50	59.00	61.46
**Small Intestine**	0.05	56.00	55.95	58.28

**Large Intestine**	0.05	31.50	31.45	32.76
**Pancreas**	0.05	29.25	29.20	30.42
**Brain**	0.30	28.75	28.45	29.64

Table S12. Time above the 5% target for FIX in each tissue in the pediatric model after administration of 100 IU/kg.

Tissue	Start time (h)	End time (h)	Duration (h)	Target attainment rates (%)
**Plasma**	0.05	96.00	95.95	99.95
**Liver**	0.05	95.75	95.70	99.69
**Spleen**	0.05	95.75	95.70	99.69
**Muscle**	4.00	96.00	92.00	95.83
**Bone**	2.00	91.00	89.00	92.71
**Kidney**	0.05	81.25	81.20	84.58
**Lung**	0.35	65.25	64.90	67.60
**Gonads**	0.05	53.00	52.95	55.16
**Heart**	0.15	38.50	38.35	39.95
**Skin**	0.90	38.75	37.85	39.43
**Stomach**	0.20	37.50	37.30	38.85
**Small Intestine**	0.10	34.50	34.40	35.83
**Large Intestine**	0.05	18.25	18.20	18.96
**Pancreas**	0.05	16.75	16.70	17.40
**Brain**	0.55	16.50	15.95	16.61

Table S13. Statistical comparison of target attainment metrics between different dose groups.

Target attainment	Group1	Group2	p
	50 IU/kg	75 IU/kg	0.018
**>1%**	50 IU/kg	100 IU/kg	0.018
	75 IU/kg	100 IU/kg	0.108
	50 IU/kg	75 IU/kg	< 0.001
**>3%**	50 IU/kg	100 IU/kg	0.002
	75 IU/kg	100 IU/kg	0.008
	50 IU/kg	75 IU/kg	< 0.001
**>5%**	50 IU/kg	100 IU/kg	< 0.001
	75 IU/kg	100 IU/kg	< 0.001

Table S14. Time above the 1% target for FIX in each tissue in the pediatric model after administration of 50IU/kg twice weekly

Tissue	Start time (h)	End time (h)	Duration (h)	Target attainment rates (%)
**Plasma**	0.05	168.00	167.95	99.97
**Liver**	0.05	168.00	167.95	99.97
**Kidney**	0.05	168.00	167.95	99.97
**Spleen**	0.05	168.00	167.95	99.97
**Gonads**	0.05	168.00	167.95	99.97
**Lung**	0.10	168.00	167.90	99.94
**Bone**	0.30	168.00	167.70	99.82
**Muscle**	1.30	168.00	166.70	99.23
**Heart**	0.05<br>96.25	92.50<br>168.00	164.20	97.74
**Stomach**	0.10<br>96.25	90.00<br>168.00	161.65	96.22
**Skin**	0.20<br>96.25	88.50<br>168.00	160.05	95.27
**Small Intestine**	0.05<br>96.25	87.75<br>168.00	159.45	94.91
**Large Intestine**	0.05<br>96.25	52.50<br>156.25	112.45	66.93
**Pancreas**	0.05<br>96.25	51.25<br>154.75	109.70	65.30
**Brain**	0.30<br>96.25	47.00<br>148.75	99.20	59.05

Table S15. Time above the 3% target for FIX in each tissue in the pediatric model after administration of 50IU/kg twice weekly

Tissue	Start time (h)	End time (h)	Duration (h)	Target attainment rates (%)
**Muscle**	5.00	168.00	163.00	97.02
**Bone**	0.95<br>96.25	91.50<br>168.00	162.30	96.61
**Plasma**	0.05<br>96.25	82.25<br>168.00	153.95	91.64
**Liver**	0.05<br>96.25	79.25<br>168.00	150.95	89.85
**Spleen**	0.05<br>96.25	77.75<br>168.00	149.45	88.96
**Lung**	0.30<br>96.25	71.00<br>168.00	142.45	84.79
**Kidney**	0.05<br>96.25	70.50<br>168.00	142.20	84.64

**Gonads**	0.05<br>96.25	46.25<br>148.50	98.45	58.60
**Skin**	0.70<br>96.50	44.75<br>145.00	92.55	55.09
**Heart**	0.20<br>96.25	43.50<br>144.50	91.55	54.49
**Stomach**	0.30<br>96.25	43.00<br>143.75	90.20	53.69
**Small Intestine**	0.10<br>96.25	40.50<br>141.25	85.40	50.83
**Large Intestine**	0.05<br>96.25	22.25<br>120.25	46.20	27.50
**Pancreas**	0.05<br>96.25	21.50<br>119.50	44.70	26.61
**Brain**	1.10<br>97.00	21.50<br>119.00	42.40	25.24

Table S16. Time above the 5% target for FIX in each tissue in the pediatric model after administration of 50IU/kg twice weekly

Tissue	Start time (h)	End time (h)	Duration (h)	Target attainment rates (%)
**Bone**	1.65<br>96.75	69.75<br>168.00	139.35	82.95
**Muscle**	11.50<br>97.75	70.50<br>168.00	129.25	76.93
**Plasma**	0.05<br>96.25	57.25<br>162.25	123.20	73.33
**Liver**	0.05<br>96.25	55.00<br>159.50	118.20	70.36
**Spleen**	0.05<br>96.25	54.00<br>158.00	115.70	68.87
**Lung**	0.55<br>96.50	51.75<br>153.50	108.20	64.40
**Kidney**	0.05<br>96.25	49.00<br>151.75	104.45	62.17
**Gonads**	0.10<br>96.25	31.50<br>131.00	66.15	39.38
**Skin**	1.25<br>97.00	32.25<br>130.50	64.50	38.39
**Heart**	0.35	30.00	62.15	36.99

	96.25	128.75		
**Stomach**	0.55<br>96.50	30.00<br>128.75	61.70	36.73
**Small Intestine**	0.20<br>96.25	27.50<br>126.00	57.05	33.96
**Large Intestine**	0.10<br>96.25	13.50<br>110.50	27.65	16.46
**Pancreas**	0.05<br>96.25	12.75<br>110.00	26.45	15.74
**Brain**	2.50<br>98.25	13.00<br>110.00	22.25	13.24

Table S17. Time above the 1% target for FIX in each tissue in the pediatric model after administration of 100IU/kg Once weekly

Tissue	Start time (h)	End time (h)	Duration (h)	Target attainment rates (%)
**Plasma**	0.05	168.00	167.95	99.97
**Liver**	0.05	168.00	167.95	99.97
**Spleen**	0.05	168.00	167.95	99.97
**Kidney**	0.05	168.00	167.95	99.97
**Lung**	0.10	168.00	167.90	99.94
**Bone**	0.25	168.00	167.75	99.85
**Muscle**	0.65	168.00	167.35	99.61
**Gonads**	0.05	158.00	157.95	94.02
**Heart**	0.05	133.25	133.20	79.29
**Stomach**	0.05	131.25	131.20	78.10
**Skin**	0.15	129.00	128.85	76.70
**Small Intestine**	0.05	126.00	125.95	74.97
**Large intestine**	0.05	85.25	85.20	50.71
**Pancreas**	0.05	80.75	80.70	48.04
**Brain**	0.10	78.00	77.90	46.37

Table S18. Time above the 3% target for FIX in each tissue in the pediatric model after administration of 100IU/kg Once weekly

Tissue	Start time (h)	End time (h)	Duration (h)	Target attainment rates (%)
**Muscle**	2.25	152.00	149.75	89.14
**Plasma**	0.05	138.00	137.95	82.11
**Liver**	0.05	131.00	130.95	77.95

**Spleen**	0.05	131.00	130.95	77.95
**Bone**	0.80	122.50	121.70	72.44
**Kidney**	0.05	115.50	115.45	68.72
**Lung**	0.20	96.50	96.30	57.32
**Gonads**	0.05	82.25	82.20	48.93
**Heart**	0.10	61.50	61.40	36.55
**Stomach**	0.15	60.25	60.10	35.77
**Skin**	0.50	59.50	59.00	35.12
**Small Intestine**	0.05	56.00	55.95	33.30
**Large Intestine**	0.05	31.50	31.45	18.72
**Pancreas**	0.05	29.25	29.20	17.38
**Brain**	0.30	28.75	28.45	16.93

Table S19. Time above the 5% target for FIX in each tissue in the pediatric model after administration of 100IU/kg Once weekly

Tissue	Start time (h)	End time (h)	Duration (h)	Target attainment rates (%)
**Muscle**	4.00	114.50	110.50	65.77
**Plasma**	0.05	102.50	102.45	60.98
**Liver**	0.05	95.75	95.70	56.96
**Spleen**	0.05	95.75	95.70	56.96
**Bone**	2.00	91.00	89.00	52.98
**Kidney**	0.05	81.25	81.20	48.33
**Lung**	0.35	65.25	64.90	38.63
**Gonads**	0.05	53.00	52.95	31.52
**Skin**	0.90	38.75	37.85	22.53
**Heart**	0.15	38.50	38.35	22.83
**Stomach**	0.20	37.50	37.30	22.20
**Small Intestine**	0.10	34.50	34.40	20.48
**Large Intestine**	0.05	18.25	18.20	10.83
**Pancreas**	0.05	16.75	16.70	9.94
**Brain**	0.55	16.50	15.95	9.49

Table S20. Statistical comparison of target attainment between increased dosing frequency and increased single-dose strategies under the same total weekly dose

Target attainment	Group1	Group2	p
**>1%**	50 IU/kg Twice Weekly	100 IU/kg Once Weekly	0.0039
**>3%**	50 IU/kg Twice Weekly	100 IU/kg Once Weekly	< 0.001
**>5%**	50 IU/kg Twice Weekly	100 IU/kg Once Weekly	< 0.001

Table S21. Time above the 1% target for FIX in each tissue in the pediatric model after administration of 50IU/kg Three Times weekly

Tissue	Start time (h)	End time (h)	Duration (h)	Target attainment rates (%)
**Kidney**	0.05	168.00	167.95	99.97
**Liver**	0.05	168.00	167.95	99.97
**Pancreas**	0.05	168.00	167.95	99.97
**Plasma**	0.05	168.00	167.95	99.97
**Small Intestine**	0.05	168.00	167.95	99.97
**Spleen**	0.05	168.00	167.95	99.97
**Heart**	0.10	168.00	167.90	99.94
**Lung**	0.10	168.00	167.90	99.94
**Gonads**	0.15	168.00	167.85	99.91
**Stomach**	0.15	168.00	167.85	99.91
**Skin**	0.30	168.00	167.70	99.82
**Bone**	0.35	168.00	167.65	99.79
**Muscle**	0.80	168.00	167.20	99.52
**Large Intestine**	0.05<br>56.25	53.75<br>168.00	165.45	98.48
**Brain**	0.60<br>56.25<br>112.25	40.75<br>106.25<br>166.50	144.40	85.95

Table S22. Time above the 3% target for FIX in each tissue in the pediatric model after administration of 50IU/kg Three Times weekly

Tissue	Start time (h)	End time (h)	Duration (h)	Target attainment rates (%)
**Kidney**	0.05	168.00	167.95	99.97
**Liver**	0.05	168.00	167.95	99.97
**Plasma**	0.05	168.00	167.95	99.97
**Spleen**	0.05	168.00	167.95	99.97
**Lung**	0.35	168.00	167.65	99.79
**Skin**	1.05	168.00	166.95	99.38
**Bone**	1.10	168.00	166.90	99.35
**Muscle**	3.00	168.00	165.00	98.21
**Heart**	0.25<br>56.25	44.00<br>168.00	155.50	92.56
**Stomach**	0.40<br>56.25<br>112.25	42.50<br>109.50<br>168.00	151.10	89.94
**Small Intestine**	0.15<br>56.25	40.25<br>109.00	148.60	88.45

	112.25	168.00		
Gonads	0.45<br>56.25<br>112.25	32.00<br>95.50<br>155.00	113.55	67.59
Pancreas	0.05<br>56.25<br>112.25	21.00<br>82.00<br>140.50	74.95	44.61
Large Intestine	0.10<br>56.25<br>112.25	19.50<br>79.75<br>137.75	68.40	40.71
Brain	2.50<br>57.75<br>113.50	17.00<br>76.00<br>133.00	52.25	31.10

Table S23. Time above the 5% target for FIX in each tissue in the pediatric model after administration of 50IU/kg Three Times weekly

Tissue	Start time (h)	End time (h)	Duration (h)	Target attainment rates (%)
Bone	2.00	168.00	166.00	98.81
Lung	0.60<br>56.25	52.00<br>168.00	163.15	97.11
Muscle	6.50<br>56.25	55.00<br>168.00	160.25	95.39
Plasma	0.05<br>56.25	46.75<br>168.00	158.45	94.32
Liver	0.05<br>56.25	45.00<br>168.00	156.70	93.27
Skin	1.95<br>56.50	47.00<br>168.00	156.55	93.18
Spleen	0.05<br>56.25	44.00<br>168.00	155.70	92.68
Kidney	0.05<br>56.25<br>112.25	40.00<br>109.00<br>168.00	148.45	88.36
Heart	0.40<br>56.25<br>112.25	29.00<br>91.50<br>150.50	102.10	60.77
Stomach	0.75<br>56.50<br>112.25	29.00<br>90.50<br>149.00	99.00	58.93

	0.25	25.75		
**Small Intestine**	56.25	88.00	91.75	54.61
	112.25	146.75		
	0.80	21.75		
**Gonads**	56.50	81.25	71.95	42.83
	112.50	138.75		
	0.10	13.00		
**Pancreas**	56.25	71.25	43.90	26.13
	112.25	128.25		
	0.15	12.00		
**Large Intestine**	56.25	70.00	40.10	23.87
	112.25	126.75		
	0.20	12.00		
**Brain**	56.25	70.00	40.05	23.84
	112.25	126.75		

Table S24. Statistical comparison metrics between different dosing frequency groups

Target attainment	Group1	Group2	p
	50 IU/kg Once Weekly	50 IU/kg Twice Weekly	< 0.001
**>1%**	50 IU/kg Once Weekly	50 IU/kg Three Times Weekly	< 0.001
	50 IU/kg Twice Weekly	50 IU/kg Three Times Weekly	0.018
	50 IU/kg Once Weekly	50 IU/kg Twice Weekly	< 0.001
**>3%**	50 IU/kg Once Weekly	50 IU/kg Three Times Weekly	0.002
	50 IU/kg Twice Weekly	50 IU/kg Three Times Weekly	< 0.001
	50 IU/kg Once Weekly	50 IU/kg Twice Weekly	< 0.001
**>5%**	50 IU/kg Once Weekly	50 IU/kg Three Times Weekly	< 0.001
	50 IU/kg Twice Weekly	50 IU/kg Three Times Weekly	< 0.001

Table S25. Statistical comparison metrics between different dosing schedule groups.

Target attainment	Group	Mean Target attainment rates %	p
**>1%**	50 IU/kg Twice Weekly (96h)	91.62	0.0059
	50 IU/kg Twice Weekly (72h)	92.99	
**>3%**	50 IU/kg Twice Weekly (96h)	65.70	< 0.001
	50 IU/kg Twice Weekly (72h)	69.47	
**>5%**	50 IU/kg Twice Weekly (96h)	48.66	< 0.001
	50 IU/kg Twice Weekly (72h)	50.88	

Reference

1. Niederalt C, Kuepfer L, Solodenko J, et al. A generic whole body physiologically based pharmacokinetic model for therapeutic proteins in PK-Sim. *J Pharmacokinet Pharmacodyn*. 2018;45(2):235-257. doi:10.1007/s10928-017-9559-4

2. Najjar A, Hamadeh A, Krause S, Schepky A, Edginton A. Global sensitivity analysis of Open Systems Pharmacology Suite physiologically based pharmacokinetic models. *CPT Pharmacomet Syst Pharmacol*. 2024;13(12):2052-2067. doi:10.1002/psp4.13256
